# Synthesis and HDAC inhibitory activity of pyrimidine-based hydroxamic acids

**DOI:** 10.3762/bjoc.18.84

**Published:** 2022-07-13

**Authors:** Virginija Jakubkiene, Gabrielius Ernis Valiulis, Markus Schweipert, Asta Zubriene, Daumantas Matulis, Franz-Josef Meyer-Almes, Sigitas Tumkevicius

**Affiliations:** 1 Department of Organic Chemistry, Faculty of Chemistry and Geosciences, Vilnius University, Naugarduko 24, 03225 Vilnius, Lithuania; 2 Department of Chemical Engineering and Biotechnology, University of Applied Sciences, Stephanstr. 7, 64295 Darmstadt, Germany; 3 Department of Biothermodynamics and Drug Design, Institute of Biotechnology, Life Sciences Center, Vilnius University, Sauletekio 7, 10257 Vilnius, Lithuania

**Keywords:** alkylation, aminolysis, HDAC inhibitors, hydroxamic acid, pyrimidine

## Abstract

Histone deacetylases (HDACs) play an essential role in the transcriptional regulation of cells through the deacetylation of nuclear histone and non-histone proteins and are promising therapeutic targets for the treatment of various diseases. Here, the synthesis of new compounds in which a hydroxamic acid residue is attached to differently substituted pyrimidine rings via a methylene group bridge of varying length as potential HDAC inhibitors is described. The target compounds were obtained by alkylation of 2-(alkylthio)pyrimidin-4(3*H*)-ones with ethyl 2-bromoethanoate, ethyl 4-bromobutanoate, or methyl 6-bromohexanoate followed by aminolysis of the obtained esters with hydroxylamine. Oxidation of the 2-methylthio group to the methylsulfonyl group and following treatment with amines resulted in the formation of the corresponding 2-amino-substituted derivatives, the ester group of which reacted with hydroxylamine to give the corresponding hydroxamic acids. The synthesized hydroxamic acids were tested as inhibitors of the HDAC4 and HDAC8 isoforms. Among the synthesized pyrimidine-based hydroxamic acids *N*-hydroxy-6-[6-methyl-2-(methylthio)-5-propylpyrimidin-4-yloxy]hexanamide was found to be the most potent inhibitor of both the HDAC4 and HDAC8 isoforms, with an IC_50_ of 16.6 µM and 1.2 µM, respectively.

## Introduction

**Figure 1:**
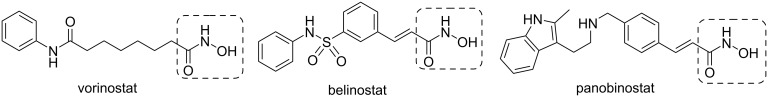
FDA-approved HDAC inhibitors with a hydroxamic acid moiety.

Histone deacetylases (HDACs) are a family of intracellular proteins responsible for removing acetyl groups in histones. This function is essential in the transcription of DNA, as histones with acetyl groups do not interact as strongly with DNA, which opens the chromatin for transcription. HDACs also interact with other cellular proteins to regulate vital functions such as cell differentiation or apoptosis. They have also been shown to play a significant role in pathologies such as cancer, neurodegenerative diseases, and metabolic disorders [1–2]. HDACs are structurally divided into 18 isoforms, which are grouped into 4 classes. The 11 isoforms belonging to classes I, II, and IV are dependent on the Zn^2+^ ion in their catalytic site, while the remaining 7 isoforms of class III, known as sirtuins, are dependent on the NAD^+^ coenzyme [3–4]. According to current knowledge, HDAC inhibitors usually have several structural subunits: a zinc chelating group, a hydrophobic linker, and a hydrophobic (usually aromatic) cap [1–2,5]. One of the most commonly used zinc chelating groups in HDACs inhibitors is a hydroxamic acid moiety (–CONHOH) [6–15]. The ability of hydroxamic acids to form chelates with various metal cations, including the Zn^2+^ ion found in the catalytic center of most HDAC proteins, gives them good biological activity in inhibiting these proteins. To date, three HDAC inhibitor drugs containing this functional group in their structure have been approved [6–7] (Figure 1).

However, the main disadvantage of many HDAC inhibitors is their low selectivity: most of them interact with all HDAC isoforms of groups I, II, and IV. Thus, efforts are currently underway to synthesize inhibitors selective for certain HDAC isoforms [2]. On the other hand, pyrimidines represent an important group of heterocyclic compounds exhibiting a broad spectrum of biological activity [16–20]. Pyrimidine-based hydroxamic acids with HDAC inhibitory activity have also been described [21–22].

Herein, we report on the synthesis of novel pyrimidine derivatives in which the hydroxamic acid fragment is linked to a variably substituted pyrimidine moiety via a methylene group bridge of varying length and the evaluation of their HDAC inhibitory activity.

## Results and Discussion

### Chemistry

**Scheme 1:**
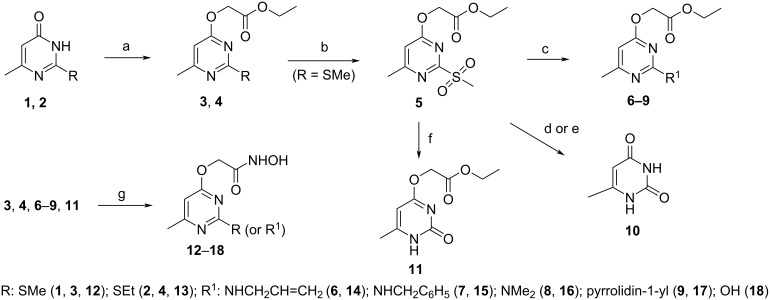
Synthesis of compounds **3**–**18**. Reagents and conditions: (a) ethyl 2-bromoethanoate, TBAB, TEA, 50–60 °C, 0.5 h; (b) oxone, DMF, 40 °C, 0.5 h; (c) corresponding amine, DMSO, 50–70 °C, 0.5 h; (d) H_2_O, DMSO, 100 °C, 0.5 h; (e) H_2_O, reflux, 1 h; (f) aqueous NaOH, dioxane, rt, 12 h, then, conc. HCl to pH 2; (g) NH_2_OH**^.^**HCl, KOH, H_2_O (H_2_O/MeOH for **7**), 0–5 °C, 1–4 h (rt, 96 h for **7**), then, conc. HCl to pH 5–6.

Commonly, hydroxamic acids (*N*-hydroxyamides) are prepared by coupling activated carboxylic acids with *O*-protected hydroxylamine [23–25] or by treatment of esters with hydroxylamine [26–30]. In this work, the pyrimidine-based hydroxamic acids were synthesized by aminolysis of the corresponding esters. The required esters **3** and **4** were obtained by alkylation of pyrimidinones **1** and **2** with ethyl 2-bromoethanoate in triethylamine in the presence of tetrabutylammonium bromide at 50–60 °C in 73% and 70% yields, respectively (Scheme 1).

In order to functionalize the 2nd position of the pyrimidine ring, the 2-methylthio group of compound **3** was oxidized to the better leaving 2-methylsulfonyl group. In our previous work [31] we investigated the oxidation of some 2-methylthiopyrimidines with *m*-CPBA and oxone and found that oxone gave better results, so we have chosen it for this reaction. Thus, heating compound **3** in dimethylformamide at 40 °C for 0.5 h with oxone gave compound **5** in 80% yield. In the NMR spectra of compound **5**, the peaks of the methylsulfonyl group are downfield shifted by 0.8 ppm and 24.8 ppm in the ^1^H and ^13^C NMR spectra, respectively, in comparison with the signals of the methylthio group of compound **3** (see Supporting Information File 1, Figures S1, S2 and S5, S6). Heating compound **5** with primary and secondary amines in dimethyl sulfoxide at 50–70 °C for 0.5 h gave the corresponding (2-substituted pyrimidin-4-yloxy)acetates **6**–**9**. In order to replace the 2-methylsulfonyl group with a hydroxy group, we tested several techniques. It was found that heating compound **5** with water in dimethyl sulfoxide at 100 °C or boiling for 1 h in water led to hydrolysis of both 2-methylsulfonyl and 4-(ethoxycarbonyl)methoxy groups with the formation of 6-methyluracil (**10**) in 40% and 56% yields, respectively. Compound **11** was successfully synthesized by stirring compound **5** with aqueous sodium hydroxide solution in dioxane at room temperature for 12 h. Hydroxamic acids **12**–**18** were synthesized by the interaction of esters **3**, **4**, and **6**–**9**, with hydroxylamine in water at 0–5 °C (in case of ester **7**, in a mixture of water and methanol 1:1 at room temperature).

**Scheme 2:**
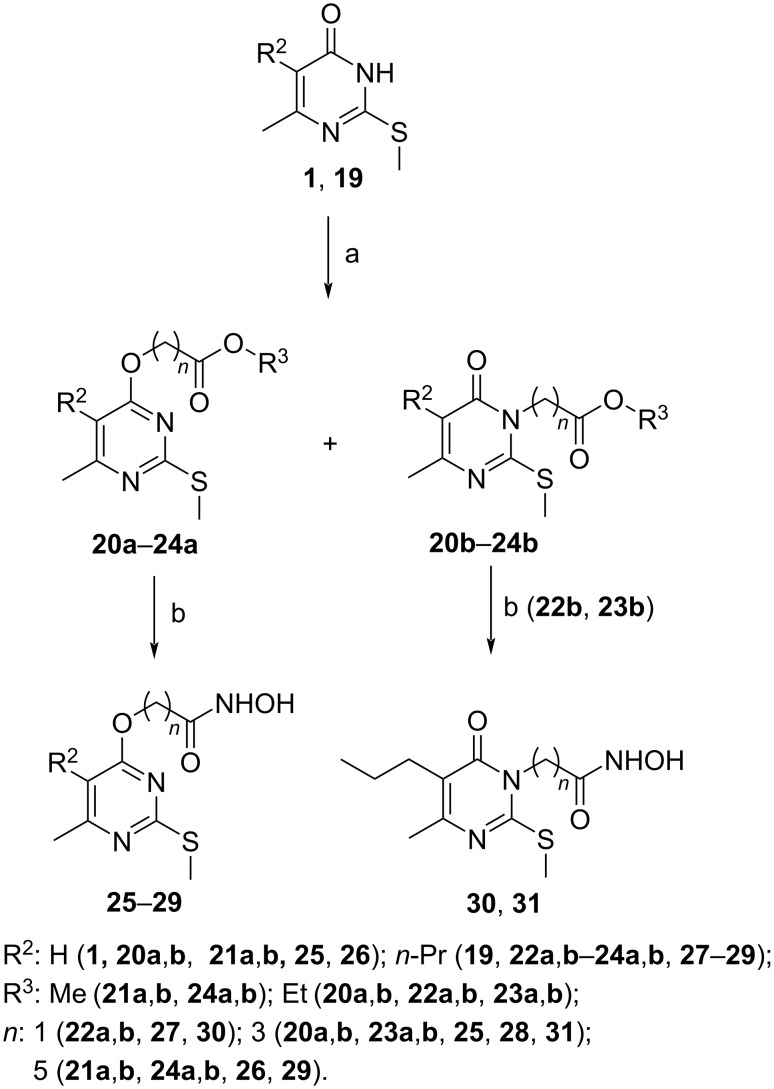
Synthesis of compounds **20**–**31**. Reagents and conditions: (a) ethyl 2-bromoethanoate (for **22**) (or ethyl 4-bromobutanoate (for **20**, **23**), or methyl 6-bromohexanoate (for **21**, **24**)), TBAB, TEA, 50–60 °C, 0.5–4 h; (b) NH_2_OH**^.^**HCl, KOH, MeOH, 0–5 °C, 1 h, then ester **20**–**24**, 0–5 °C,1 h, then conc. HCl to pH 5–6.

To investigate the effect of an alkyl substituent at the fifth position of the pyrimidine ring and the length of the methylene bridge linking the pyrimidine ring and the hydroxamic acid residue on the HDAC activity, hydroxamic acids **25**–**31** were synthesized (Scheme 2).

In contrast to the synthesis of esters **3** and **4**, alkylation of pyrimidinones **1** and **19** with the corresponding bromoesters in triethylamine in the presence of tetrabutylammonium bromide at 50–60 °C afforded mixtures of the *O*-alkylation **20a**–**24a** and *N*(3)-alkylation **20b**–**24b** products (Table 1, entries 1–5), which are easily separated by column chromatography. The overall yields are reasonable with the exception of the synthesis **24a** and **24b**. To improve the yield of the alkylation reaction of compound **19** with methyl 6-bromohexanoate and select the conditions favoring the formation of the *O*-isomer, a weak base, potassium carbonate, and a bipolar aprotic solvent, dimethylformamide, were used. The reaction was carried out at room temperature for 120 hours and a significant increase in the overall yield (from 52 to 71%) and selectivity (from 1.4:1 to 2.5:1) in favor of the *O*-isomer **24a** were observed (compare entries 5 and 6 in Table 1). The best alkylation results were obtained when the alkylation reaction was carried out at 50–60 °C. These reaction conditions proved to be the most successful with an overall reaction yield as high as 82% and a ratio of *O*- to *N*-isomers **24a** to **24b** as high as 3.3:1 (Table 1, entry 7).

**Table 1:** Alkylation of pyrimidin-4(3*H*)-ones **1** and **19** with bromoesters (1.1 equiv).

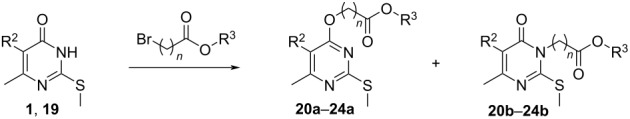								

entry	substr.	R^2^	R^3^	*n*	solvent (TBAB or base)	reaction temp., °C (time, h)	alkylation product (yield, %)	

							*O*-isomer	*N*-isomer

1	**1**	H	Et	3	TEA (TBAB)	50–60 (4)	**20a** (45)	**20b** (31)
2	**1**	H	Me	5	TEA (TBAB)	50–60 (4)	**21a** (50)	**21b** (28)
3	**19**	*n*-Pr	Et	1	TEA (TBAB)	50–60 (4)	**22a** (62)	**22b** (12)
4	**19**	*n*-Pr	Et	3	TEA (TBAB)	50–60 (4)	**23a** (37)	**23b** (32)
5	**19**	*n*-Pr	Me	5	TEA (TBAB)	50–60 (4)	**24a** (30)	**24b** (22)
6	**19**	*n*-Pr	Me	5	DMF (K_2_CO_3_)	rt (120)	**24a** (51)	**24b** (20)
7	**19**	*n*-Pr	Me	5	DMF (K_2_CO_3_)	50–60 (4)	**24a** (63)	**24b** (19)

As expected, the polar aprotic solvent dimethylformamide promoted the reaction of the electrophile with the more negatively charged oxygen atom by well solvating the cation. Thus, this method allows the synthesis of the *O*-isomer **24a** in an acceptable 63% yield, though the alkylation reaction is not very selective. Hydroxamic acids **25**–**31** were obtained in 52–92% yields in the usual way by treating the corresponding esters with hydroxylamine in methanol at 0–5 °C (Scheme 2).

**Figure 2:**
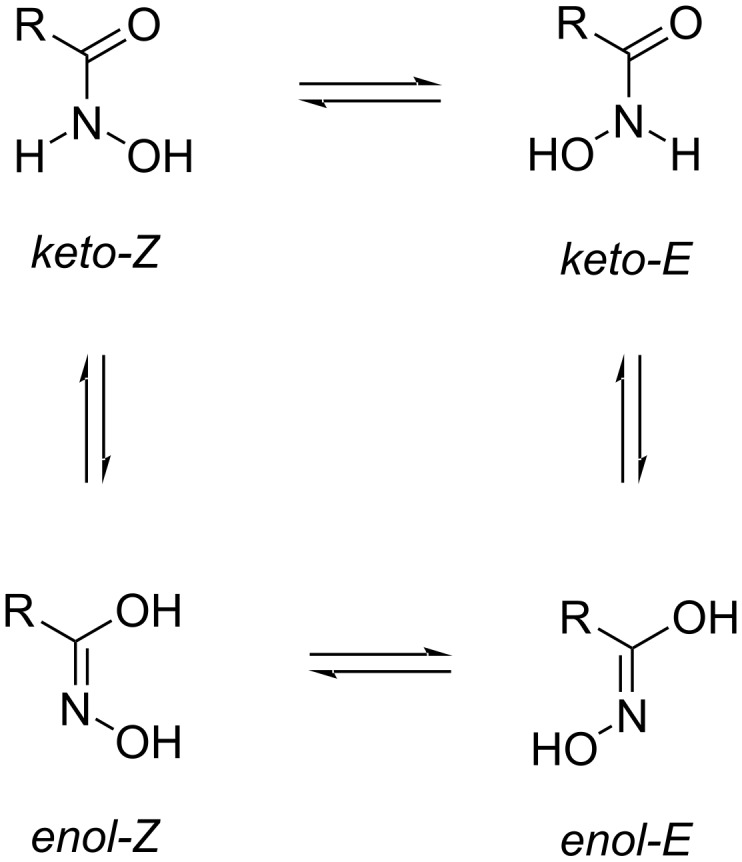
The conformational and tautomeric forms of hydroxamic acids according to [36].

It is known that there are two possible tautomeric forms of each hydroxamic acid: the *keto* and *enol* tautomer. Furthemore, each tautomer can adopt an *E* or *Z* conformation (Figure 2) [32–36].

**Figure 3:**
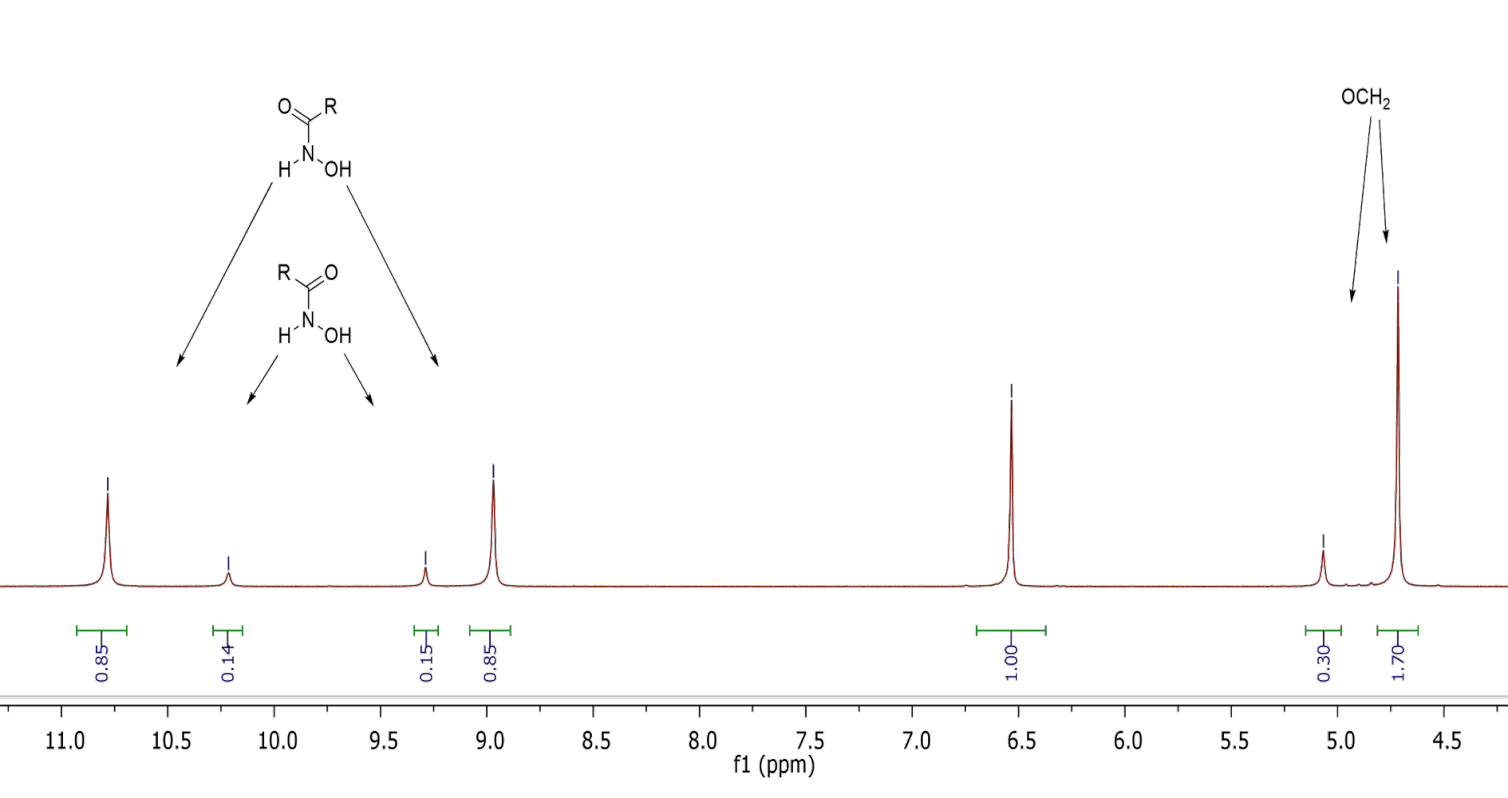
Fragment of the ^1^H NMR spectrum in DMSO-*d_6_* of compound **12**.

The results of NMR spectra and theoretical calculations showed that hydroxamic acids tend to adopt the more stable *keto-E* and *keto-Z* conformations [36]. In the ^1^H NMR spectra of the synthesized hydroxamic acids **12**–**18** and **25**–**31** two sets of proton signals from the NH and OH groups of the hydroxamic acid residue are observed. Following data [36], we assigned the more intense peaks to the *keto-E* form and the less intense ones to the *keto-Z* form. It is interesting to note that two proton signals of the OCH_2_ groups are also observed in the ^1^H NMR spectra of compounds **12**, **13**, **16–18**, **27**, and **30**. The fact that the ratio of the intensities of these signals is the same as the ratio of the intensities of the signals of the NH and OH groups of the hydroxamic acid moiety suggests that two OCH_2_ group signals are also due to the two isomeric forms. The fragment of the ^1^H NMR spectrum of compound **12** is presented in Figure 3 (full NMR spectra of the compounds are given in Supporting Information File 1).

### In vitro HDAC4 and HDAC8 inhibitory activities of the synthesized compounds

Compounds **12**–**18** and **25**–**31** were tested as inhibitors of HDAC4 and HDAC8 isoforms according to the procedures described previously [37]. The activity data of the tested compounds are presented in Table 2. A comparison of the IC_50_ values for compounds **12**–**18**, which differ only in the substituent at the second position of the pyrimidine ring, reveals that the presence of a methylthio group at this position gives compound **12** a slight advantage in the inhibition of the HDAC4 isoform, while the presence of a more bulky substituent at the second position of the pyrimidine ring favors the inhibition of HDAC8 isoforms (Table 2, compounds **13**–**15** and **17**). However, most of the compounds tested, with the exception of **29**, were inactive or showed weak inhibitory effect on the HDAC4 isoform.

**Table 2:** Inhibitory activities (IC_50_) of tested compounds against HDAC4 and HDAC8.

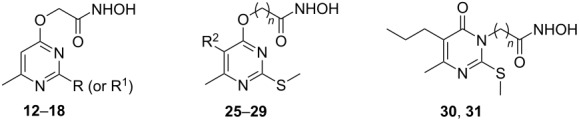						

compound	R	R^1^	R^2^	*n*	IC_50_^a^, μM	

					HDAC4	HDAC8

**12**	SMe				38	28
**13**	SEt				≥100	13
**14**		NHCH_2_CH=CH_2_			≥100	15
**15**		NHCH_2_C_6_H_5_			≥100	14
**16**		NMe_2_			≥100	35
**17**		pyrrolidin-1-yl			≥100	13
**18**		OH			≥100	60
**25**			H	3	>50	5.65
**26**			H	5	>50	2.05
**27**			*n*-Pr	1	38	1.4
**28**			*n*-Pr	3	>35	2.4
**29**			*n*-Pr	5	16.6	1.2
**30**				1	>35	>35
**31**				3	>35	>35
vorinostat					27^b^	5.3^b^

^a^IC_50_ values were determined by measuring the inhibition of enzymatic activity using BOC-LYS-(TFA)-AMC as a substrate, 100 mM TRIS, 300 mM KCl, pH 8.0 buffer, at 30 °C. ^b^Taken from ref [38].

Comparison of the potency of the HDAC8 isoform inhibitory activity of compounds **12**, **25**, and **26** with that of compounds **27**–**29** suggests that the propyl substituent at the position 5 of the pyrimidine ring is favorable for this effect. Extension of the methylene bridge connecting the pyrimidine ring to the hydroxamic acid residue to the same series of compounds also increases the ability of the compounds to inhibit the HDAC8 isoform, except in the case of compound **28**. It should be noted that both, HDAC4 and HDAC8 isoforms were most inhibited by compound **29** (IC_50_ 16.6 and 1.2 μM, respectively). The structure of this most active compound is distinguished by the fact that the compound not only has a propyl substituent at position 5 of the pyrimidine ring, but also has the longest pentamethylene group bridge connecting the pyrimidine ring to the hydroxamic acid residue.

## Conclusion

In summary, starting with corresponding 2-(alkylthio)pyrimidin-4(3*H*)-ones we have developed an efficient synthesis of hitherto unknown pyrimidine-based hydroxamic acids, wherein the hydroxamic acid residue is attached to the pyrimidine ring by a methylene linker of varying length. The ^1^H NMR spectra show that the synthesized hydroxamic acids in solution exist as an equilibrium mixture of two isomeric forms. The inhibitory activity of the synthesized hydroxamic acids on HDAC4 and HDAC8 isoforms shows that most of them are prone to inhibit HDAC8 isoform rather than HDAC4. The most potent inhibitor of both the HDAC4 and HDAC8 isoforms was found to be *N*-hydroxy-6-[6-methyl-2-(methylthio)-5-propylpyrimidin-4-yloxy]hexanamide (**29**) with an IC_50_ of 16.6 and 1.2 µM, respectively. We believe that the obtained data on HDAC inhibitory activity of the synthesized pyrimidine-based hydroxamic acids will be useful in the future design of potent HDAC inhibitors.

## Experimental

Melting points were determined in open capillaries with a digital melting point IA9100series apparatus (ThermoFisher Scientific). All reactions and purity of the synthesized compounds were monitored by TLC using silica gel 60 F254 aluminum plates (Merck). Visualization was accomplished by UV light. Column chromatography was performed using silica gel 60 (0.040–0.063 mm) (Merck). NMR spectra were recorded on a Bruker Ascend 400 spectrometer (400 MHz and 100 MHz for ^1^H and ^13^C, respectively). ^1^H NMR and ^13^C NMR were referenced to residual solvent peaks. High-resolution mass spectrometry (HRMS) analyses were carried out on a Dual-ESI Q-TOF 6520 (Agilent Technologies) mass spectrometer.

The starting compounds **1** [39], **2** [39] and **19** [40] were prepared following the reported methods. Synthetic details, characterization and analytical data as well as ^1^H and ^13^C NMR spectra of all synthesized compounds are presented in the Supporting Information File 1.

### General procedure for the synthesis of esters **3**, **4**, and **20–24**

To a mixture of pyrimidinone **1**, **2** or **19** (1 mmol) and TBAB (0.032 g, 0.1 mmol) in TEA (0.4 mL) at 50–60 °C the corresponding bromoester (1.1 mmol) was added dropwise under stirring. The reaction mixture was stirred at this temperature for 0.5–4 h (for details see Supporting Information File 1), then cooled to room temperature, and poured into ice water (30 mL). In case of esters **3** and **4**, the resultant precipitate was collected by filtration, washed with cold water, dried, and recrystallized from hexane. In case of esters **20–24**, the resultant mixture was extracted with chloroform (3 × 10 mL). The organic solutions were combined, washed with brine, dried over Na_2_SO_4_, and evaporated under reduced pressure. The residue was purified by column chromatography.

### General procedure for the synthesis of esters **6–9**

To a suspension of compound **5** (0.274 g, 1 mmol) in dry DMSO (0.3 mL) the corresponding amine (2 mmol; in the case of **8** – the solution of dimethylamine in DMSO) was added. The reaction mixture was stirred at 50–70 °C for 0.5 h, then cooled to room temperature, and poured into ice water (10 mL). The resultant precipitate was collected by filtration, washed with water, dried, and recrystallized from hexane.

### 6-Methyluracil (**10**)

Method A. To a suspension of compound **5** (0.274 g, 1 mmol) in DMSO (0.3 mL) H_2_O (0.036 g, 2 mmol) was added. The reaction mixture was stirred at 100 °C for 0.5 h, then cooled to room temperature, and poured into ice water (5 mL). The resultant precipitate was collected by filtration, washed with ice water, and dried to yield white powder.

Method B. A mixture of compound **5** (0.274 g, 1 mmol) and H_2_O (5 mL) was heated at reflux for 1 h, then cooled to 0 °C. The resultant precipitate was collected by filtration, washed with ice water, and dried to yield 0.07 g (56%) of compound **10**.

### Ethyl (6-methyl-2-oxo-1,2-dihydropyrimidin-4-yloxy]acetate (**11**)

To a solution of compound **5** (0.274 g, 1 mmol) in dioxane (25 mL) a 1 M aqueous solution of NaOH (2 mL) was added. The reaction mixture was stirred at room temperature for 12 h, then water (15 mL) was added to the mixture, and stirring was continued for 5 min. The reaction mixture was acidified with conc. HCl to pH 2 and extracted with DCM. The extract was washed with brine, dried over Na_2_SO_4_, and evaporated under reduced pressure. The residue was purified by column chromatography to give 0.155 g (73%) of compound **11**.

### General procedure for the synthesis of hydroxamic acids **12–18**

To a mixture of esters **3**, **4**, **6**–**9**, or **11** (1 mmol) and NH_2_OH·HCl (0.204 g, 3 mmol) in H_2_O (3 mL) (in the case of ester **7** in a mixture H_2_O/MeOH 1:1, 6 mL) at 0–5 °C a solution of KOH (0.392 g, 7 mmol) in H_2_O (1 mL) was added dropwise under stirring. The reaction mixture was stirred at this temperature for 1–4 h (in the case of ester **7** it was stirred at room temperature for 96 h), then acidified with conc. HCl to pH 5–6. The resultant precipitate was collected by filtration, washed with ice water, and dried to yield compounds **12**–**18**.

### General procedure for the synthesis of hydroxamic acids **25–31**

A mixture of NH_2_OH·HCl (0.556 g, 8 mmol), KOH (0.672 g, 12 mmol), and methanol (5 mL) was stirred at room temperature for 0.5 h, then cooled to 0–5 °C and filtered. To the filtrate the corresponding ester **20**–**24** (1 mmol) was added. The reaction mixture was stirred at 0–5 °C for 1 h, then the solvent was removed under reduced pressure, the residue was dissolved in water, cooled to 0–5 °C and acidified with conc. HCl to pH 5–6. The resultant precipitate was collected by filtration, washed with ice water and dried (compound **30** was recrystallized from a mixture of ethanol/water 1:1). In case of compound **29**, after acidification, the reaction mixture was extracted with chloroform (3 × 10 mL), the organic solutions were combined, washed with brine, dried over Na_2_SO_4_, and evaporated under reduced pressure. The residue was purified by column chromatography.

## Supporting Information

File 1 Compounds characterization and analytical data; HDAC enzyme activity assay; references; NMR spectra.
