# Supplementary material for: Synthesis and HDAC inhibitory activity of pyrimidine-based hydroxamic acids
Source: Beilstein J Org Chem. 2022 Jul 13;18:837–44. doi: 10.3762/bjoc.18.84 (PMC9296983; doi:10.3762/bjoc.18.84)
Supplement: File 1 — Compounds characterization and analytical data; HDAC enzyme activity assay; references; NMR spectra. [file Beilstein_J_Org_Chem-18-837-s001.pdf]

BEILSTEIN JOURNAL OF ORGANIC CHEMISTRY

Supporting Information

for

Synthesis and HDAC inhibitory activity of pyrimidine-based hydroxamic acids

Virginija Jakubkiene, Gabrielius Ernis Valiulis, Markus Schweipert, Asta Zubriene, Daumantas Matulis, Franz-Josef Meyer-Almes and Sigitas Tumkevicius

Beilstein J. Org. Chem. **2022,** *18,* 837–844. doi:10.3762/bjoc.18.84

Compounds characterization and analytical data; HDAC enzyme activity assay; references; NMR spectra

License and Terms: This is a supporting information file under the terms of the Creative Commons Attribution License (https://creativecommons.org/licenses/by/4.0). Please note that the reuse, redistribution and reproduction in particular requires that the author(s) and source are credited and that individual graphics may be subject to special legal provisions.
The license is subject to the *Beilstein Journal of Organic Chemistry* terms and conditions: (https://www.beilstein-journals.org/bjoc/terms)

Table of contents

Compounds characterization and analytical data S2

HDAC enzyme activity assay S7

References S7

Copies of NMR spectra S8

Compounds characterization and analytical data

*Ethyl [6-methyl-2-(methylthio)pyrimidin-4-yloxy]acetate (**3**).* White crystals; yield 0.17 g (73%); mp 52–53 °C; ^{1}H NMR ($CDCl_3$): δ 6.37 (s, 1H, CH), 4.87 (s, 2H, OCH_2), 4.23 (q, *J* = 7.2 Hz, 2H, CH_2), 2.47 (s, 3H, SCH_3), 2.38 (s, 3H, CH_3), 1.26 (t, *J* = 7.2 Hz, 3H, CH_3); ^{13}C NMR ($CDCl_3$): δ 171.4, 168.5 (2), 168.2, 102.1, 62.8, 61.4, 23.9, 14.3, 14.0; HRMS calcd for $C_{10}H_{14}N_2O_3S$: $[M + H]^+$ = 243.0798, found: 243.0797.

*Ethyl [2-(ethylthio)-6-methylpyrimidin-4-yloxy]acetate (**4**).* White crystals; yield 0.18 g (70%); mp 50–52 °C; ^{1}H NMR ($CDCl_3$): δ 6.36 (s, 1H, CH), 4.86 (s, 2H, OCH_2), 4.22 (q, *J* = 7.2 Hz, 2H, CH_2), 3.05 (q, *J* = 7.2 Hz, 2H, SCH_2), 2.37 (s, 3H, CH_3), 1.34 (t, *J* = 7.2 Hz, 3H, CH_3), 1.27 (t, *J* = 7.2 Hz, 3H, CH_3); ^{13}C NMR ($CDCl_3$): δ 171.0, 168.6, 168.5, 168.2, 102.1, 62.7, 61.5, 25.2, 23.9, 14.8, 14.3; HRMS calcd for $C_{11}H_{16}N_2O_3S$: $[M + H]^+$ = 257.0954, found: 257.0954.

*Ethyl [6-methyl-2-(methylsulfonyl)pyrimidin-4-yloxy]acetate (**5**)*
A mixture of compound **3** (0.242g, 1 mmol) and oxone (1.54 g, 2.5 mmol) in dry DMF (2 mL) was stirred at 40 °C for 0.5 h, then cooled to room temperature, and poured into ice water. The resultant precipitate collected by filtration, washed with water, and dried to give white solid, yield 0.214 g (78%); mp 83–85 °C; ^{1}H NMR ($CDCl_3$): δ 6.88 (s, 1H, CH), 4.98 (s, 2H, OCH_2), 4.24 (q, *J* = 7.2 Hz, 2H, CH_2), 3.27 (s, 3H, SO_2CH_3), 2.58 (s, 3H, CH_3), 1.28 (t, *J* = 7.2 Hz, 3H, CH_3); ^{13}C NMR ($CDCl_3$): δ 170.3, 169.7, 167.6, 164.6, 109.9, 63.6, 61.9, 39.1, 24.1, 14.3; HRMS calcd for $C_{10}H_{14}N_2O_5S$: $[M + H]^+$ = 275.0696, found: 275.0691.

*Ethyl [2-(allylamino)-6-methylpyrimidin-4-yloxy]acetate (**6**).* White crystals; yield 0.203 g (81%); mp 108–110 °C; ^{1}H NMR ($CDCl_3$): δ 6.01 (s, 1H, CH), 5.95–5.82 (m, 1H, CH=), 5.41 (very br. s, 1H, NH), 5.20 (d, *J* = 17.2 Hz, 1H, CH_2=), 5.09 (d, *J* = 10.0 Hz, 1H, CH_2=), 4.79 (s, 2H, OCH_2), 4.21 (q, *J* = 7.2 Hz, 2H, CH_2), 4.04–3.91 (m, 2H, NH$\underline{CH_2}$), 2.26 (s, 3H, CH_3), 1.26 (t, *J* = 7.2 Hz, 3H, CH_3); ^{13}C NMR ($CDCl_3$): δ 169.5, 168.9, 168.6, 161.6, 135.2, 115.8, 96.1, 62.5, 61.3, 43.9, 23.7, 14.3; HRMS calcd for $C_{12}H_{17}N_3O_3$: $[M + H]^+$ = 252.1343, found: 252.1343.

*Ethyl [2-(benzylamino)-6-methylpyrimidin-4-yloxy]acetate (**7**).* White crystals; yield 0.245 g (81%); mp 117–119 °C; ^{1}H NMR ($CDCl_3$): δ 7.36–7.20 (m, 5H, C_6H_5), 6.03 (s, 1H, CH), 5.55 (very br. s, 1H, NH), 4.76 (s, 2H, OCH_2), 4.55 (d, *J* = 6 Hz, 2H, NH$\underline{CH_2}$), 4.13 (q, *J* = 7 Hz, 2H, CH_2), 2.27 (s, 3H, CH_3), 1.21 (t, *J* = 7 Hz, 3H, CH_3); ^{13}C NMR ($CDCl_3$): δ 169.5, 168.9, 168.8, 161.7, 139.5, 128.7, 127.5, 127.3, 96.3, 62.4, 61.2, 45.5, 23.9, 14.3; HRMS calcd for $C_{16}H_{19}N_3O_3$: $[M + H]^+$ = 302.1499, found: 302.1499.

*Ethyl [2-(dimethylamino)-6-methylpyrimidin-4-yloxy]acetate (**8**).* White crystals; yield 0.168 g (70%); mp 58–60 °C; ^{1}H NMR ($CDCl_3$): δ 5.93 (s, 1H, CH), 4.77 (s, 2H, OCH_2), 4.21 (q, *J* = 7.2 Hz, 2H, CH_2), 3.10 (s, 6H, $N(CH_3)_2$), 2.27 (s, 3H, CH_3), 1.25 (t, *J* = 7.2 Hz, 3H, CH_3); ^{13}C NMR ($CDCl_3$): δ 169.3, 168.8, 168.6, 161.8, 94.2, 62.4, 61.1, 36.9, 24.3, 14.3; HRMS calcd for $C_{11}H_{17}N_3O_3$: $[M + H]^+$ = 240.1343, found: 240.1343.

*Ethyl [6-methyl-2-(pyrrolidin-1-yl)pyrimidin-4-yloxy]acetate (**9**).* White crystals; yield 0.241 g (91%); mp 85–87 °C; ^{1}H NMR ($CDCl_3$): δ 5.94 (s, 1H, CH), 4.79 (s, 2H, OCH_2), 4.21 (q, *J* = 7.2

Hz, 2H, CH_2), 3.50 (br. s, 4H, $N(CH_2)_2$), 2.28 (s, 3H, CH_3), 1.98–1.86 (m, 4H, $(CH_2)_2$), 1.25 (t, *J* = 7.2 Hz, 3H, CH_3); ^{13}C NMR ($CDCl_3$): δ 169.3, 168.8, 168.6, 159.9, 94.2, 62.4, 61.1, 46.6, 25.6, 24.3, 14.3; HRMS calcd for $C_{13}H_{19}N_3O_3$: $[M + H]^+$ = 266.1499, found: 266.1499.

*6-Methyluracil (**10**).* Method **A**. White powder; yield 0.05 g (40%); mp 320 °C (decomp.) (320 °C lit. [1]); ^{1}H NMR (DMSO-*d*$_6$): δ 10.87 (s, 1H, NH); 10.81 (s, 1H, NH); 5.31 (s, 1H, CH), 2.00 (s, 3H, CH_3); ^{13}C NMR (DMSO-*d*$_6$): δ 164.1, 152.9, 151.6, 98.7, 18.2.
Method **B**. White powder; yield 0.07 g (56%) , mp 320 °C (decomp).

*Ethyl (6-methyl-2-oxo-1,2-dihydropyrimidin-4-yloxy]acetate (**11**).* White powder; R_f 0.5 (chloroform : ethyl acetate : methanol – 4 : 1 : 1); yield 0.155 g (73%); mp 210–212 °C (197 °C lit.[2]); ^{1}H NMR ($CDCl_3$): δ 12.85 (br. s, 1H, NH), 5.89 (s, 1H, CH), 4.97 (s, 2H, OCH_2), 4.22 (q, J = 7.2 Hz, 2H, CH_2), 2.32 (s, 3H, CH_3), 1.27 (t, J = 7.2 Hz, 3H, CH_3); ^{13}C NMR ($CDCl_3$): δ 172.3, 167.9, 160.3, 157.6, 94.8, 62.6, 61.6, 18.9, 14.2; HRMS calcd for $C_9H_{12}N_2O_4$: $[M + H]^+$ = 213.0870, found: 213.0869.

*N-Hydroxy-(6-methyl-2-(methylthio)pyrimidin-4-yloxy]acetamide (**12**).* Reaction time 1 h; white solid; yield 0.186 g (81%); mp 182–184 °C; ^{1}H NMR (DMSO-*d*$_6$): δ 10.78 and 10.21 (2s, 1H, NH), 9.29 and 8.97 (2s, 1H, OH), 6.53 (s, 1H, CH), 5.07 and 4.72 (2s, 2H, OCH_2), 2.45 (s, 3H, SCH_3), 2.32 (s, 3H, CH_3) 1.28 (t, *J* = 8 Hz, 3H, CH_3);; ^{13}C NMR (DMSO-*d*$_6$): δ 170.2, 168.1 (2), 164.0, 102.1, 62.8, 23.3, 13.4; HRMS calcd for $C_8H_{11}N_3O_3S$: $[M + H]^+$ = 230.0594, found: 230.0594.

*[2-(Ethylthio)-6-methylpyrimidin-4-yloxy]-N-hydroxyacetamide (**13**).* Reaction time 1 h; white solid; yield 0.177 g (73%); mp 175–176 °C; ^{1}H NMR (DMSO-*d*$_6$): δ 10.76 and 10.21 (2s, 1H, NH), 9.25 and 8.94 (2s, 1H, OH), 6.53 (s, 1H, CH), 5.06 and 4.71 (2s, 2H, OCH_2), 3.04 (q, *J* = 8 Hz, 2H, CH_2), 2.31 (s, 3H, CH_3); ^{13}C NMR (DMSO-*d*$_6$): δ 169.8, 168.1 (2), 164.0, 102.2, 62.7, 24.4, 23.3, 14.7; HRMS calcd for $C_9H_{13}N_3O_3S$: $[M + H]^+$ = 244.0750, found: 244.0748.

*[2-(Allylamino)-6-methylpyrimidin-4-yloxy]-N-hydroxyacetamide (**14**).* Reaction time 4 h; white solid; yield 0.162 g (68%); mp 177–179 °C; ^{1}H NMR (DMSO-*d*$_6$): δ 10.71 and 10.12 (2s, 1H, NH), 8.94 (br. s, 1H, OH), 6.02–5.78 (m, 2H, CH, CH=), 5.15 (dd, *J* = 17.2 and 1.2 Hz, 1H, CH_2=), 5.02 (dd, *J* = 10.2 and 1.4 Hz, 1H, CH_2=), 4.94 and 4.62 (2s, 2H, OCH_2), 3.85 (br. s, 2H, NH$\underline{CH_2}$), 2.15 (s, 3H, CH_3); ^{13}C NMR (DMSO-*d*$_6$): δ 168.9, 168.0, 164.4, 161.6, 136.1, 115.0, 94.6, 62.1, 43.1, 23.5; HRMS calcd for $C_{10}H_{14}N_4O_3$: $[M + H]^+$ = 239.1139, found: 239.1140.

*[2-(Benzylamino)-6-methylpyrimidin-4-yloxy]-N-hydroxyacetamide (**15**).* Reaction time 96 h; white solid; yield 0.176 g (61%); mp 168–170 °C; ^{1}H NMR (DMSO-*d*$_6$): δ 10.71 and 10.15 (2s, 1H, NH), 9.20 and 8.91 (2s, 1H, OH), 7.64 (br. s, 1H, $\underline{NH}CH_2$), 7.37–7.16 (m, 5H, C_6H_5), 5.94 (s, 1H, CH), 5.02 – 4.33 (m, 4H, OCH_2, NH$\underline{CH_2}$), 2.15 (s, 3H, CH_3); ^{13}C NMR (DMSO-*d*$_6$): δ 168.9, 164.4, 161.7, 158.7, 140.6, 128.1, 127.5, 126.5, 94.9, 62.2, 44.0, 23.5; HRMS calcd for $C_{14}H_{16}N_4O_3$: $[M + H]^+$ = 289.1295, found: 289.1295.

*[2-(Dimethylamino)-6-methylpyrimidin-4-yloxy]-N-hydroxyacetamide (**16**).* Reaction time 1 h; white solid; yield 0.174 g (77%); mp 192–194 °C; ^{1}H NMR (DMSO-*d*$_6$): δ 10.73 and 10.11 (2s, 1H, NH), 9.22 and 8.89 (2s, 1H, OH), 5.93 (s, 1H, CH), 4.98 and 4.62 (2s, 2H, OCH_2), 3.05 (s, 6H, $(CH_3)_2$), 2.19 (s, 3H, CH_3); ^{13}C NMR (DMSO-*d*$_6$): δ 168.5, 167.8, 164.6, 161.3, 93.8, 62.1, 36.4, 23.8; HRMS calcd for $C_9H_{14}N_4O_3$: $[M + H]^+$ = 227.1139, found: 227.1136.

*N-Hydroxy-[6-methyl-2-(pyrrolidin-1-yl)pyrimidin-4-yloxy]acetamide (**17**)*. Reaction time 2 h; white solid; yield 0.176 g (70%); mp 178–180 °C; ^{1}H NMR (DMSO-d_6): δ 10.69 and 10.08 (2s, 1H, NH), 9.17 and 8.87 (2s, 1H, OH), 5.93 (s, 1H, CH), 4.99 and 4.61 (2s, 2H, OCH_2), 3.48–3.38 (m, 4H, $N(CH_2)_2$), 2.18 (s, 3H, CH_3), 1.92–1.83 (m, 4H, $(CH_2)_2$); ^{13}C NMR (DMSO-d_6): δ 168.5, 167.8, 164.6, 159.5, 93.8, 62.1, 46.1, 24.9, 23.7; HRMS calcd for $C_{11}H_{16}N_4O_3$: $[M + H]^+$ = 253.1295, found: 253.1297.

*N-Hydroxy-(2-hydroxy-6-methylpyrimidin-4-yloxy)acetamide (**18**)*. Reaction time 1 h; white solid; yield 0.109 g (55%); mp 190–191 °C (decomp.); ^{1}H NMR (DMSO-d_6): δ 11.39 (s, 1H, NH), 10.73 and 10.17 (2s, 1H, NH), 9.27 and 8.94 (2s, 1H, OH), 5.82 (s, 1H, CH), 4.97 and 4.61 (2s, 2H, OCH_2), 2.14 (s, 3H, CH_3); ^{13}C NMR (DMSO-d_6): δ 171.1, 163.9, 157.2, 156.4, 92.6, 62.3, 18.0; HRMS calcd for $C_7H_9N_3O_4$: $[M + H]^+$ = 200.0666, found: 200.0671.

*Ethyl 4-[6-methyl-2-(methylthio)pyrimidin-4-yloxy]butanoate (**20a**)*. Reaction time 4 h; isolated from a mixture of compounds **20a** and **20b**; R_f 0.37 (cyclohexane – ethyl acetate 4:1); colourless oil; yield 0.111 g (41%); ^{1}H NMR ($CDCl_3$): δ 6.18 (s, 1H, CH), 4.37 (t, *J* = 6.4 Hz, 2H, $O\underline{CH_2}CH_2$), 4.12 (q, *J* = 7.2 Hz, 2H, $O\underline{CH_2}CH_3$), 2.50 (s, 3H, SCH_3), 2.43 (t, *J* = 7.4 Hz, 2H, CH_2CO), 2.33 (s, 3H, CH_3), 2.13–2.00 (m, 2H, $CH_2\underline{CH_2}CH_2$), 1.23 (t, *J* = 7.2 Hz, 3H, $OCH_2\underline{CH_3}$); ^{13}C NMR ($CDCl_3$): δ 173.1, 171.4, 169.2, 167.7, 102.1, 65.4, 60.6, 30.9, 24.3, 23.7, 14.3, 14.1; HRMS calcd for $C_{12}H_{18}N_2O_3S$: $[M + H]^+$ = 271.1111, found: 271.1108.

*Ethyl 4-[6-methyl-2-(methylthio)-4-oxopyrimidin-3(4H)-yl]butanoate (**20b**)*. Reaction time 4 h; isolated from a mixture of compounds **20a** and **20b**; R_f 0.17 (cyclohexane – ethylacetate 4:1); colourless oil; yield 0.084 g (31%); ^{1}H NMR ($CDCl_3$): δ 5.97 (s, 1H, CH), 4.09 (q, *J* = 7.2 Hz, 2H, $O\underline{CH_2}CH_3$), 4.03 (t, *J* = 7.6 Hz, 2H, NCH_2), 2.51 (s, 3H, SCH_3), 2.36 (t, *J* = 7.4 Hz, 2H, CH_2CO), 2.16 (s, 3H, CH_3), 2.00 (quintet, *J* = 7.4 Hz, 2H, $CH_2\underline{CH_2}CH_2$), 1.21 (t, *J* = 7.2 Hz, 3H, $OCH_2\underline{CH_3}$); ^{13}C NMR ($CDCl_3$): δ 172.6, 162.3 (2), 161.4, 107.7, 60.6, 43.3, 31.5, 23.6, 22.8, 15.0, 14.2; HRMS calcd for $C_{12}H_{18}N_2O_3S$: $[M + H]^+$ = 271.1111, found: 271.1117.

*Methyl 6-[6-methyl-2-(methylthio)pyrimidin-4-yloxy]hexanoate (**21a**)*. Reaction time 2.75 h; isolated from a mixture of compounds **21a** and **21b**; R_f 0.7 (chloroform – ethyl acetate 4:1); white solid; yield 0.054 g (19%); mp 50 °C; ^{1}H NMR ($CDCl_3$): δ 6.19 (s, 1H, CH), 4.33 (t, *J* = 6.6 Hz, 2H, $O\underline{CH_2}CH_2$), 3.66 (s, 3H, OCH_3), 2.52 (s, 3H, SCH_3), 2.38–2.28 (m, 5H, CH_2CO, CH_3), 1.80–1.63 (m, 4H, $2CH_2$), 1.50–1.40 (m, 2H, CH_2); ^{13}C NMR ($CDCl_3$): δ 174.1, 171.4, 169.4, 167.7, 102.1, 66.2, 51.6, 34.1, 28.6, 25.7, 24.8, 23.8, 14.1; HRMS calcd for $C_{13}H_{20}N_2O_3S$: $[M + H]^+$ = 285.1267, found: 285.1268.

*Methyl 6-[6-methyl-2-(methylthio)-4-oxopyrimidin-3(4H)-yl]hexanoate (**21b**)*. Reaction time 2.75 h; isolated from a mixture of compounds **21a** and **21b**; R_f 0.31 (chloroform – ethyl acetate 4:1); white solid; yield 0.026 g (9%); mp 52–54 °C; ^{1}H NMR ($CDCl_3$): δ 6.01 (s, 1H, CH), 3.98 (t, *J* = 8 Hz, 2H, NCH_2), 3.66 (s, 3H, OCH_3), 2.54 (s, 3H, SCH_3), 2.32 (t, *J* = 7.6 Hz, 2H, CH_2CO), 2.20 (s, 3H, CH_3), 1.78–1.63 (m, 4H, $2CH_2$), 1.45–1.35 (m, 2H, CH_2); ^{13}C NMR ($CDCl_3$): δ 174.1, 162.4, 162.3, 161.3, 107.9, 51.6, 44.0, 34.0, 27.2, 26.5, 24.6, 23.8, 15.1; HRMS calcd for $C_{13}H_{20}N_2O_3S$: $[M + H]^+$ = 285.1267, found: 285.1274.

*Ethyl [6-methyl-2-(methylthio)-5-propylpyrimidin-4-yloxy]acetate (**22a**).* Reaction time 0.5 h; isolated from a mixture of compounds **22a** and **22b**; R_f 0.53 (cyclohexane – ethyl acetate 4:1); white solid; yield 0.1 g (35%); mp 52–54 °C; ^{1}H NMR ($CDCl_3$): δ 4.88 (s, 2H, OCH_2), 4.21 (q, *J* = 7.2 Hz, 2H, $O\underline{CH_2}CH_3$), 2.54 (t, *J* = 7.6 Hz, 2H, CH_2), 2.45 (s, 3H, SCH_3), 2.39 (s, 3H, CH_3), 1.55 (sextet, *J* = 7.6 Hz, 2H, CH_2), 1.25 (t, *J* = 7.2 Hz, 3H, $OCH_2\underline{CH_3}$), 0.96 (t, *J* = 7.6 Hz, 3H, CH_3); ^{13}C NMR ($CDCl_3$): δ 168.7, 167.2, 166.2, 165.9, 114.8, 62.9, 61.3, 26.9, 22.0, 21.4, 14.3, 14.2, 14.0; HRMS calcd for $C_{13}H_{20}N_2O_3S$: $[M + H]^+$ = 285.1267, found: 285.1270.

*Ethyl [6-methyl-2-(methylthio)-4-oxo-5-propylpyrimidin-3(4H)-yl]acetate (**22b**).* Reaction time 0.5 h; isolated from a mixture of compounds **22a** and **22b**; R_f 0.39 (cyclohexane – ethyl acetate 4:1); white solid; yield 0.029 g (10%); mp 87–88 °C; ^{1}H NMR ($CDCl_3$): δ 4.77 (s, 2H, NCH_2), 4.23 (q, *J* = 7.2 Hz, 2H, $O\underline{CH_2}CH_3$), 2.55 (s, 3H, SCH_3), 2.45 (t, *J* = 7.6 Hz, 2H, CH_2), 2.27 (s, 3H, CH_3), 1.49 (sextet, *J* = 7.6 Hz, 2H, CH_2), 1.27 (t, *J* = 7.2 Hz, 3H, $OCH_2\underline{CH_3}$), 0.94 (t, *J* = 7.6 Hz, 3H, CH_3); ^{13}C NMR ($CDCl_3$): δ 167.0, 162.3, 158.3, 157.3, 119.6, 62.0, 45.2, 28.2, 21.7, 21.6, 14.9, 14.3, 14.2; HRMS calcd for $C_{13}H_{20}N_2O_3S$: $[M + H]^+$ = 285.1267, found: 285.1267.

*Ethyl 4-[6-methyl-2-(methylthio)-5-propylpyrimidin-4-yloxy]butanoate (**23a**).* Reaction time 4 h; isolated from a mixture of compounds **23a** and **23b**; R_f 0.69 (chloroform – ethyl acetate 30:1); colourless oil; yield 0.116 g (37%); ^{1}H NMR ($CDCl_3$): δ 4.37 (t, *J* = 6.4 Hz, 2H, OCH_2), 4.12 (q, *J* = 7.2 Hz, 2H, $O\underline{CH_2}CH_3$), 2.49 (s, 3H, SCH_3), 2.48–2.41 (m, 4H, $2CH_2$), 2.35 (s, 3H, CH_3), 2.08 (quintet, *J* = 6.8 Hz, 2H, $CH_2\underline{CH_2}CH_2$), 1.45 (sextet, *J* = 7.6 Hz, 2H, CH_2), 1.24 (t, *J* = 7.2 Hz, 3H, $OCH_2\underline{CH_3}$), 0.92 (t, *J* = 7.4 Hz, 3H, CH_3); ^{13}C NMR ($CDCl_3$): δ 173.1, 167.3, 167.0, 165.0, 114.7, 65.4, 60.6, 31.0, 26.8, 24.4, 22.1, 21.3, 14.3, 14.1 (2); HRMS calcd for $C_{15}H_{24}N_2O_3S$: $[M + H]^+$ = 313.1580, found: 313.1580.

*Ethyl 4-[6-methyl-2-(methylthio)-4-oxo-5-propylpyrimidin-3(4H)-yl]butanoate (**23b**).* Reaction time 4 h; isolated from a mixture of compounds **23a** and **23b**; R_f 0.5 (chloroform – ethyl acetate 30:1); white solid; yield 0.1 g (32%); mp 53–55 °C; ^{1}H NMR ($CDCl_3$): δ 4.13 (q, *J* = 7.2 Hz, 2H, $O\underline{CH_2}CH_3$), 4.06 (t, *J* = 7.6 Hz, 2H, NCH_2), 2.53 (s, 3H, SCH_3), 2.46–2.35 (m, 4H, $2CH_2$), 2.24 (s, 3H, CH_3), 2.04 (quintet, *J* = 7.6 Hz, 2H, $CH_2\underline{CH_2}CH_2$), 1.48 (sextet, *J* = 7.6 Hz, 2H, CH_2), 1.25 (t, *J* = 7.2 Hz, 3H, $OCH_2\underline{CH_3}$), 0.95 (t, *J* = 7.4 Hz, 3H, CH_3); ^{13}C NMR ($CDCl_3$): δ 172.8, 162.6, 157.8, 157.4, 119.7, 60.7, 43.9, 31.8, 28.3, 23.1, 21.7, 21.5, 14.8, 14.4, 14.3; HRMS calcd for $C_{15}H_{24}N_2O_3S$: $[M + H]^+$ = 313.1580, found: 313.1580.

*Methyl 6-[6-methyl-2-(methylthio)-5-propylpyrimidin-4-yloxy]hexanoate (**24a**).* Reaction time 5 h; isolated from a mixture of compounds **24a** and **24b**; R_f 0.47 (chloroform – ethyl acetate 30:1); colourless oil; yield 0.099 g (30%); ^{1}H NMR ($CDCl_3$): δ 4.37 (t, *J* = 6.4 Hz, 2H, OCH_2), 3.66 (s, 3H, OCH_3), 2.51 (s, 3H, SCH_3), 2.46 (t, *J* = 7.8 Hz, 3H, CH_2CO), 2.36 (s, 3H, CH_3), 2.34 (t, *J* = 7.6 Hz, 2H, CH_2), 1.82–1.65 (m, 4H, $2CH_2$), 1.53–1.41 (m, 4H, $2CH_2$), 0.93 (t, *J* = 7.4 Hz, 3H, CH_3); ^{13}C NMR ($CDCl_3$): δ 174.1, 167.3, 167.2, 164.8, 114.8, 66.2, 51.6, 34.1, 28.7, 26.9, 25.8, 24.8, 22.1, 21.3, 14.2, 14.1; HRMS calcd for $C_{16}H_{26}N_2O_3S$: $[M + H]^+$ = 327.1737, found: 327.1737.

*Methyl 6-[6-methyl-2-(methylthio)-4-oxo-5-propylpyrimidin-3(4H)-yl]hexanoate (**24b**).* Reaction time 5 h; isolated from a mixture of compounds **24a** and **24b**; R_f 0.3 (chloroform – ethyl acetate 30:1); white solid; yield 0.072 g (22%); mp 61–62 °C; ^{1}H NMR ($CDCl_3$): δ 3.98 (t, *J* = 7.8 Hz, 2H, NCH_2), 3.66 (s, 3H, OCH_3), 2.52 (s, 3H, SCH_3), 2.43 (t, *J* = 7.8 Hz, 3H, CH_2CO), 2.32 (t, *J* = 7.4 Hz, 2H, CH_2), 2.24 (s, 3H, CH_3), 1.79–1.60 (m, 4H, 2CH_2), 1.55–1.35 (m, 4H, 2CH_2), 0.95 (t, *J* = 7.2 Hz, 3H, CH_3); ^{13}C NMR ($CDCl_3$): δ 174.1, 162.6, 157.7, 157.3, 119.7, 51.6, 44.6, 34.0, 28.3, 27.3, 26.6, 24.6, 21.7, 21.5, 14.8, 14.3; HRMS calcd for $C_{16}H_{26}N_2O_3S$: $[M + H]^+$ = 327.1737, found: 327.1737.

*N-Hydroxy-4-[6-methyl-2-(methylthio)pyrimidin-4-yloxy]butanamide (**25**).* White solid; yield 0.134 g (52%); mp 150–151 °C. ^{1}H NMR (DMSO-d_6): δ 10.46 and 9.84 (2s, 1H, NH), 9.12 and 8.72 (2s, 1H, OH), 6.46 (s, 1H, CH), 4.27 (t, *J* = 6.4 Hz, 2H, OCH_2), 2.47 (s, 3H, SCH_3), 2.30 (s, 3H, CH_3), 2.08 (t, *J* = 7.4 Hz, 2H, CH_2CO), 1.91 (quintet, *J* = 6.8 Hz, 2H, $CH_2\underline{CH_2}CH_2$); ^{13}C NMR (DMSO-d_6): δ 170.3, 168.7, 168.4, 167.8, 101.8, 65.6, 28.7, 24.4, 23.3, 13.4; HRMS calcd for $C_{10}H_{15}N_3O_3S$: $[M + H]^+$ = 258.0907, found: 258.0906.

*N-Hydroxy-6-[6-methyl-2-(methylthio)pyrimidin-4-yloxy]hexanamide (**26**).* White solid; yield 0.193 g (68%); mp 112–113 °C. ^{1}H NMR (DMSO-d_6): δ 10.35 and 9.75 (2s, 1H, NH), 9.01 and 8.68 (2s, 1H, OH), 6.45 (s, 1H, CH), 4.27 (t, *J* = 6.4 Hz, 2H, OCH_2), 2.47 (s, 3H, SCH_3), 2.29 (s, 3H, CH_3), 1.95 (t, *J* = 7.2 Hz, 2H, CH_2CO), 1.68 (quintet, *J* = 7 Hz, 2H, CH_2); 1.53 (quintet, *J* = 7.2 Hz, 2H, CH_2), 1.40–1.27 (m, 2H, CH_2); ^{13}C NMR (DMSO-d_6): δ 170.3, 169.0, 168.8, 167.8, 101.8, 66.0, 32.2, 27.9, 25.0, 24.8, 23.3, 13.4; HRMS calcd for $C_{12}H_{19}N_3O_3S$: $[M + H]^+$ = 286.1220, found: 286.1223.

*N-Hydroxy-[6-methyl-2-(methylthio)-5-propylpyrimidin-4-yloxy]acetamide (**27**).* White solid; yield 0.230 g (85%); mp 144–146 °C. ^{1}H NMR (DMSO-d_6): δ 10.76 and 10.18 (2s, 1H, NH), 9.31 and 8.96 (2s, 1H, OH), 5.10 and 4.73 (2s, 2H, OCH_2), 2.55–2.46 (m, 2H, CH_2, overlaps with the residual signal of DMSO-d_6), 2.44 (s, 3H, SCH_3), 2.34 (s, 3H, CH_3), 1.48 (sextet, *J* = 7.4 Hz, 2H, CH_2), 0.90 (t, *J* = 7.4 Hz, 3H, CH_3); ^{13}C NMR (DMSO-d_6): δ 166.3, 166.0, 165.3, 164.3, 114.2, 62.9, 26.1, 21.4, 21.0, 13.9, 13.4; HRMS calcd for $C_{11}H_{17}N_3O_3S$: $[M + H]^+$ = 272.1063, found: 272.1069.

*N-Hydroxy-4-[6-methyl-2-(methylthio)-5-propylpyrimidin-4-yloxy]butanamide (**28**).* White solid; yield 0.275 g (92%); mp 125–127 °C. ^{1}H NMR (DMSO-d_6): δ 10.42 and 9.84 (2s, 1H, NH), 9.04 and 8.70 (2s, 1H, OH), 4.29 (t, *J* = 6.2 Hz, 2H, OCH_2), 2.48–2.42 (m, 5H, CH_2, SCH_3), 2.33 (s, 3H, CH_3), 2.10 (t, *J* = 7.2 Hz, 2H, CH_2), 1.92 (quintet, *J* = 6.8 Hz, 2H, CH_2), 1.45 (sextet, *J* = 7.4 Hz, 2H, CH_2), 0.89 (t, *J* = 7.4 Hz, 3H, CH_3); ^{13}C NMR (DMSO-d_6): δ 168.5, 166.5, 166.4, 164.8, 114.2, 65.5, 28.7, 26.1, 24.5, 21.5, 21.0, 13.8, 13.4; HRMS calcd for $C_{13}H_{21}N_3O_3S$: $[M + H]^+$ = 300.1376, found: 300.1376.

*N-Hydroxy-6-[6-methyl-2-(methylthio)-5-propylpyrimidin-4-yloxy]hexanamide (**29**).* R_f 0.32 (chloroform – ethyl acetate – methanol 14:1:1); yiellowish orange oil; yield 0.249 g (76%); ^{1}H NMR (DMSO-d_6): δ 10.34 and 9.74 (2 br. s, 1H, NH), 8.69 (very br. s, 1H, OH), 4.29 (t, *J* = 6.4 Hz, 2H, OCH_2), 2.48–2.41 (m, 5H, CH_2, SCH_3), 2.32 (s, 3H, CH_3), 1.95 (t, *J* = 7.4 Hz, 2H, CH_2), 1.69 (quintet, *J* = 7.1 Hz, 2H, CH_2), 1.54 (quintet, *J* = 7.5 Hz, 2H, CH_2), 1.44 (sextet, *J* = 7.5 Hz, 2H, CH_2), 1.36 (quintet, *J* = 7.2 Hz, 2H, CH_2), 0.88 (t, *J* = 7.4 Hz, 3H, CH_3); ^{13}C NMR (DMSO-d_6): δ 169.0, 166.6, 166.4, 164.7, 114.1, 66.0, 32.2, 28.0, 26.1, 25.1, 24.8, 21.5, 21.0, 13.8, 13.4; HRMS calcd for $C_{15}H_{25}N_3O_3S$: $[M + H]^+$ = 328.1689, found: 328.1689.

N-Hydroxy-[6-methyl-2-(methylthio)-4-oxo-5-propylpyrimidin-3(4H)-yl]acetamide (***30***). White solid; yield 0.141 g (58%); mp 185–187 °C (ethanol-water); ^{1}H NMR (DMSO-d_6): δ 10.78 and 10.39 (2s, 1H, NH), 9.46 and 9.01 (2s, 1H, OH), 4.82 and 4.50 (2s, 2H, NCH_2), 2.50 (s, 3H, SCH_3, overlaps with the residual signal of DMSO-d_6), 2.35 (t, J = 7.2 Hz, 2H, CH_2), 2.24 (s, 3H, CH_3), 1.40 (sextet, J = 7.2 Hz, 2H, CH_2), 0.88 (t, J = 7.2 Hz, 3H, CH_3); ^{13}C NMR (DMSO-d_6): δ 162.7, 161.3, 158.0, 157.2, 118.2, 43.8, 27.5, 21.3, 21.1, 14.4, 13.9; HRMS calcd for $C_{11}H_{17}N_3O_3S$: $[M + H]^+$ = 272.1063, found: 272.1066.

N-Hydroxy-4-[6-methyl-2-(methylthio)-4-oxo-5-propylpyrimidin-3(4H)-yl]butanamide (***31***). Yield 0.188 g (63%); mp 133–135 °C. ^{1}H NMR (DMSO-d_6): δ): δ 10.41 and 9.83 (2s, 1H, NH), 9.04 and 8.72 (2s, 1H, OH), 3.93 (t, J = 7.6 Hz, 2H, NCH_2), 2.51 (s, 3H, SCH_3, overlaps with the residual signal of DMSO-d_6), 2.35 (t, J = 7.6 Hz, 2H, CH_2), 2.21 (s, 3H, CH_3), 2.01 (t, J = 7.6 Hz, 2H, CH_2),), 1.81 (quintet, J = 7.6 Hz, 2H, CH_2), 1.40 (sextet, J = 7.4 Hz, 2H, CH_2), 0.88 (t, J = 7.4 Hz, 3H, CH_3); ^{13}C NMR (DMSO-d_6): δ 168.1, 161.3, 157.3, 157.0, 118.4, 43.6, 29.8, 27.5, 23.5, 21.2, 21.1, 14.2, 13.9; HRMS calcd for $C_{13}H_{21}N_3O_3S$: $[M + H]^+$ = 300.1376, found: 300.1375.

HDAC enzyme activity assay

Recombinant HDAC4 and 8 were produced as described in [3]. Serial dilutions of the inhibitor in the assay buffer (25 mM Tris-HCl, pH 8.0, 75 mM KCl, 0.001% Pluronic F-127) were incubated with HDAC in a black 96-well microtiter half-area plate (Greiner) for 60 min at 30 °C. Subsequently the reaction was started by the addition of 20 µM Boc-Lys(trifluoroacetyl)-AMC (Bachem) as a substrate. After incubation for 60 min at 30 °C, the reaction was stopped by the addition of 1.7 µM SATFMK. The deacetylated substrate was transformed into a fluorescent product by the addition of 0.4 mg/mL trypsin (Applichem). The release of AMC was followed in a microplate reader (PheraStar Plus, BMG Labtech) at 450 nm (λ_{Ex} = 350 nm) and correlated to enzyme activity. Dose–response curves were produced with GraphPad Prism and fitted to a four parameters logistic function to obtain IC_{50} values [4].

$$EA = E_0 + \frac{(E_{max} - E_0)}{1 + 10^{(\log(IC_{50}) - x) * h}}$$

in which EA is the enzyme activity at a given inhibitor concentration x; E_{max} and E_0 are the enzyme activities determined at zero and complete inhibition, respectively; IC_{50} represents the inhibitor concentration at which half the enzyme molecules are inhibited; h is the slope of the curve.

References

1. Gut, J.; Morávek, J.; Párkányi, C.; Prystaš, M.; Škoda, J.; Šorm, F. *Collect. Czech. Chem. Commun.* **1959**, *24*, 3154–3162. doi: 10.1135/cccc19593154
2. Bakavoli, M.; Eshghi, H.; Shiri, A.; Afrough, T.; Tajabadi, J. *Tetrahedron* **2013,** *69*, 8470–8476. doi:10.1016/j.tet.2013.07.025
3. Jänsch, N.; Meyners, C.; Muth, M.; Kopranovic, A.; Witt, O.; Oehme, I.; Meyer-Almes, F.-J. *Redox Biol.* **2019**, *20*, 60–67. doi: 10.1016/j.redox.2018.09.013
4. Volund, A. *Biometrics*. **1978**, *34*, 357. doi: 10.2307/2530598

Copies of NMR spectra

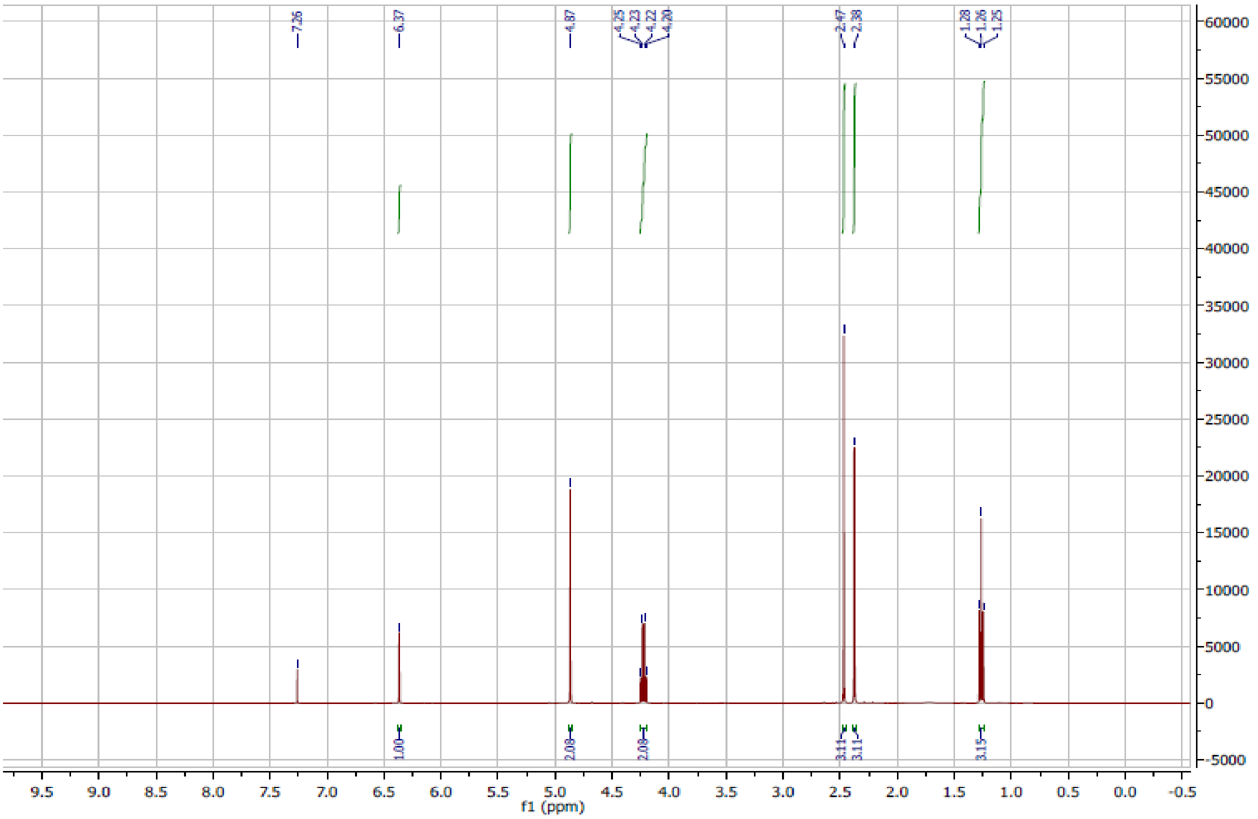

Figure S1: ^{1}H NMR spectrum of compound **3** ($CDCl_3$).

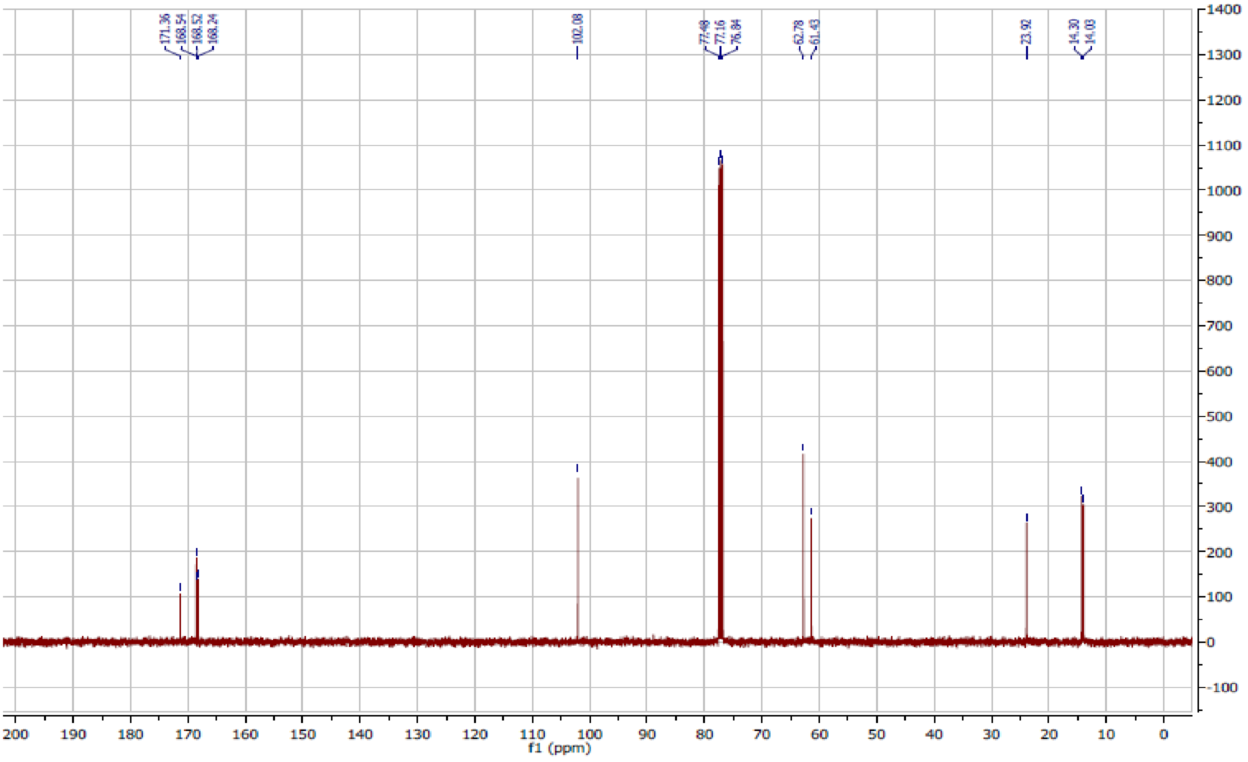

Figure S2: ^{13}C NMR spectrum of compound **3** ($CDCl_3$).

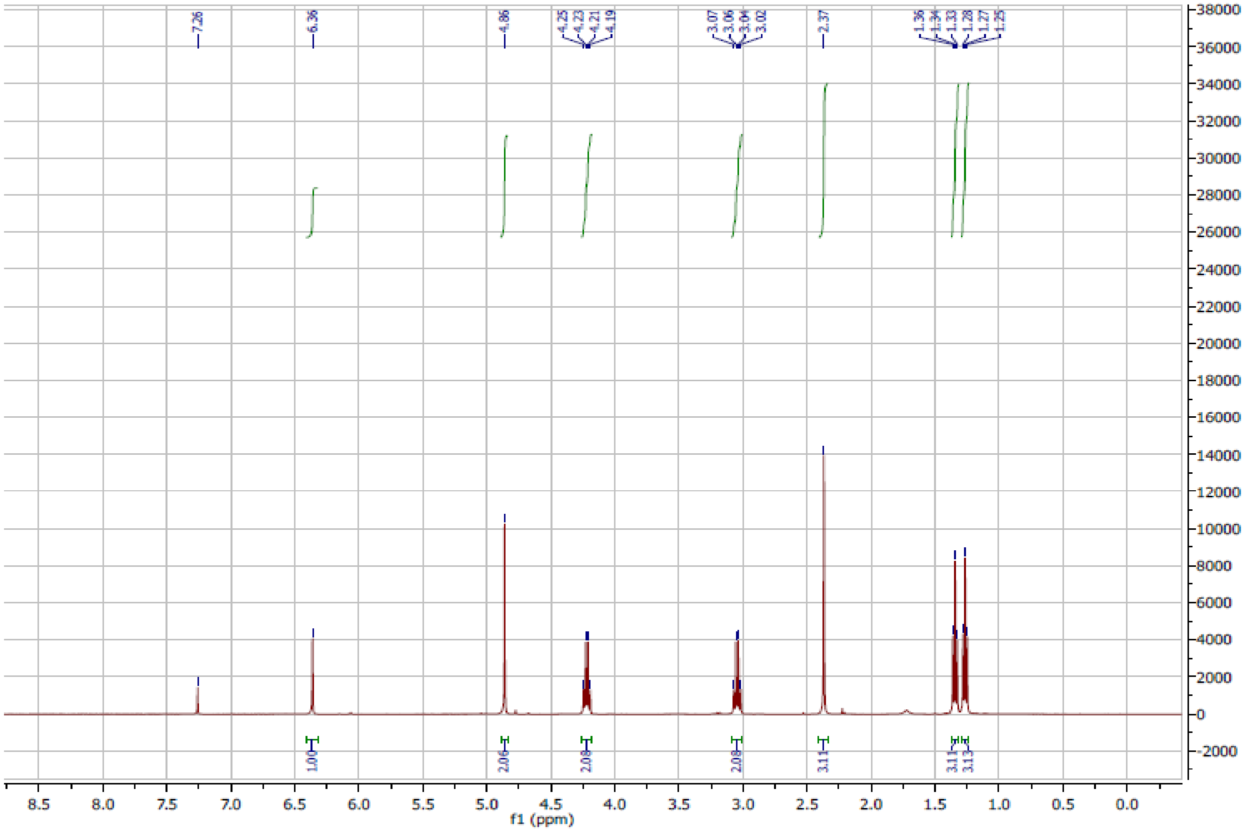

Figure S3: ^{1}H NMR spectrum of compound **4** ($CDCl_3$).

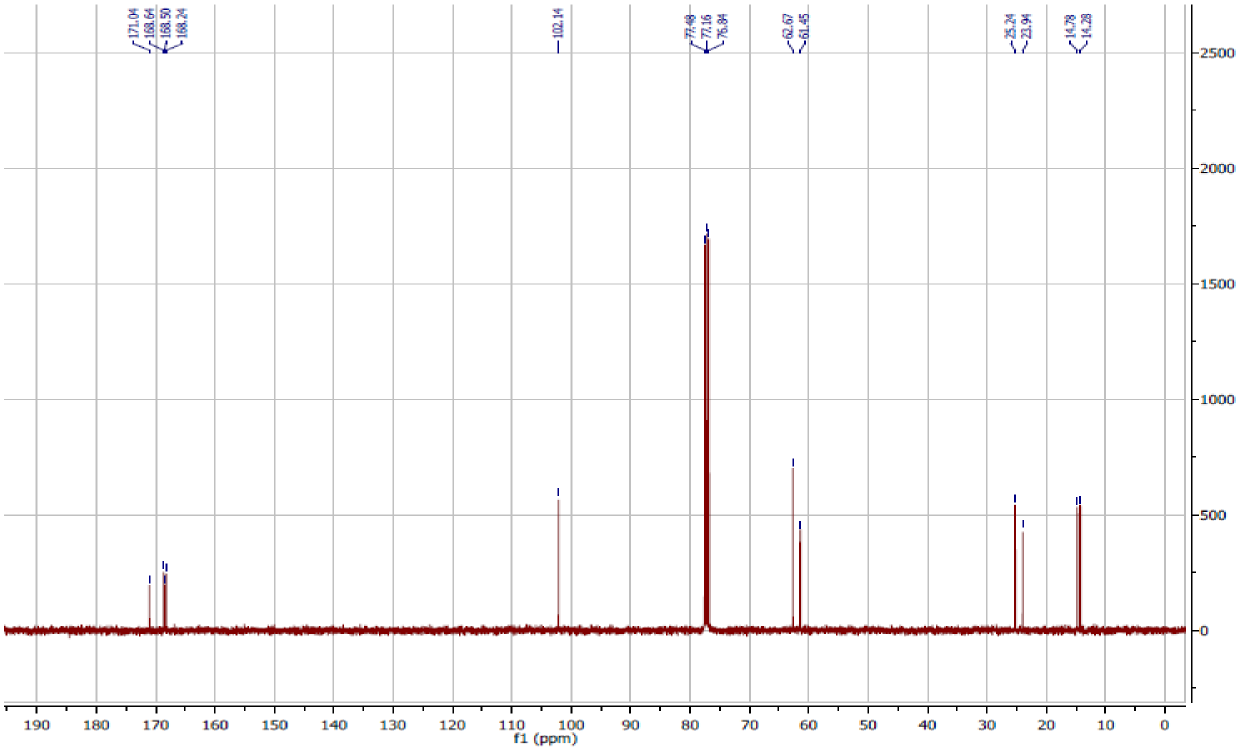

Figure S4: ^{13}C NMR spectrum of compound **4** ($CDCl_3$).

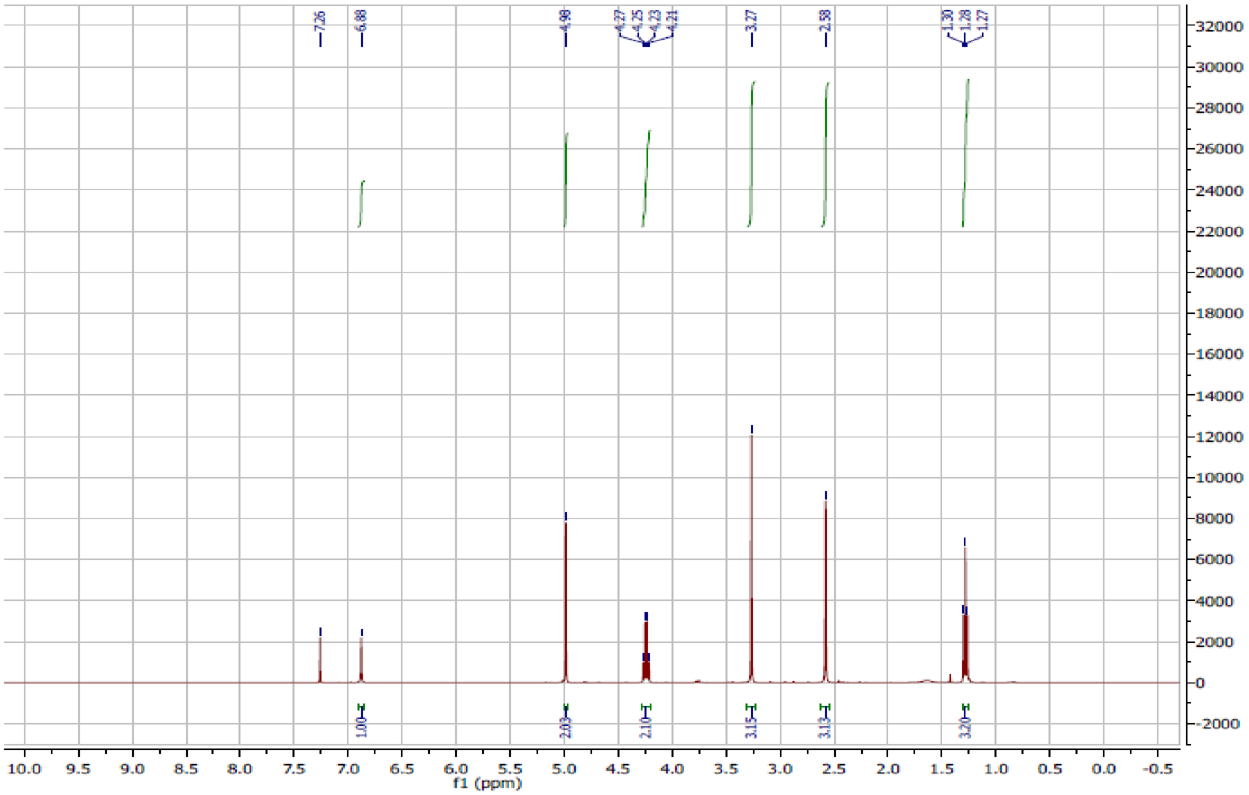

Figure S5: ^{1}H NMR spectrum of compound **5** ($CDCl_3$).

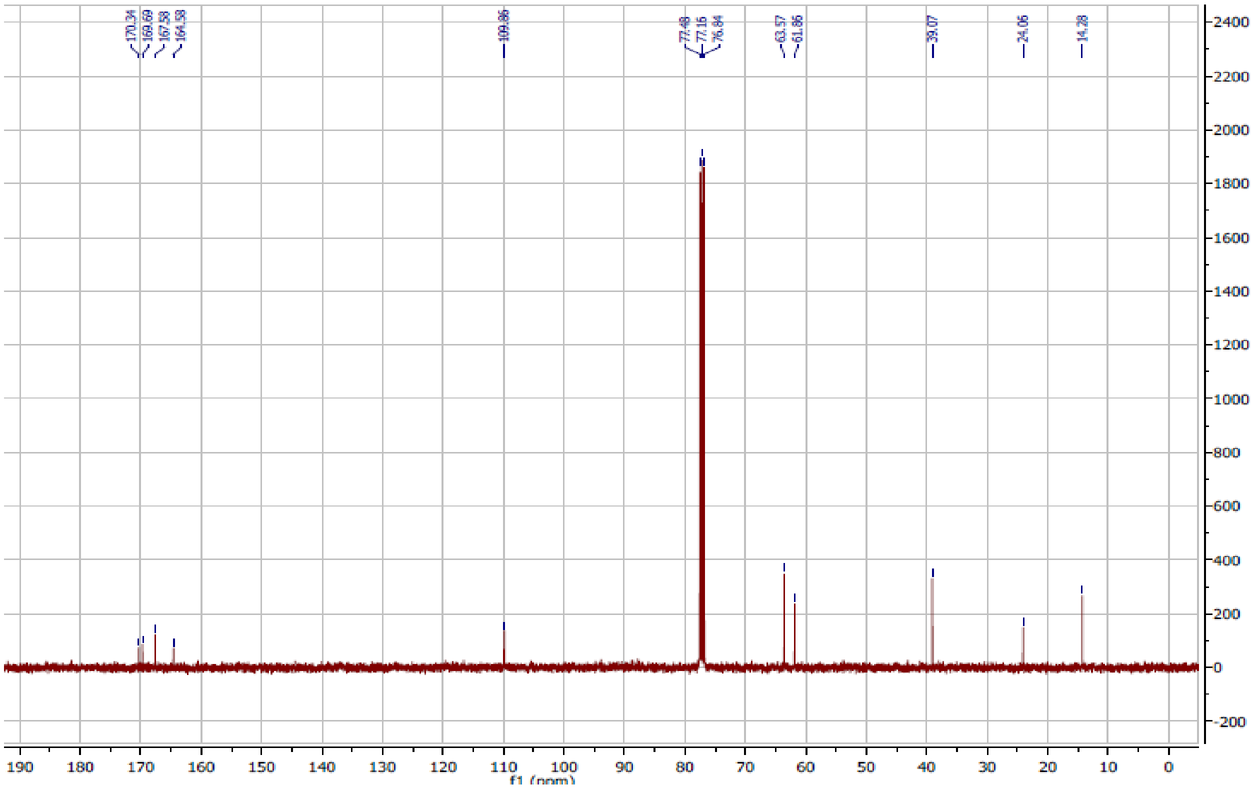

Figure S6: ^{13}C NMR spectrum of compound **5** ($CDCl_3$).

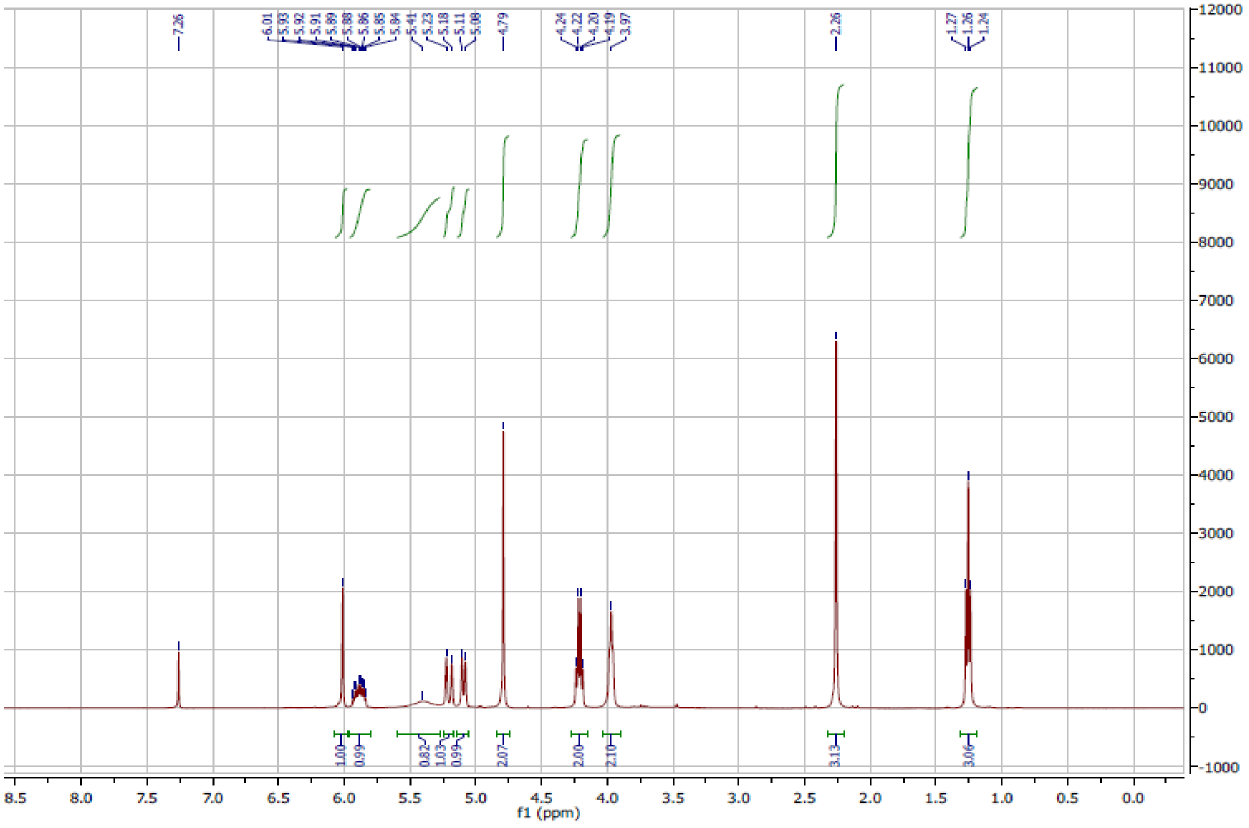

Figure S7: ^{1}H NMR spectrum of compound **6** ($CDCl_3$).

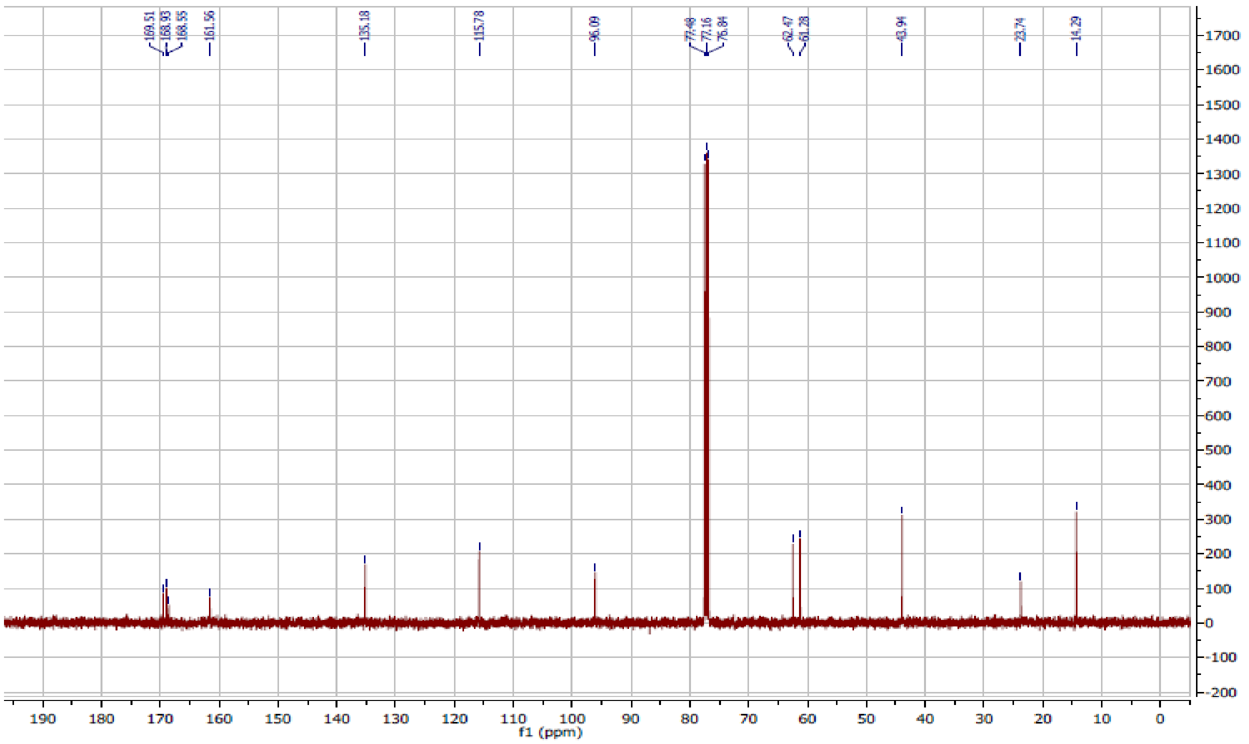

Figure S8: ^{13}C NMR spectrum of compound **6** ($CDCl_3$).

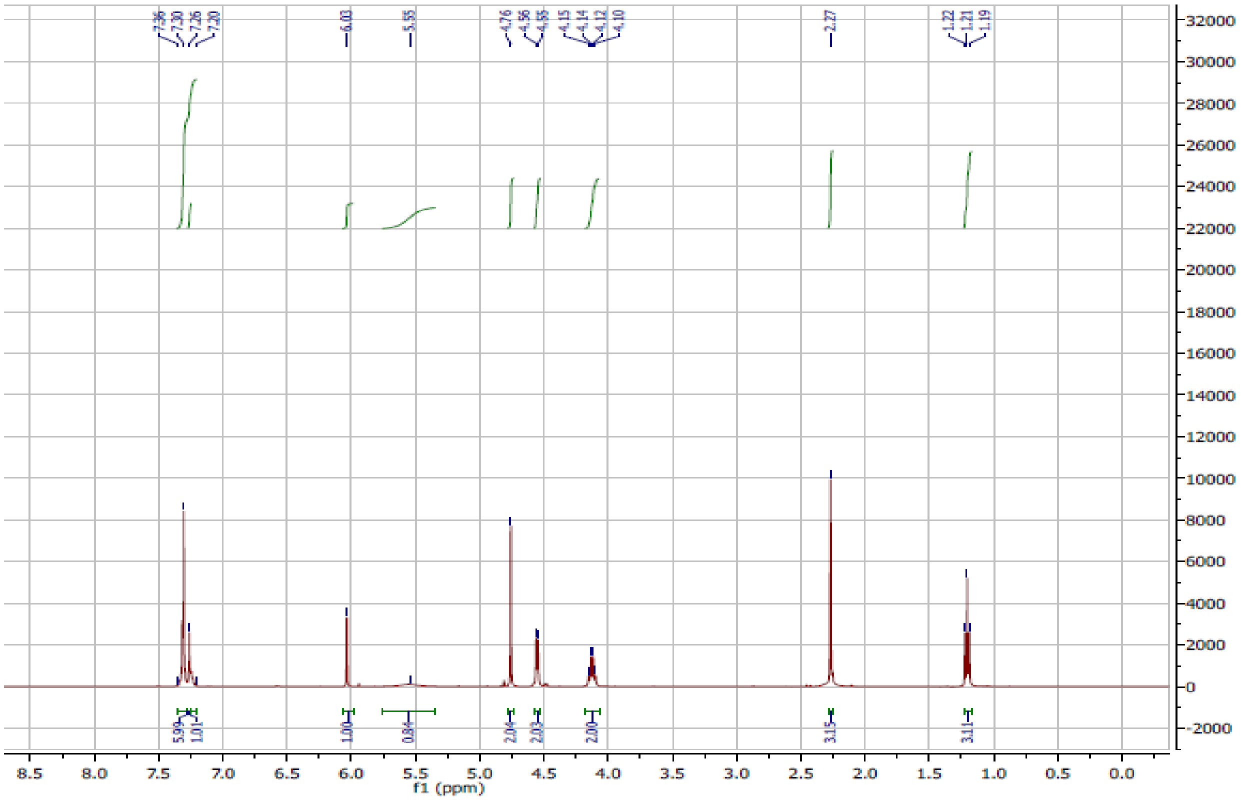

Figure S9: ^{1}H NMR spectrum of compound **7** ($CDCl_3$).

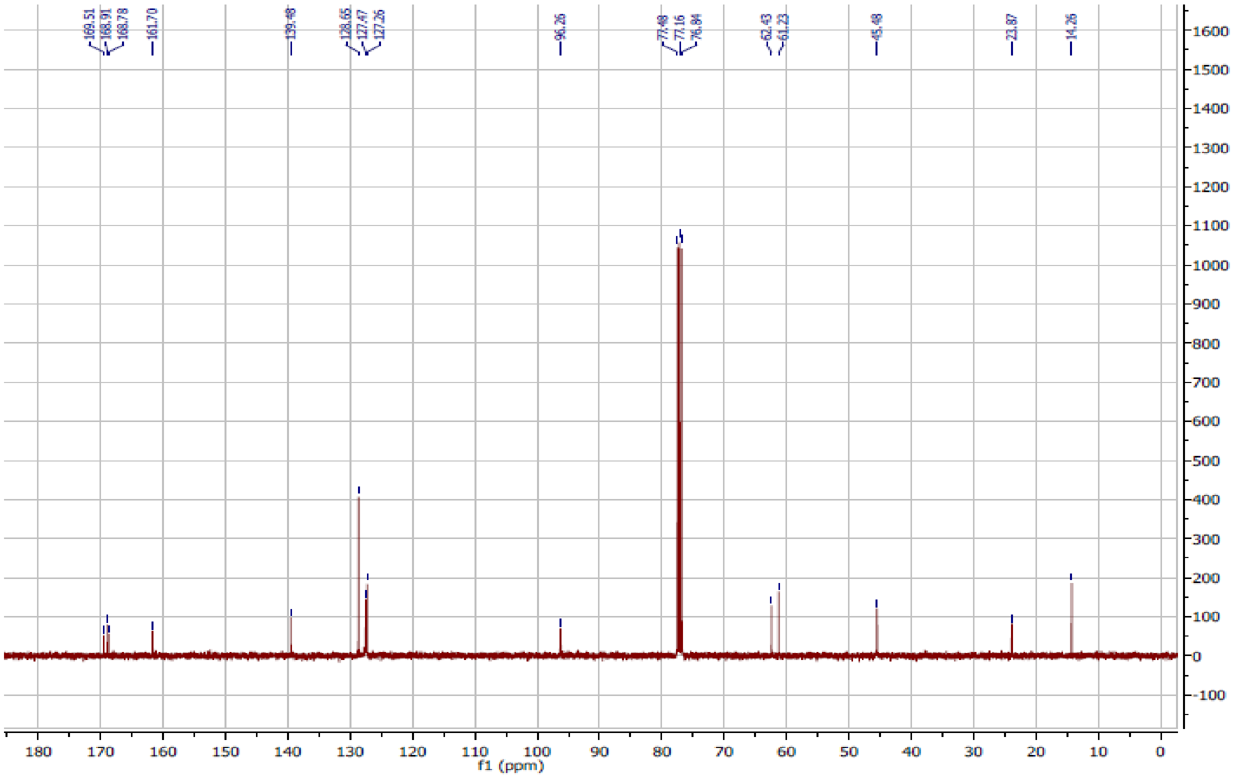

Figure S10: ^{13}C NMR spectrum of compound **7** ($CDCl_3$).

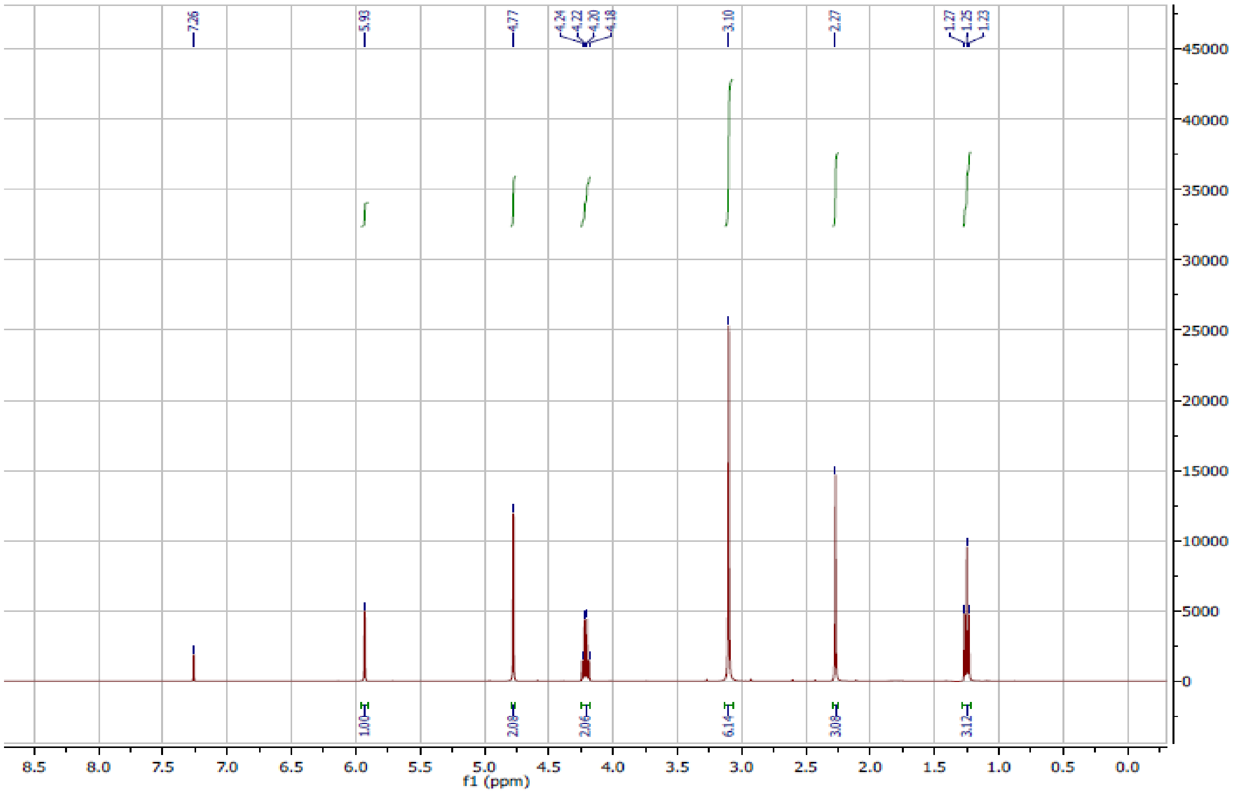

Figure S11: ^{1}H NMR spectrum of compound **8** ($CDCl_3$).

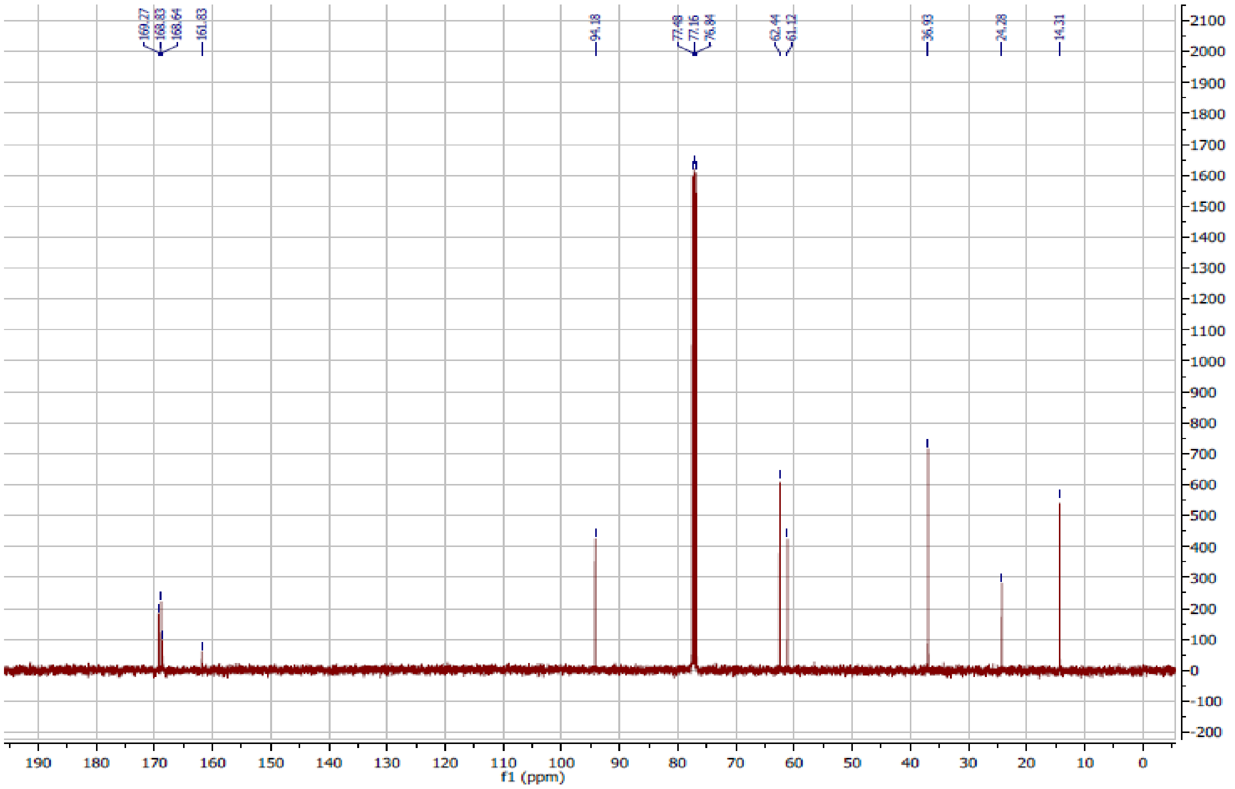

Figure S12: ^{13}C NMR spectrum of compound **8** ($CDCl_3$).

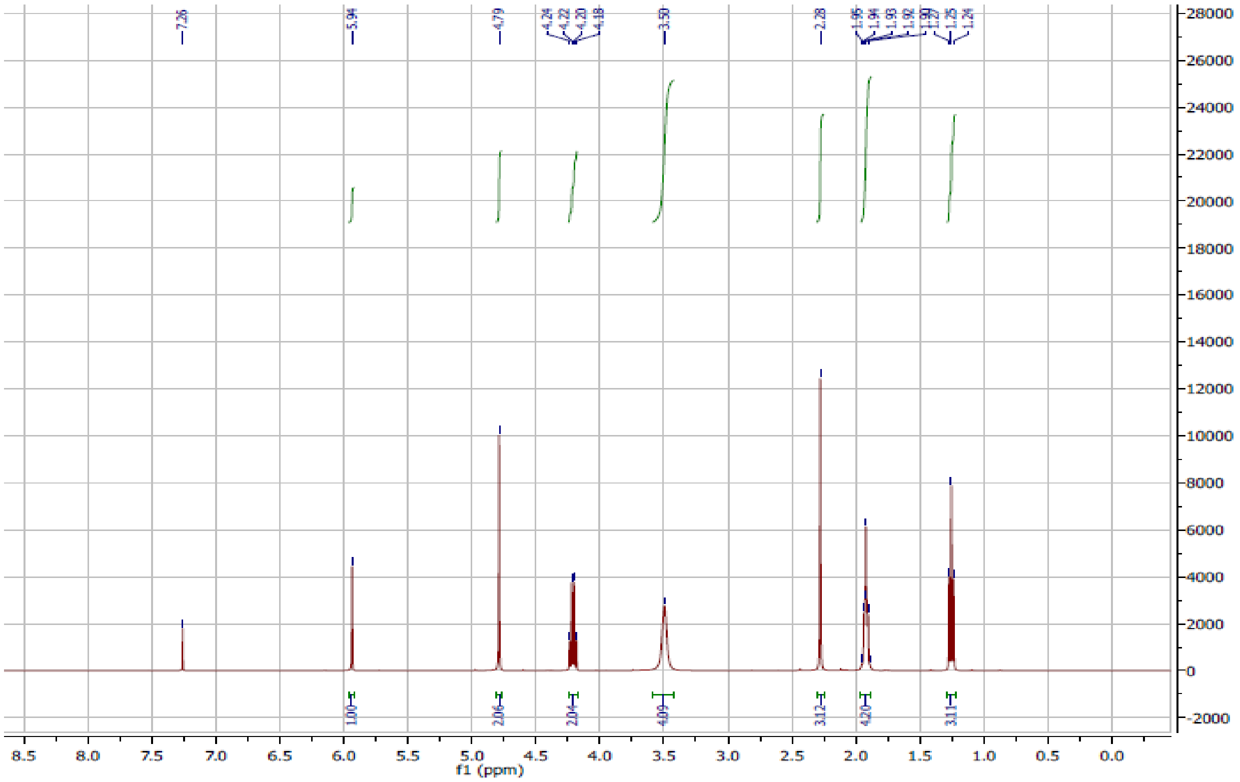

Figure S13: ^{1}H NMR spectrum of compound **9** ($CDCl_3$).

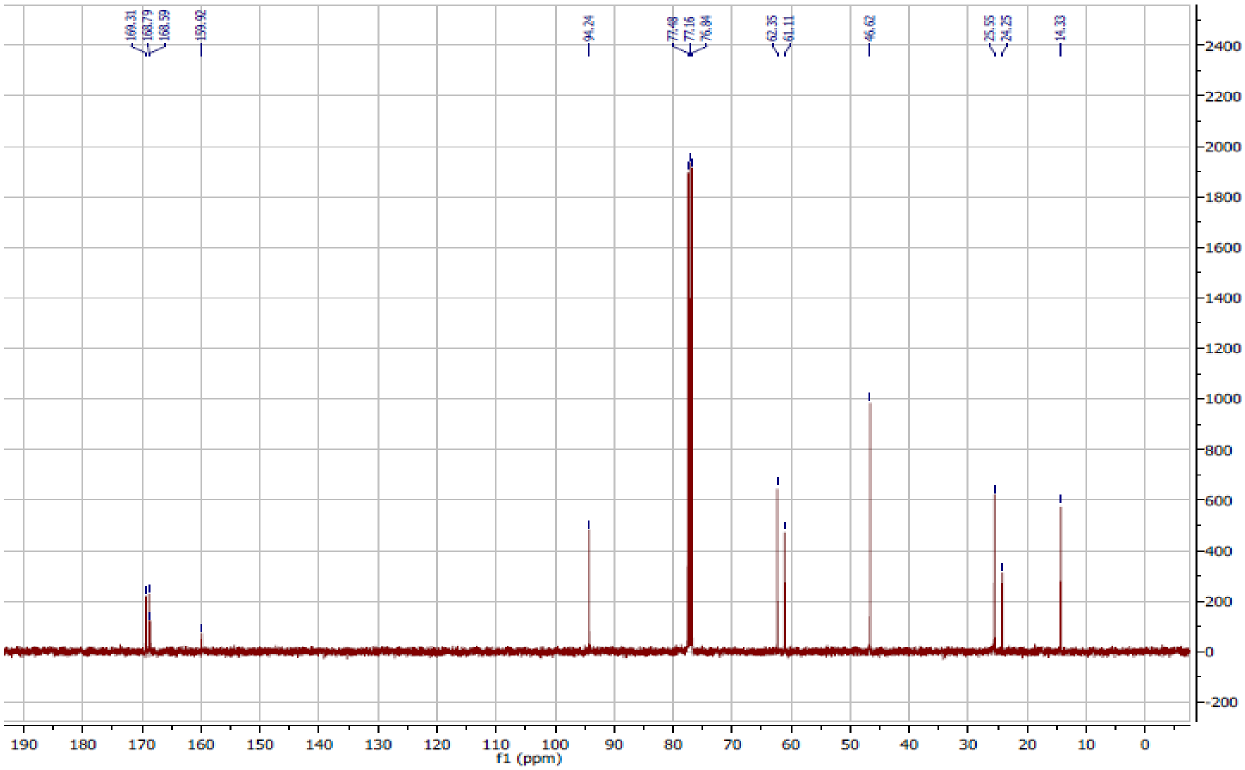

Figure S14: ^{13}C NMR spectrum of compound **9** ($CDCl_3$).

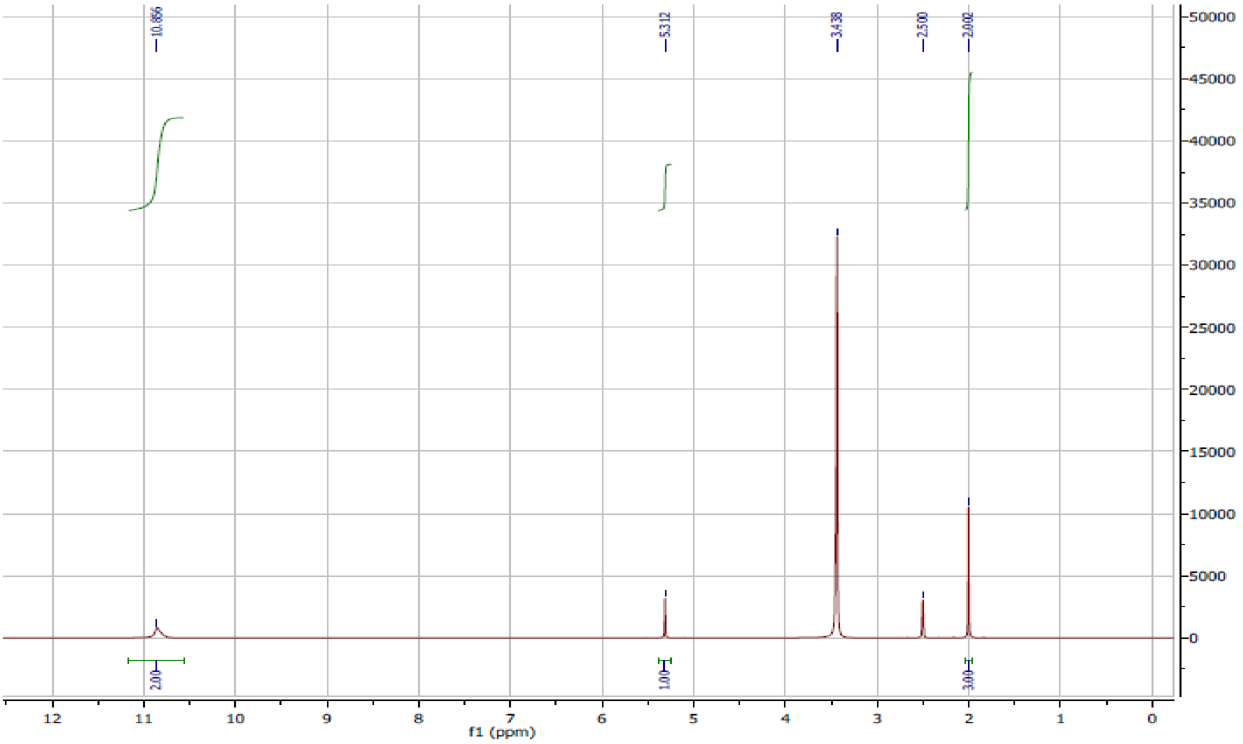

Figure S15: ^{1}H NMR spectrum of compound **10** (DMSO-d_6).

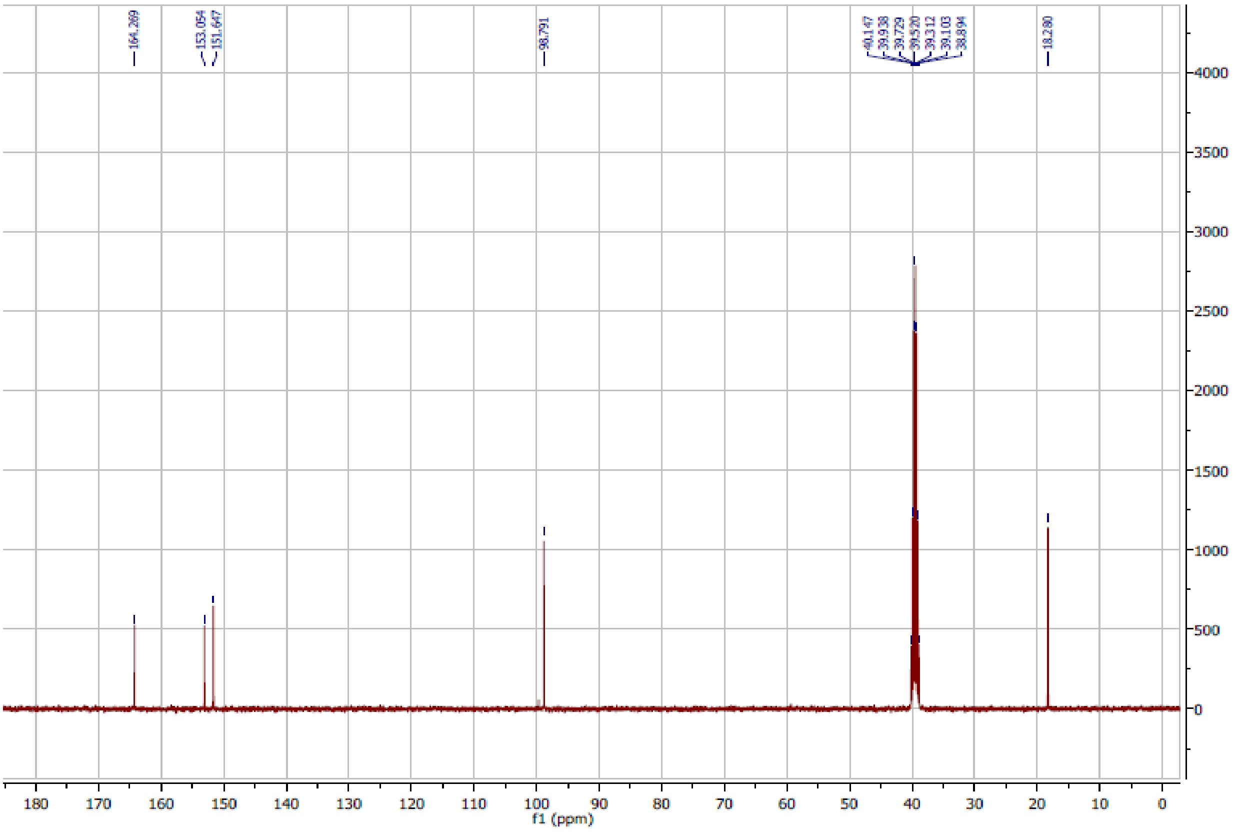

Figure S16: ^{13}C NMR spectrum of compound **10** (DMSO-d_6).

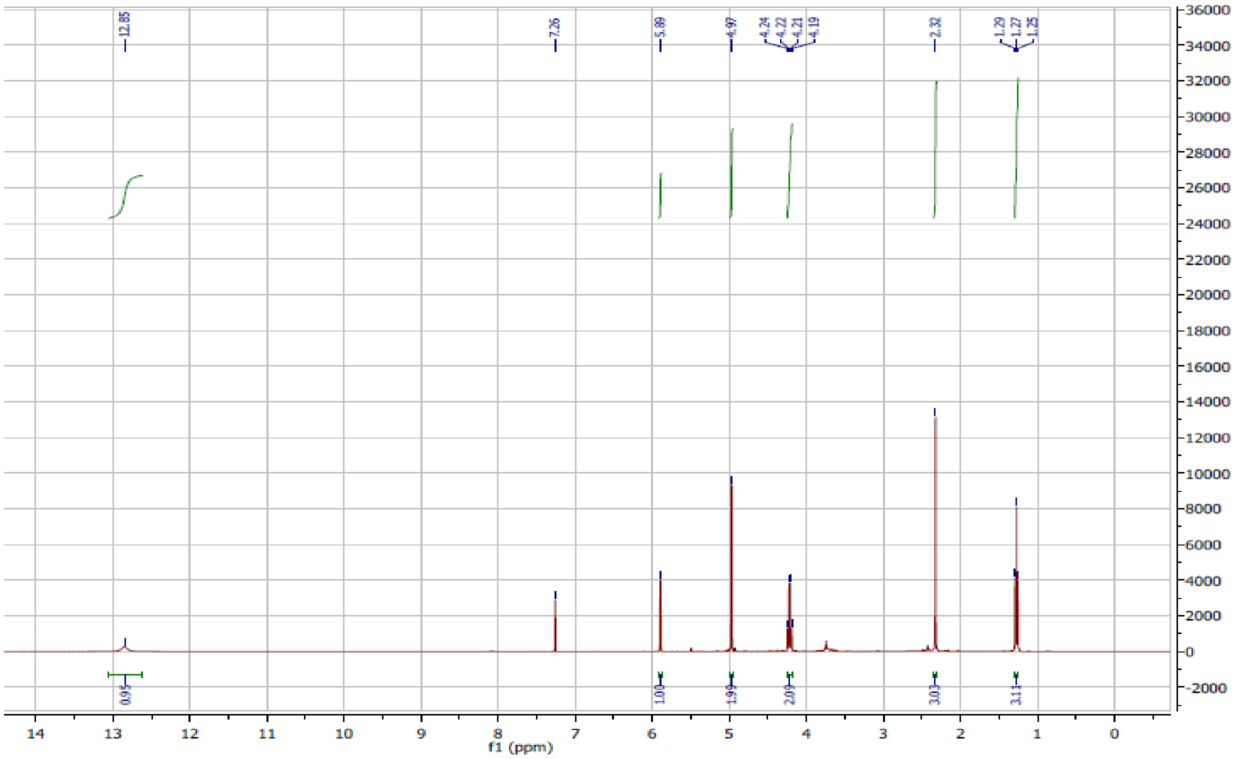

Figure S17: ^{1}H NMR spectrum of compound **11** ($CDCl_3$).

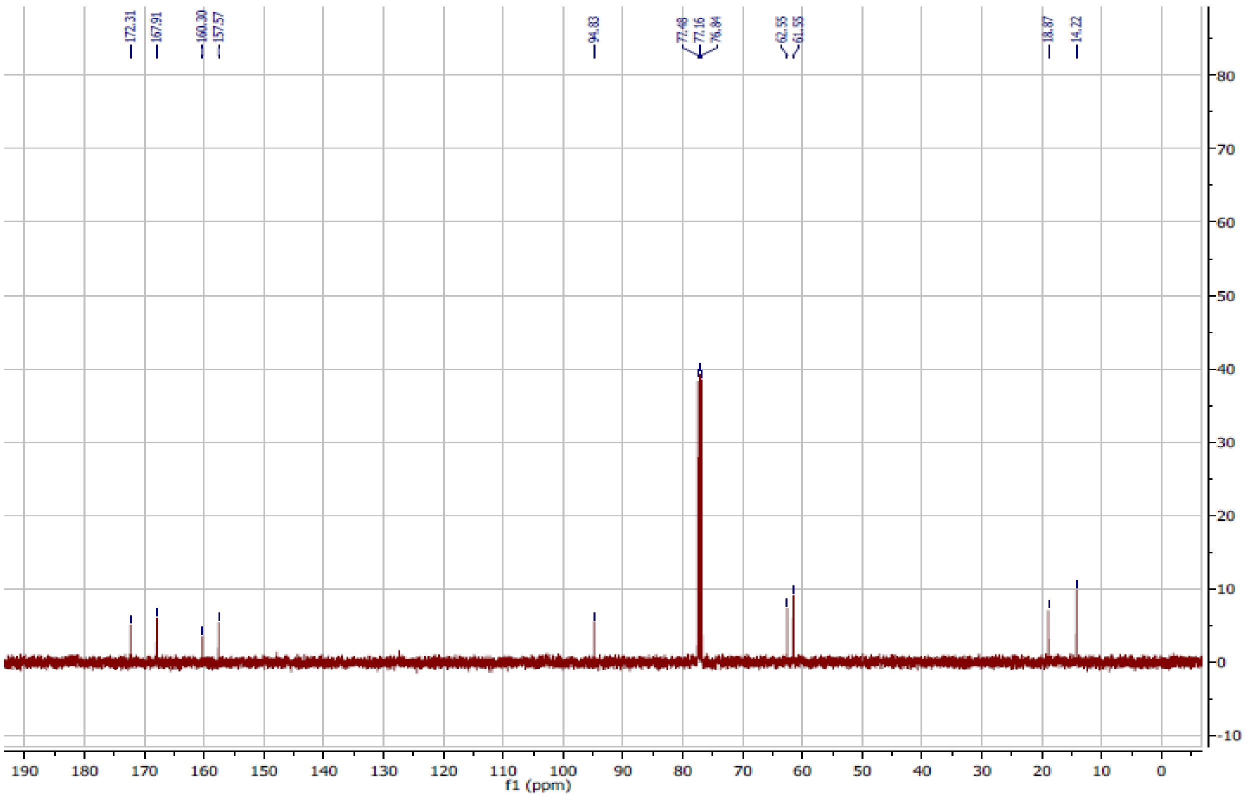

Figure S18: ^{13}C NMR spectrum of compound **11** ($CDCl_3$).

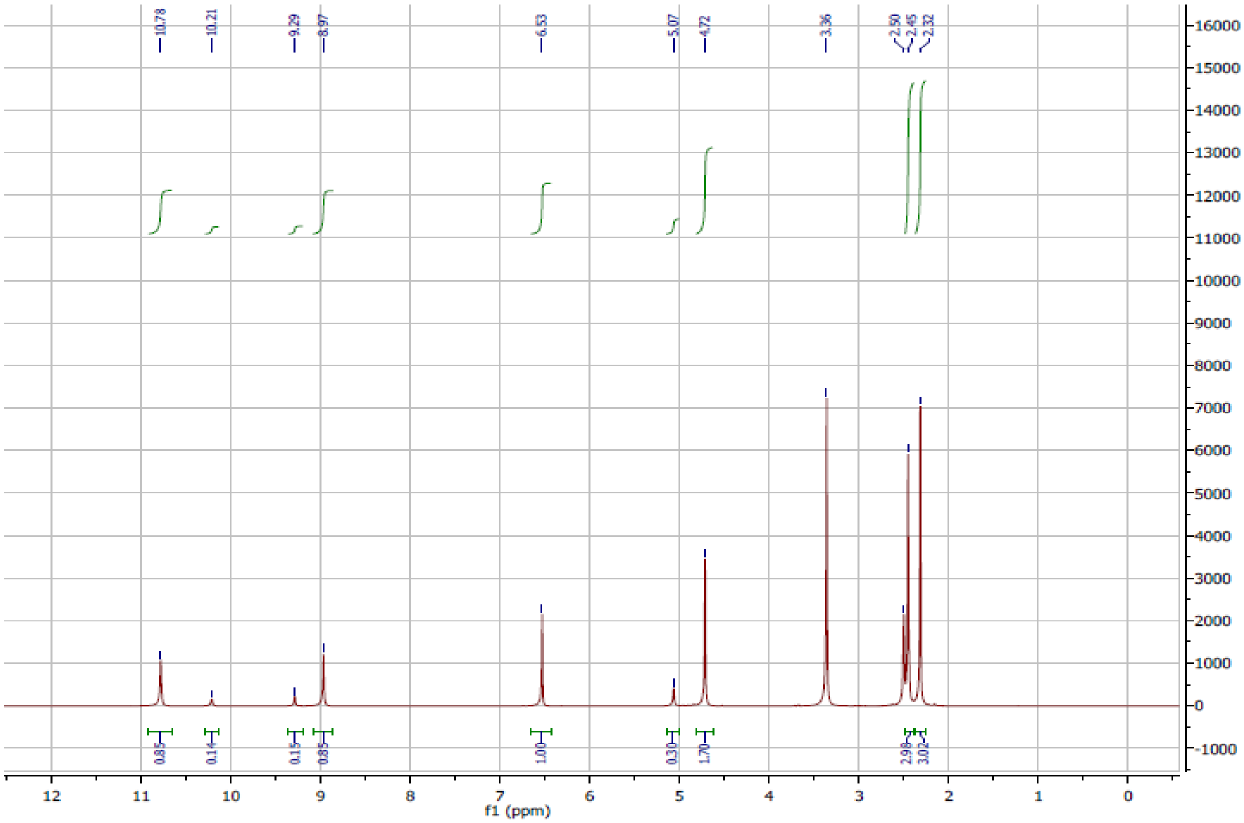

Figure S19: ^{1}H NMR spectrum of compound **12** (DMSO-d_6).

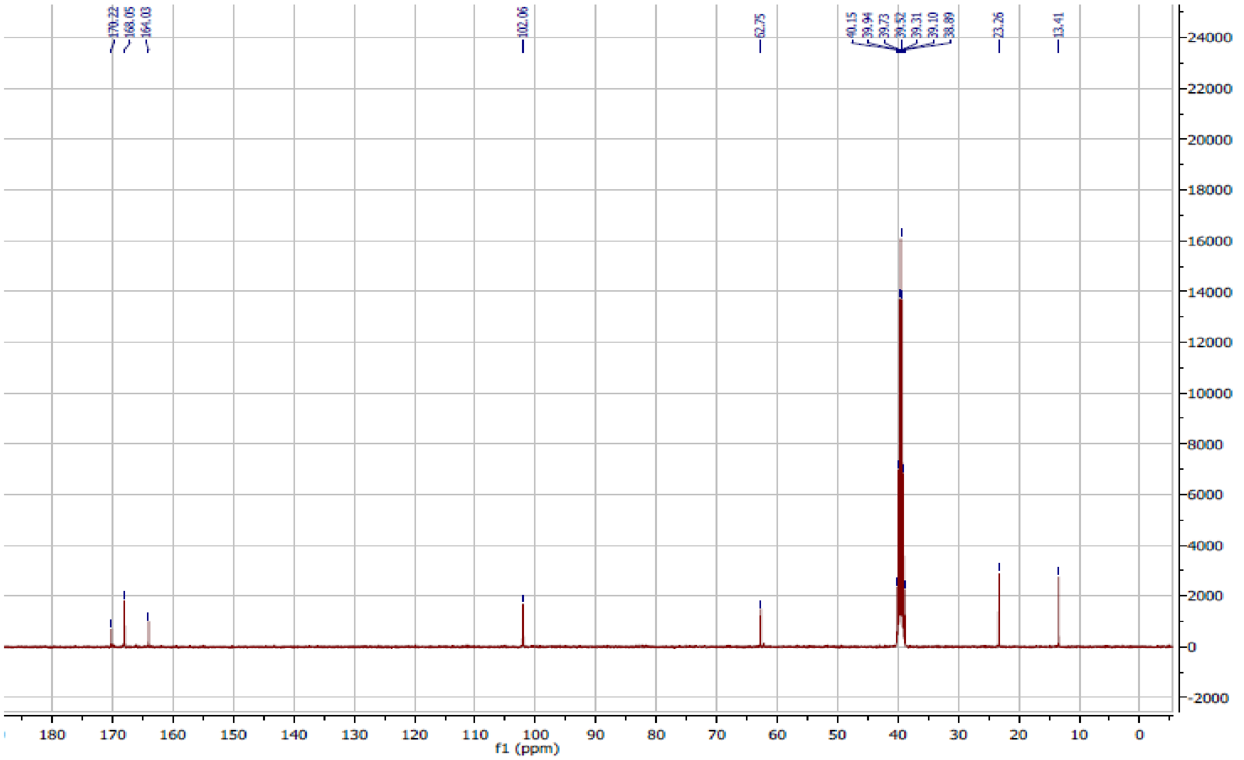

Figure S20: ^{13}C NMR spectrum of compound **12** (DMSO-d_6).

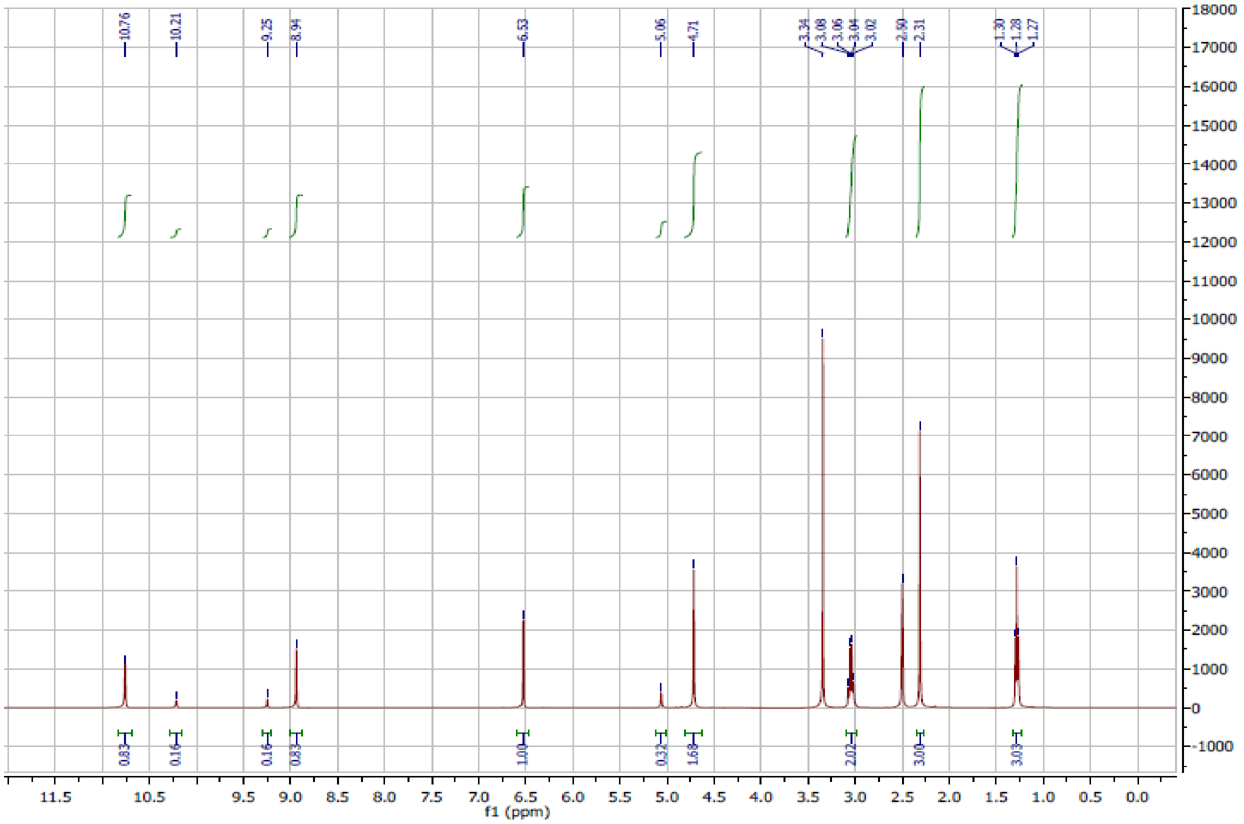

Figure S21: ^{1}H NMR spectrum of compound **13** (DMSO-d_6).

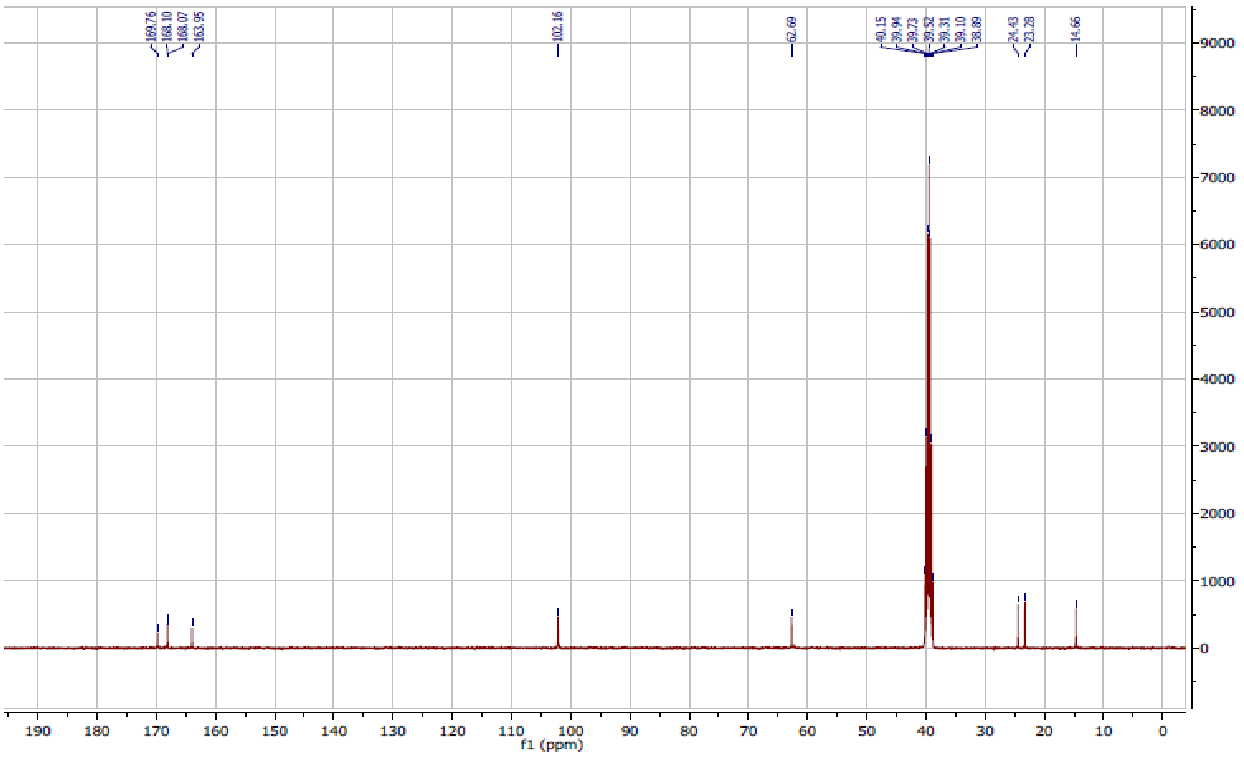

Figure S22: ^{13}C NMR spectrum of compound **13** (DMSO-d_6).

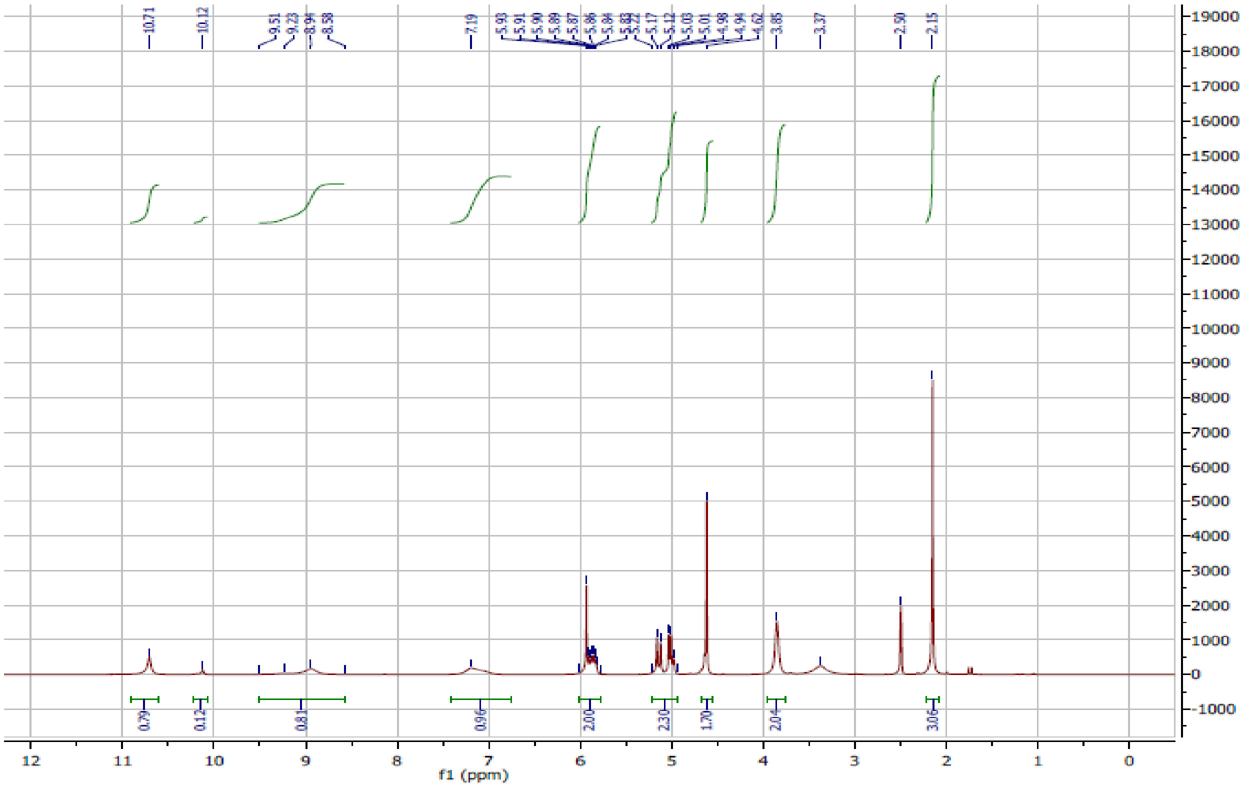

Figure S23: ^{1}H NMR spectrum of compound **14** (DMSO-d_6).

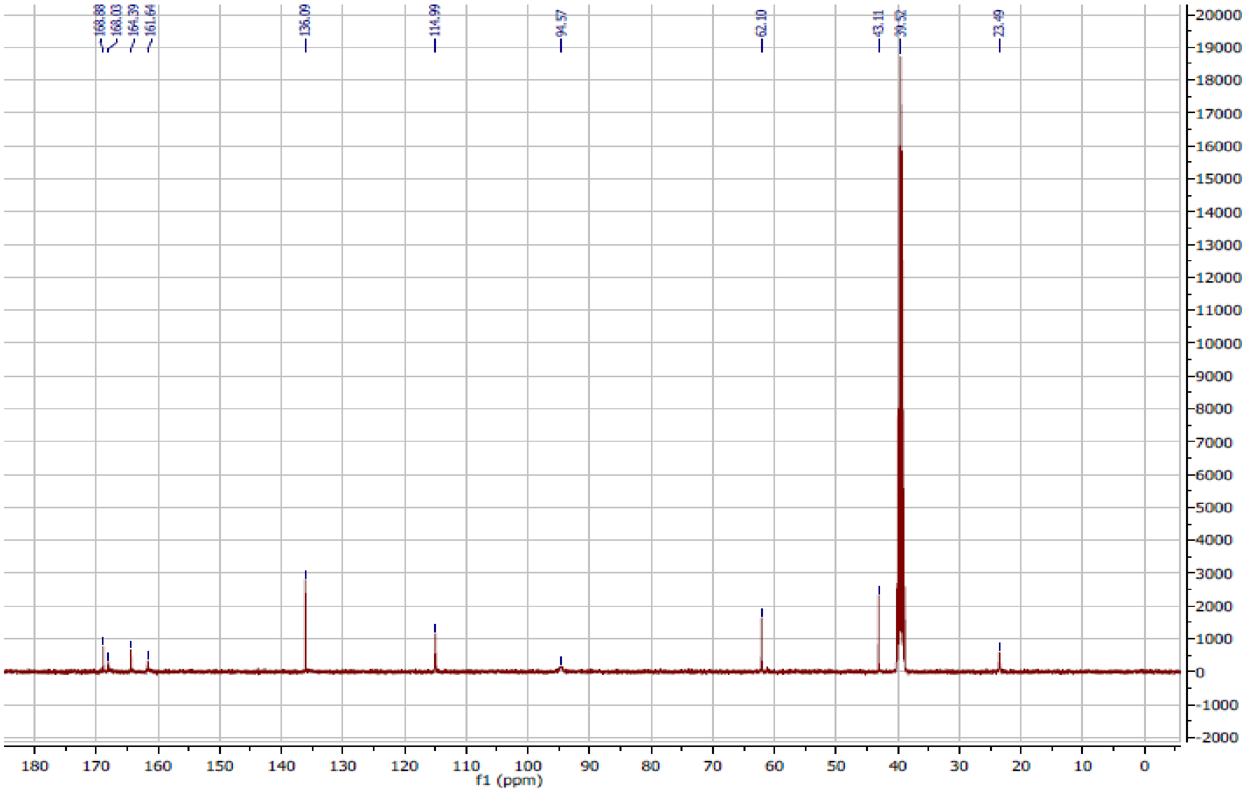

Figure S24: ^{13}C NMR spectrum of compound **14** (DMSO-d_6).

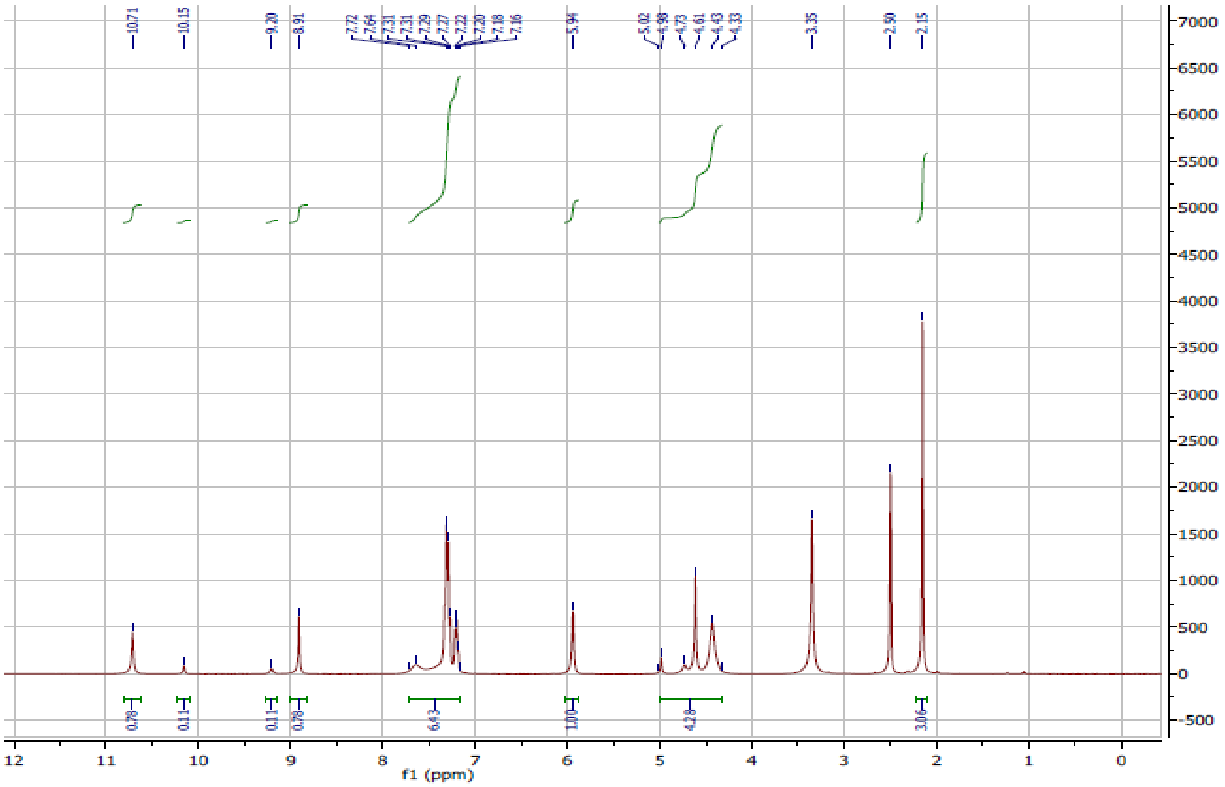

Figure S25: ^{1}H NMR spectrum of compound **15** (DMSO-d_6).

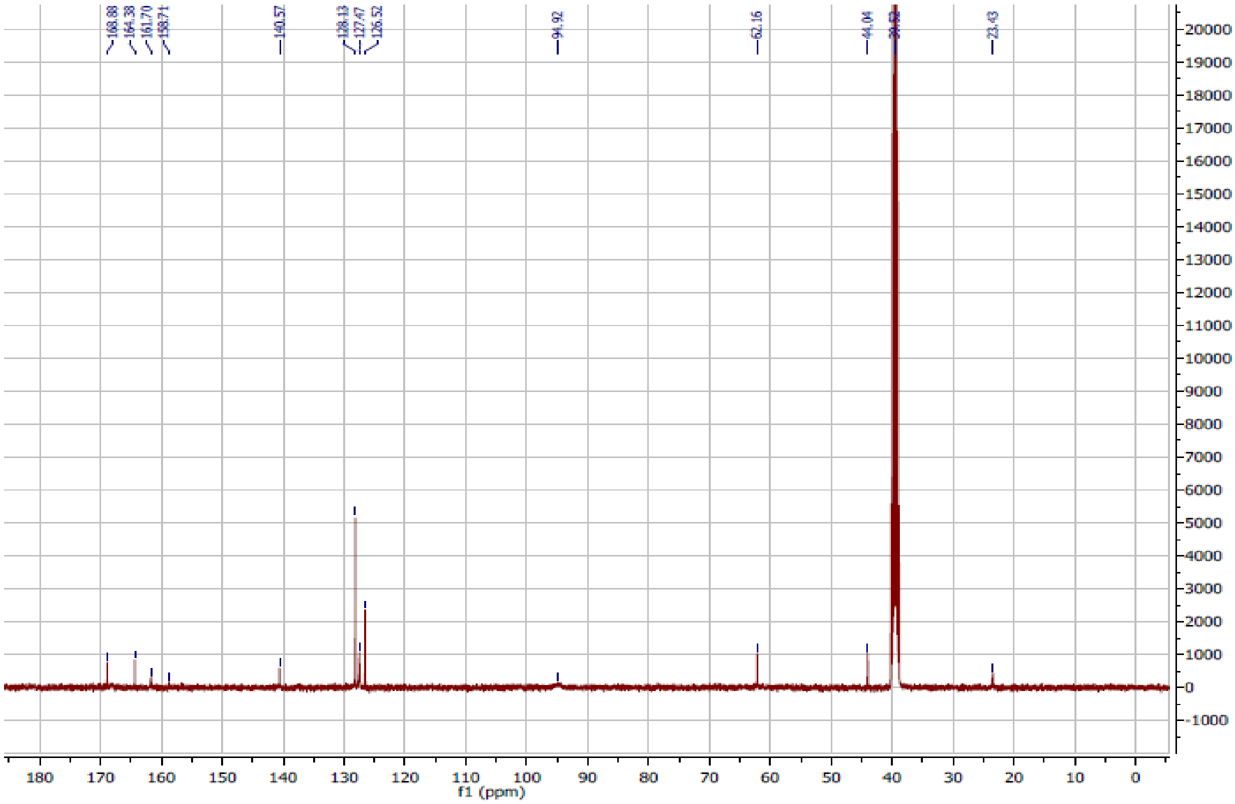

Figure S26: ^{13}C NMR spectrum of compound **15** (DMSO-d_6).

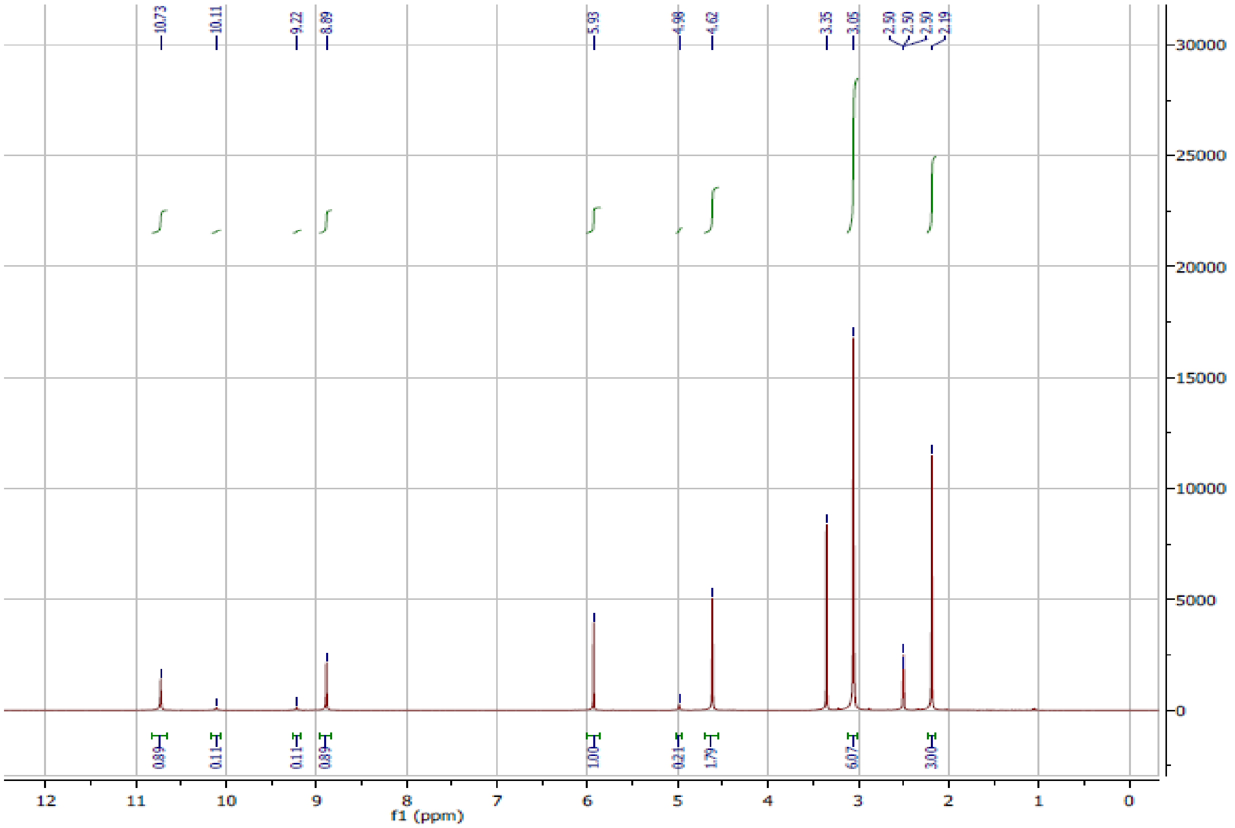

Figure S27: ^{1}H NMR spectrum of compound **16** (DMSO-d_6).

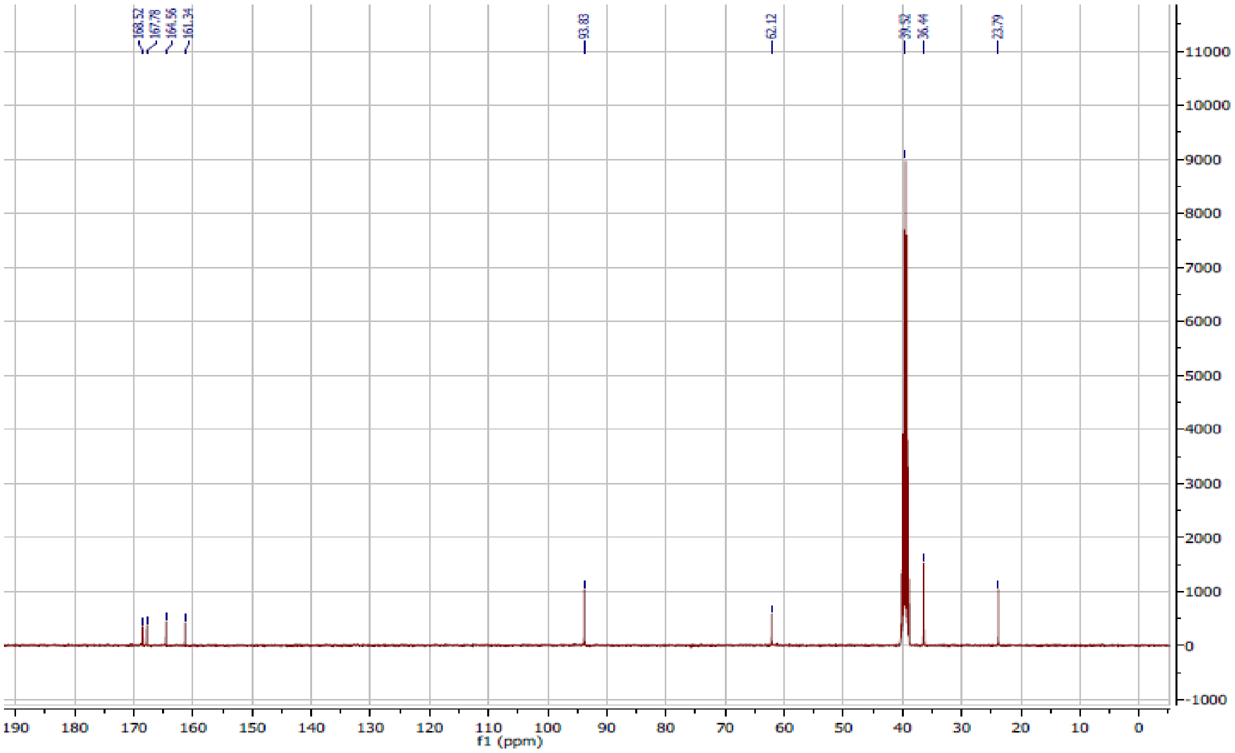

Figure S28: ^{13}C NMR spectrum of compound **16** (DMSO-d_6).

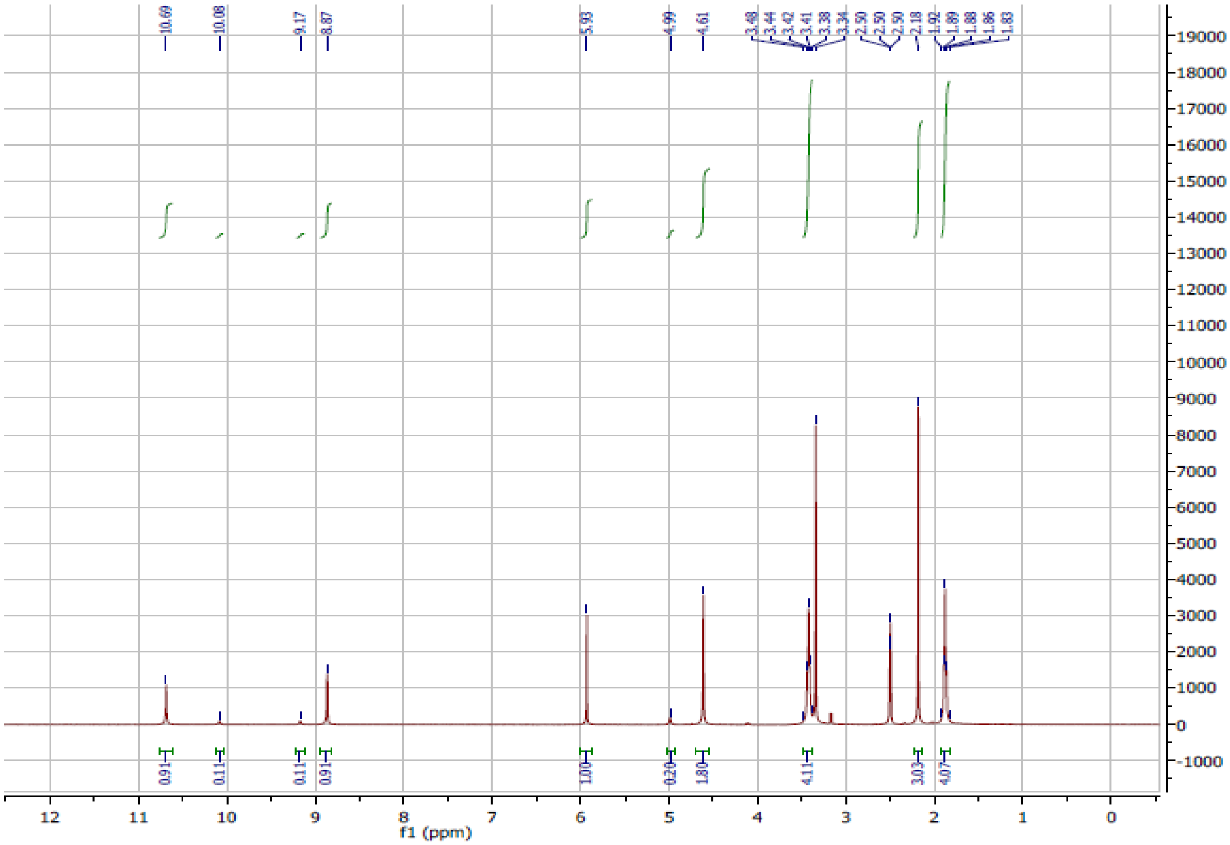

Figure S29: ^{1}H NMR spectrum of compound **17** (DMSO-d_6).

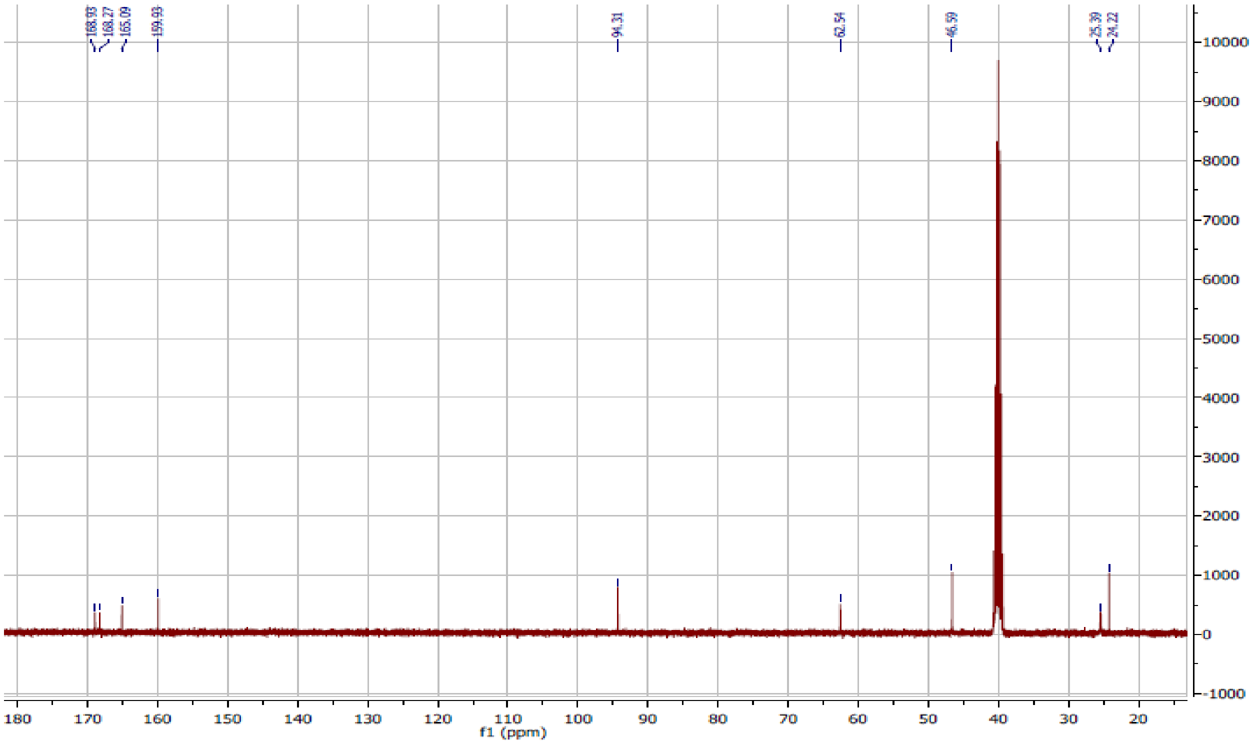

Figure S30: ^{13}C NMR spectrum of compound **17** (DMSO-d_6).

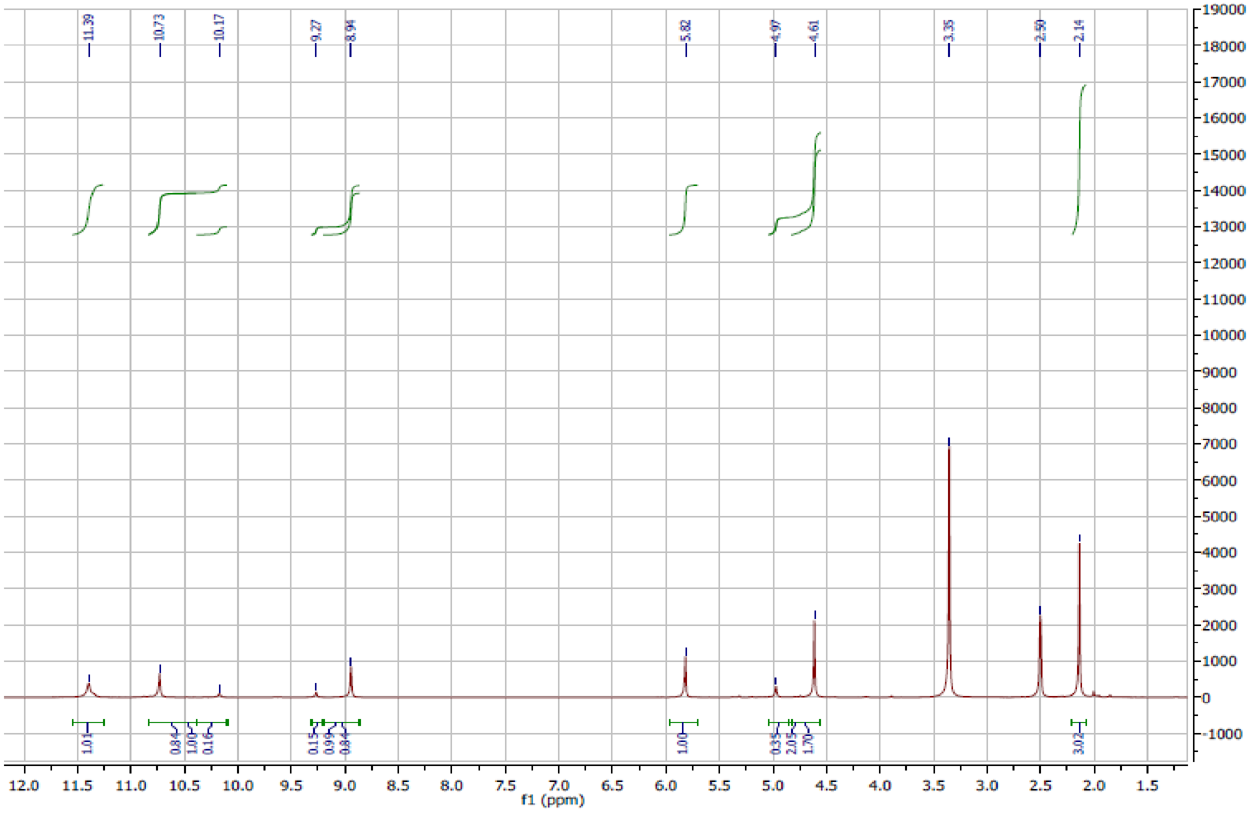

Figure S31: ^{1}H NMR spectrum of compound **18** (DMSO-d_6).

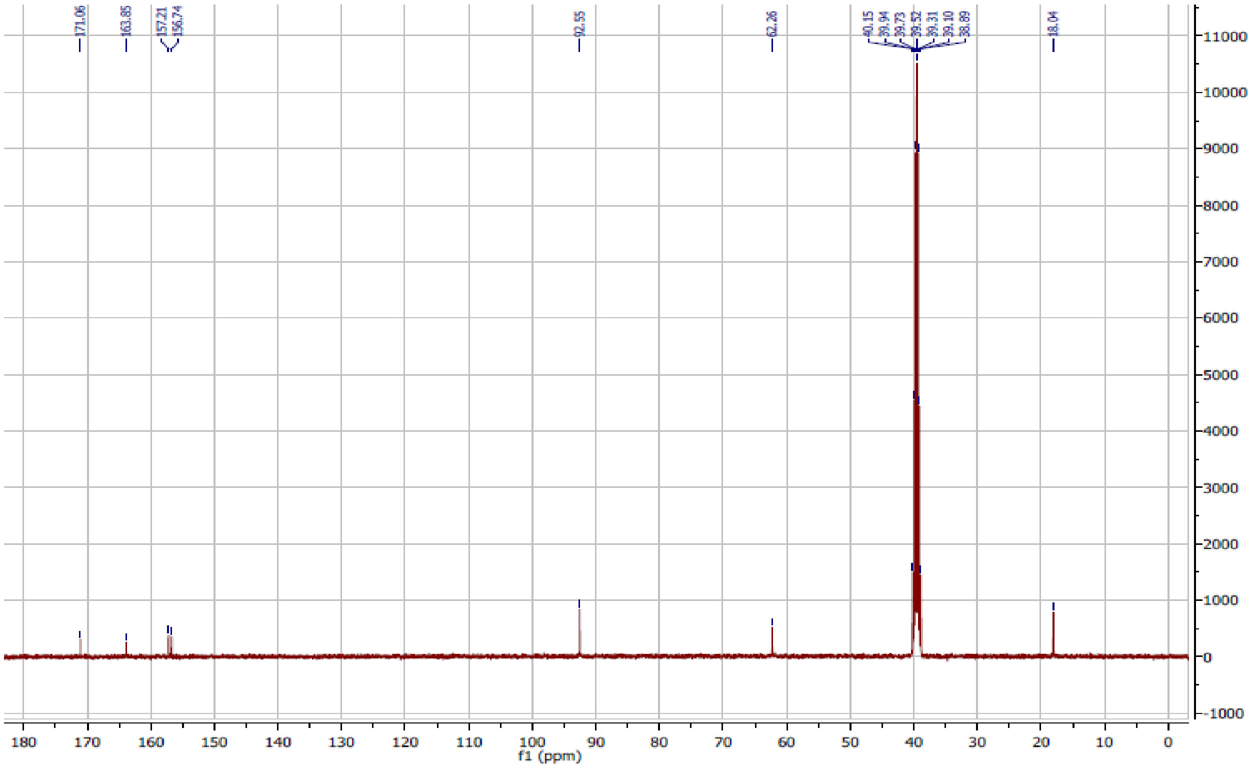

Figure S32: ^{13}C NMR spectrum of compound **18** (DMSO-d_6).

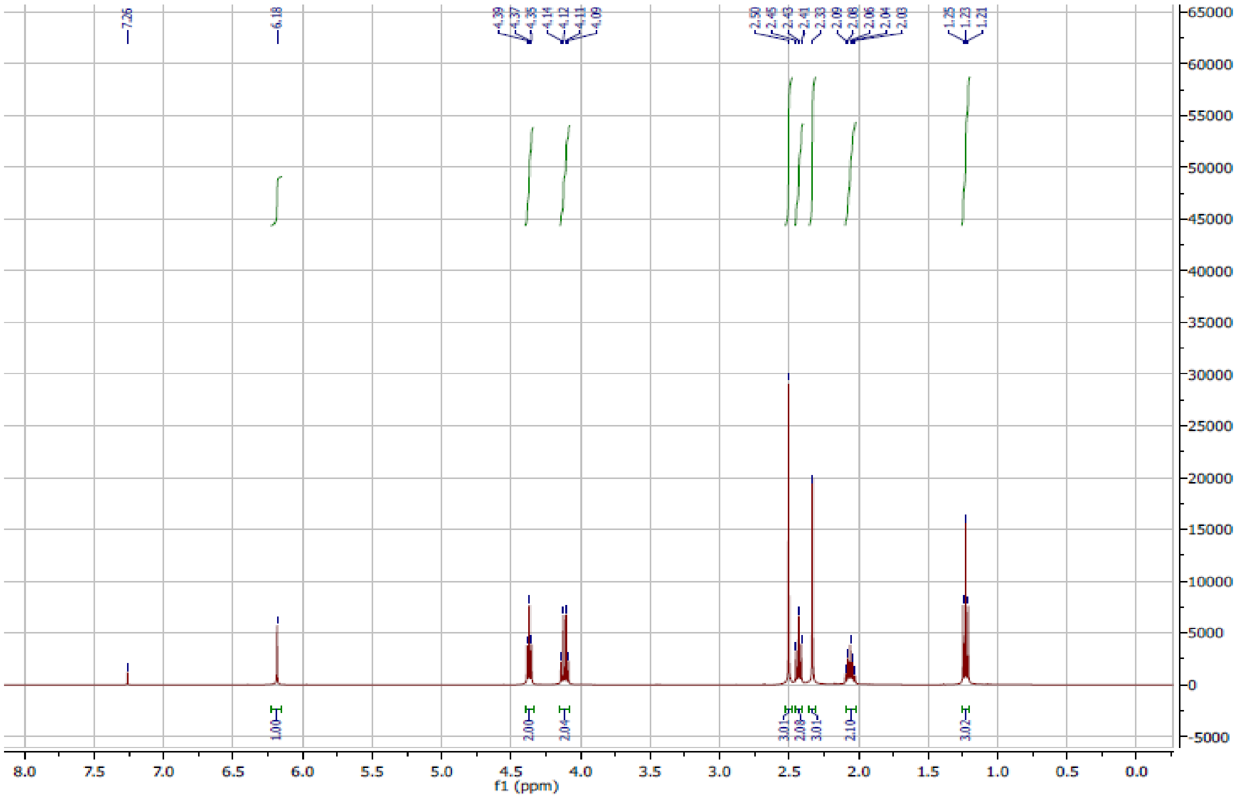

Figure S33: ^{1}H NMR spectrum of compound **20a** ($CDCl_3$).

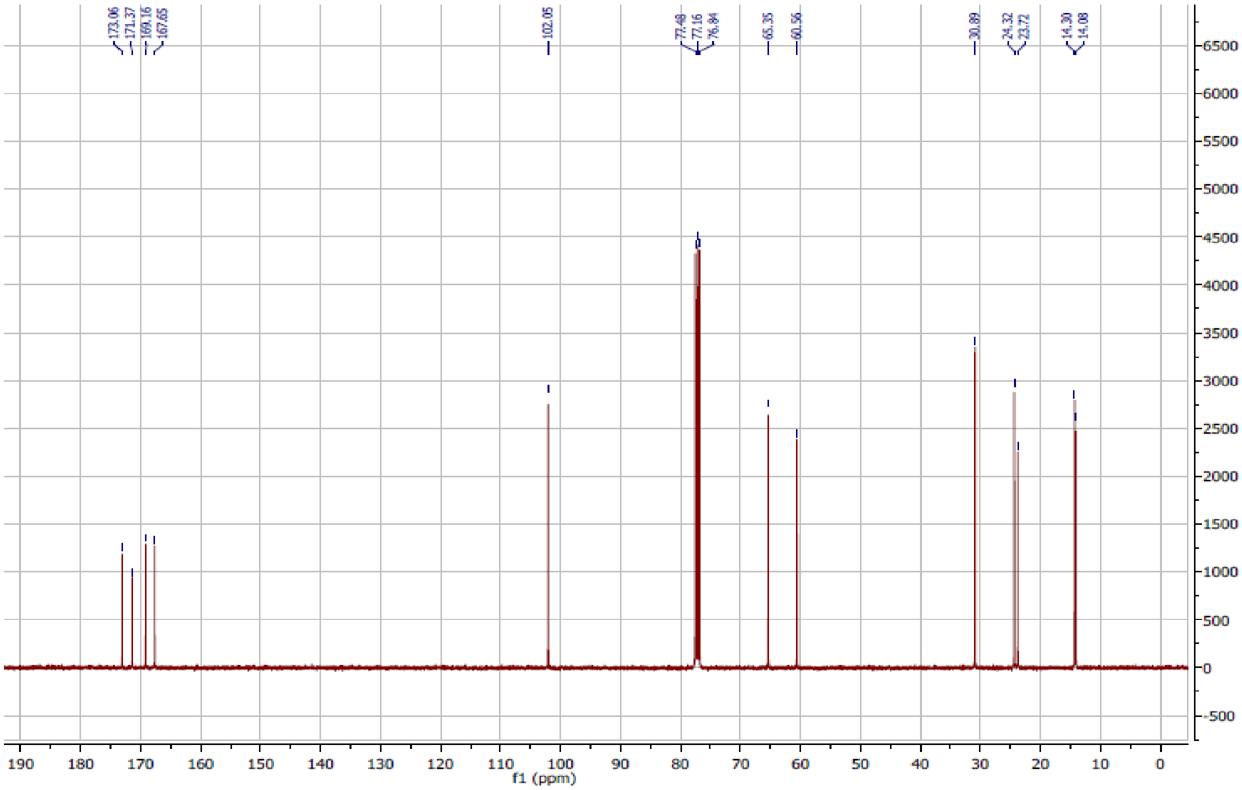

Figure S34: ^{13}C NMR spectrum of compound **20a** ($CDCl_3$).

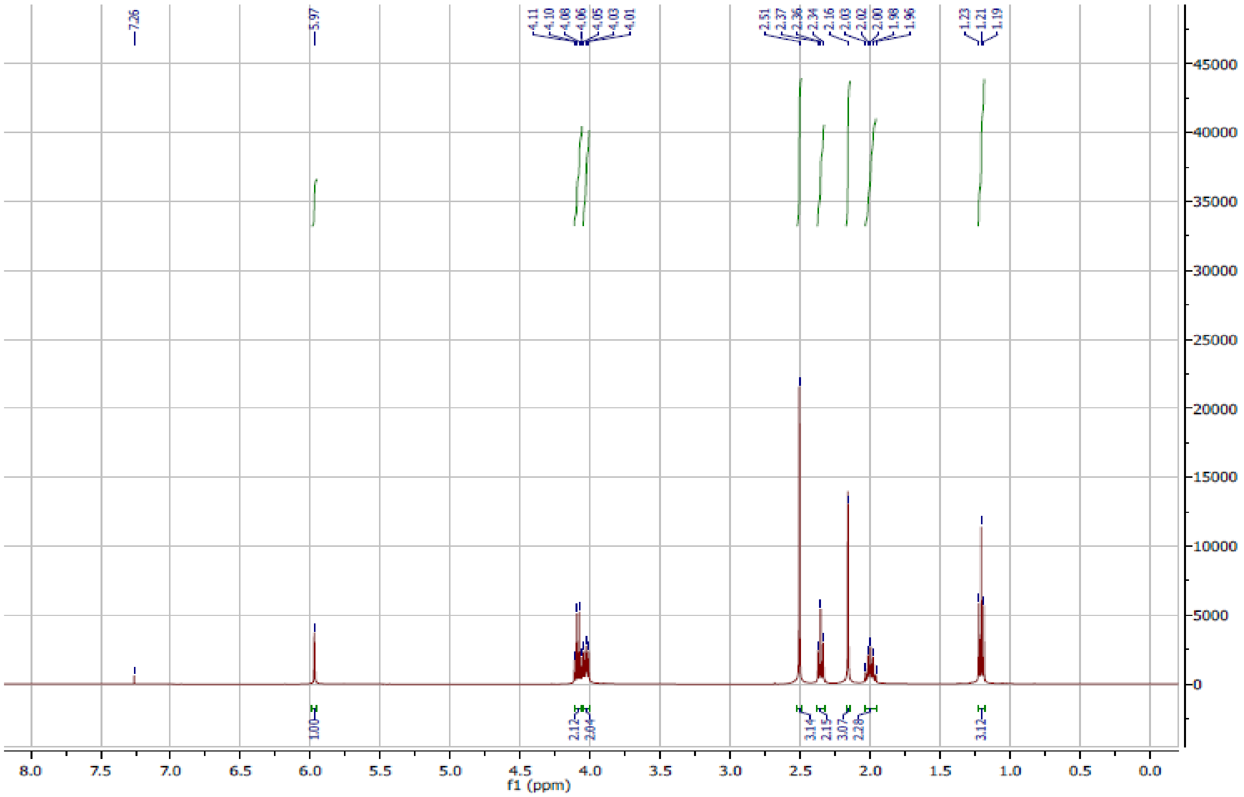

Figure S35: ^{1}H NMR spectrum of compound **20b** ($CDCl_3$).

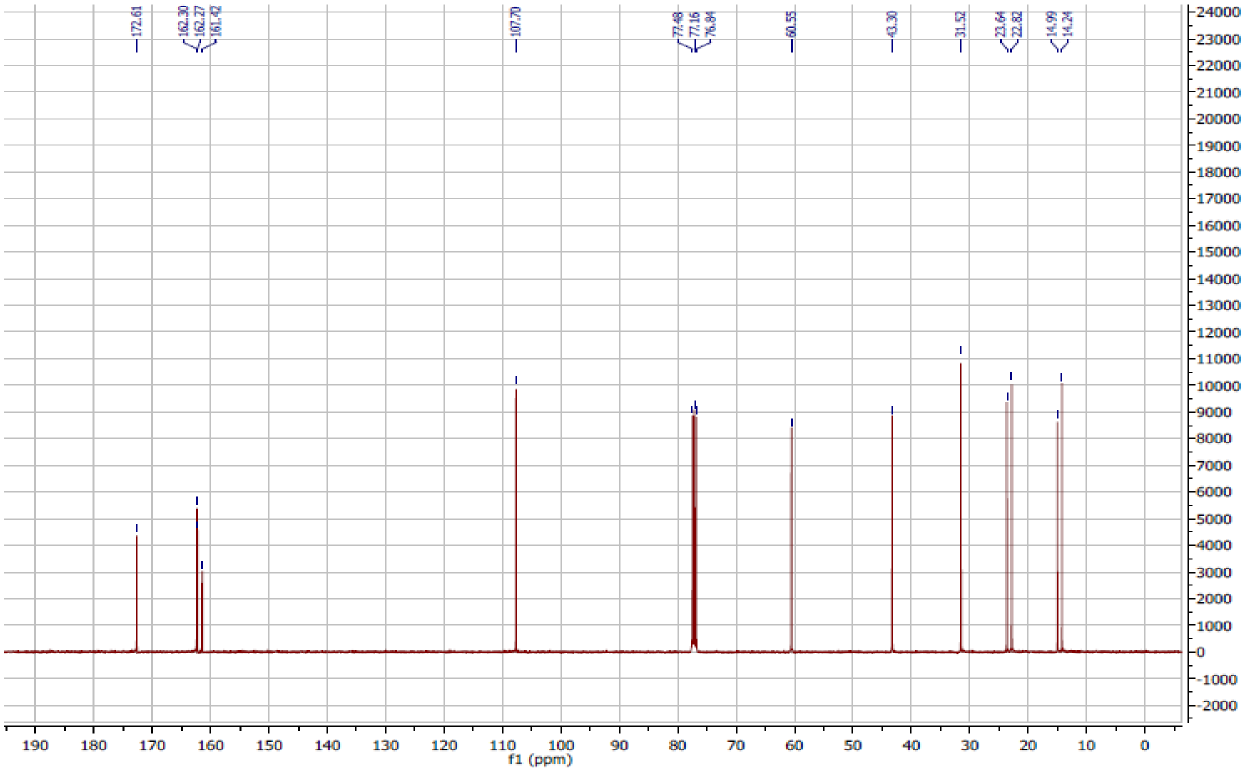

Figure S36: ^{13}C NMR spectrum of compound **20b** ($CDCl_3$).

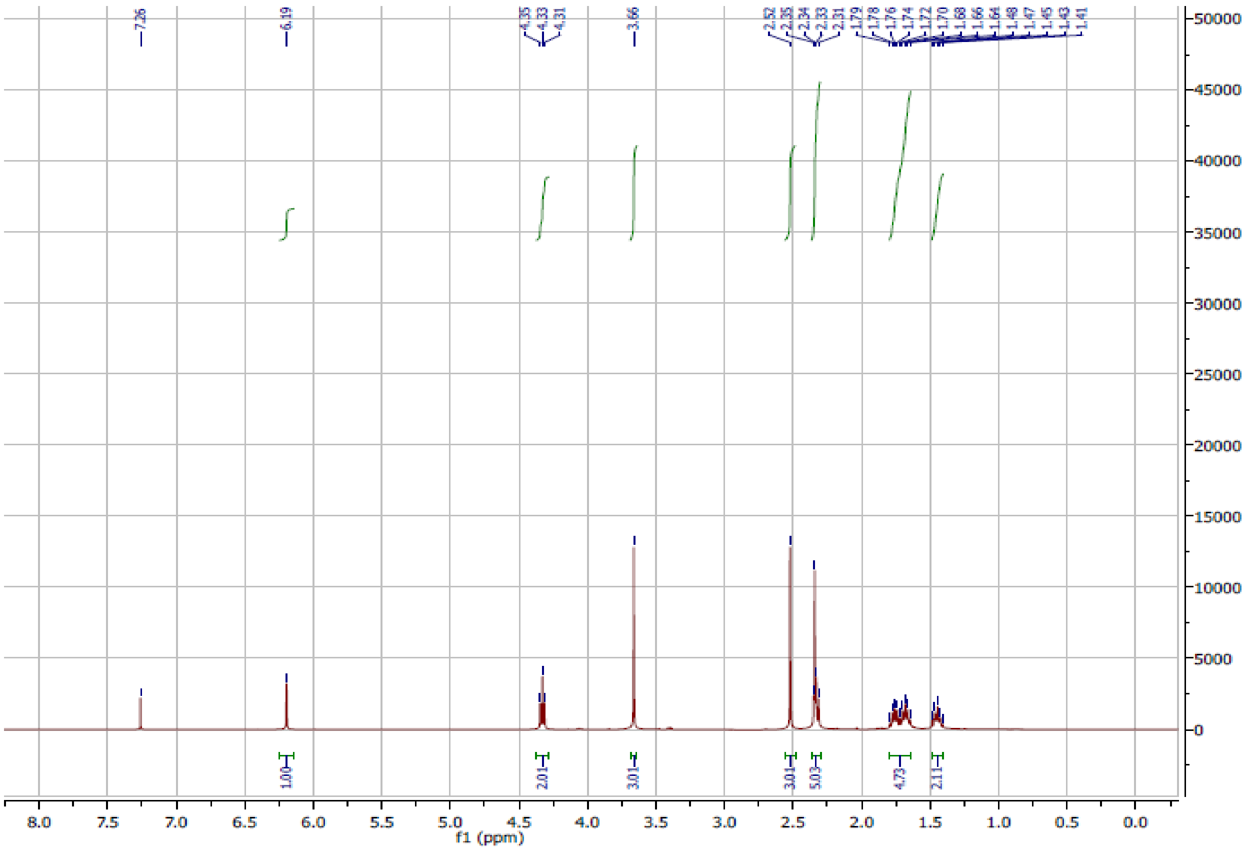

Figure S37: ^{1}H NMR spectrum of compound **21a** ($CDCl_3$).

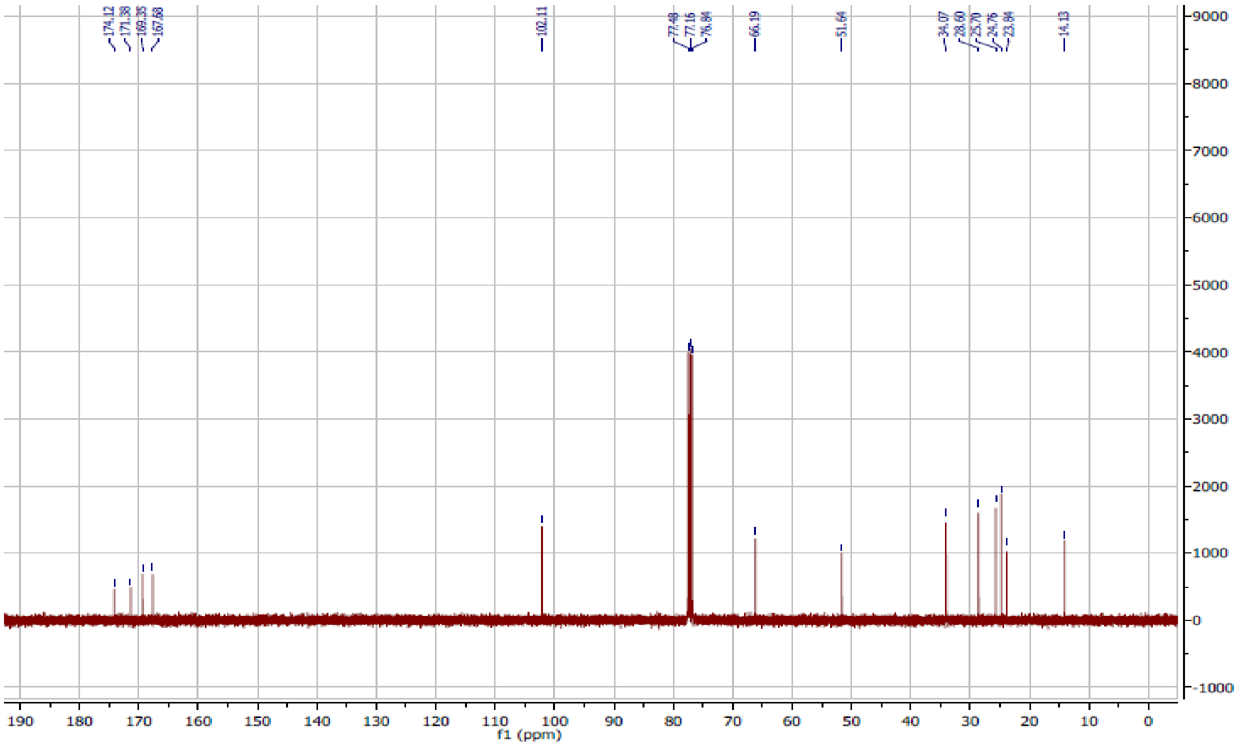

Figure S38: ^{13}C NMR spectrum of compound **21a** ($CDCl_3$).

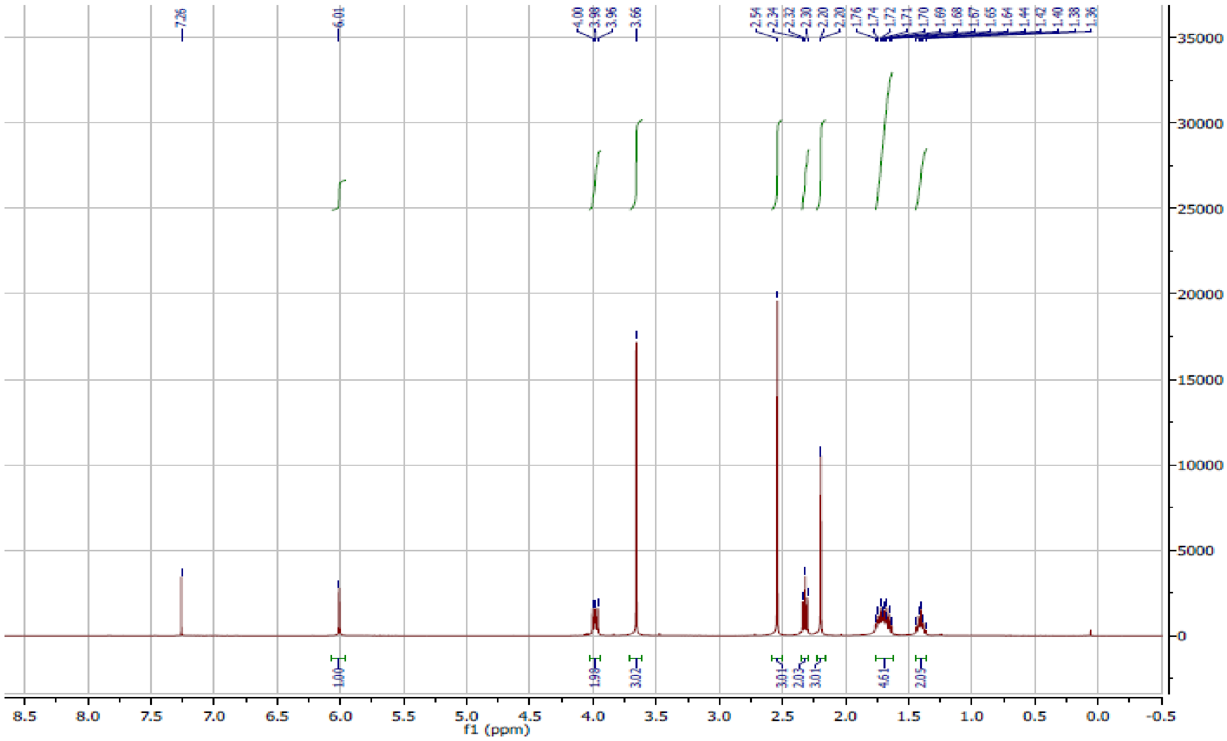

Figure S39: ^{1}H NMR spectrum of compound **21b** ($CDCl_3$).

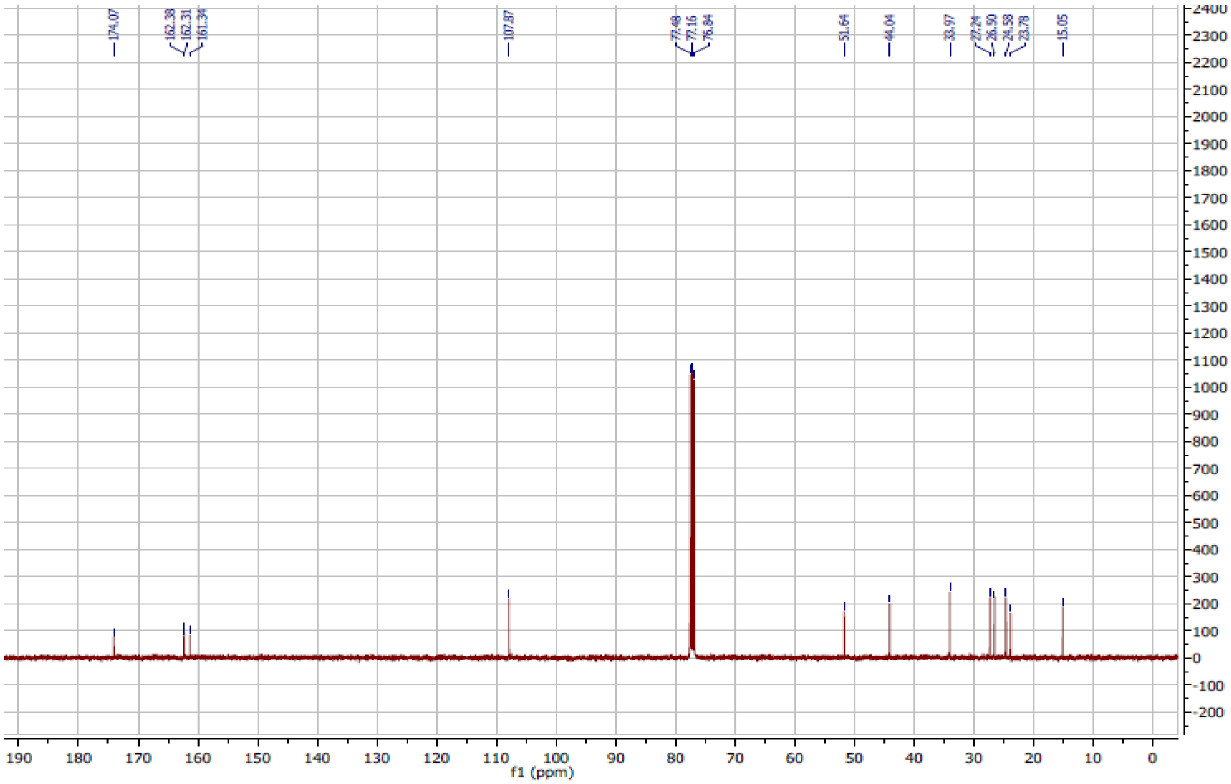

Figure S40: ^{13}C NMR spectrum of compound **21a** ($CDCl_3$).

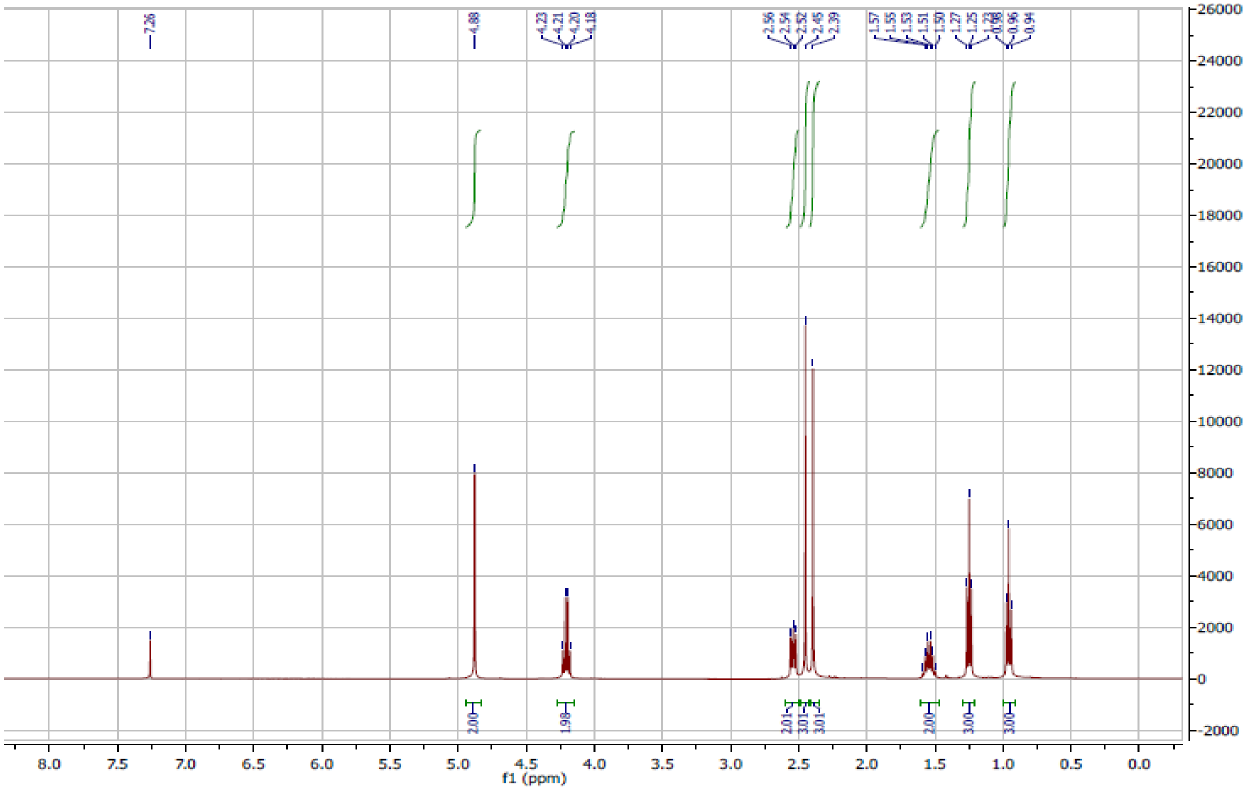

Figure S41: ^{1}H NMR spectrum of compound **22a** ($CDCl_3$).

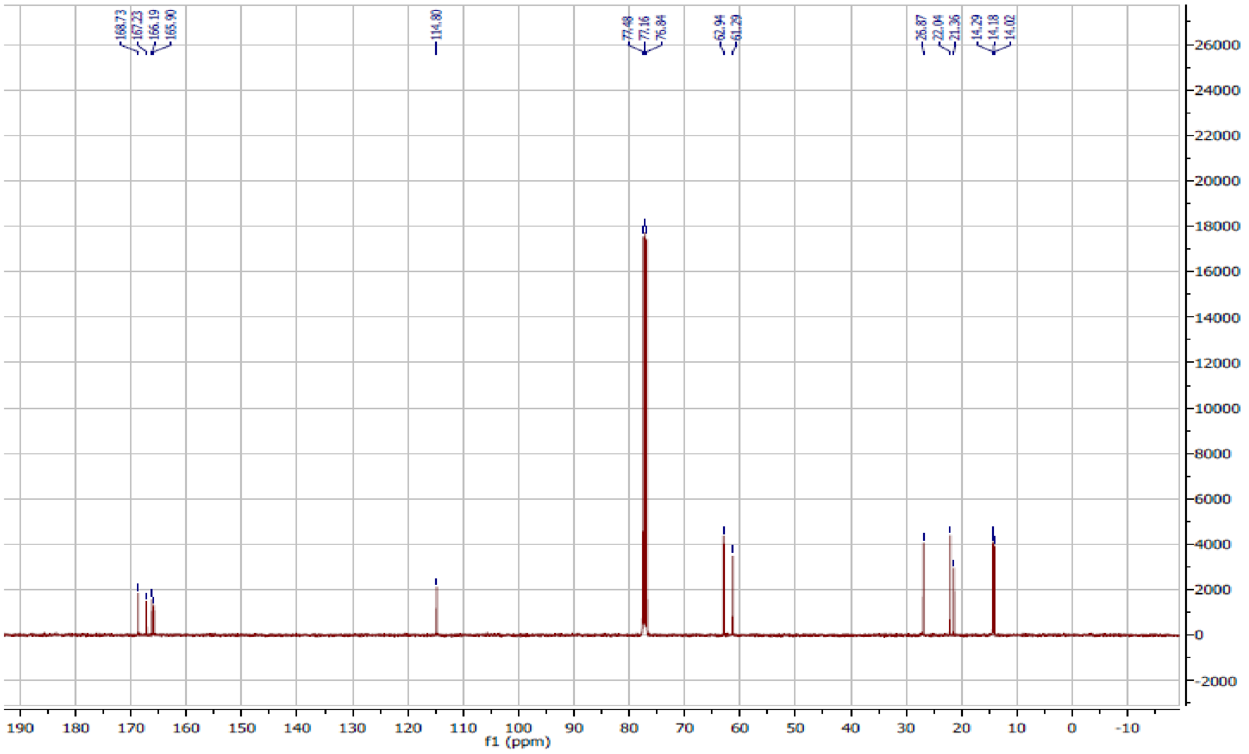

Figure S42: ^{13}C NMR spectrum of compound **22a** ($CDCl_3$).

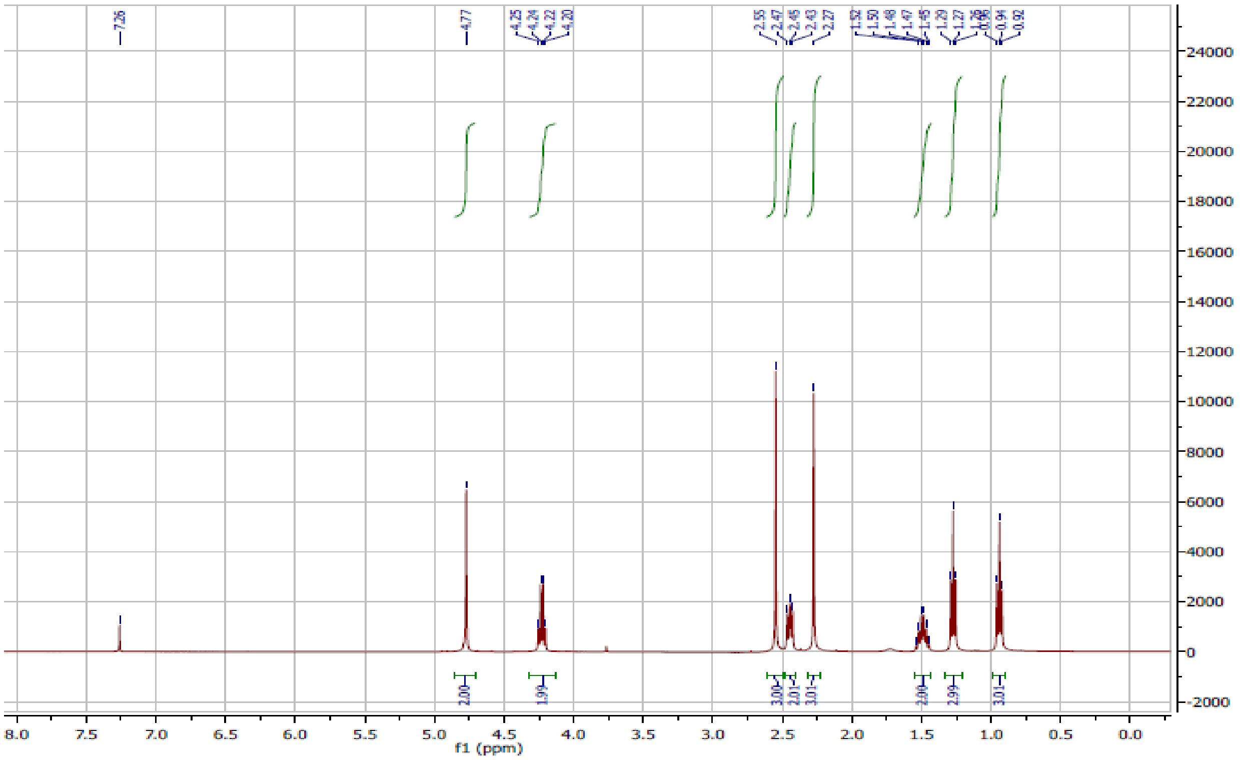

Figure S43: ^{1}H NMR spectrum of compound **22b** ($CDCl_3$).

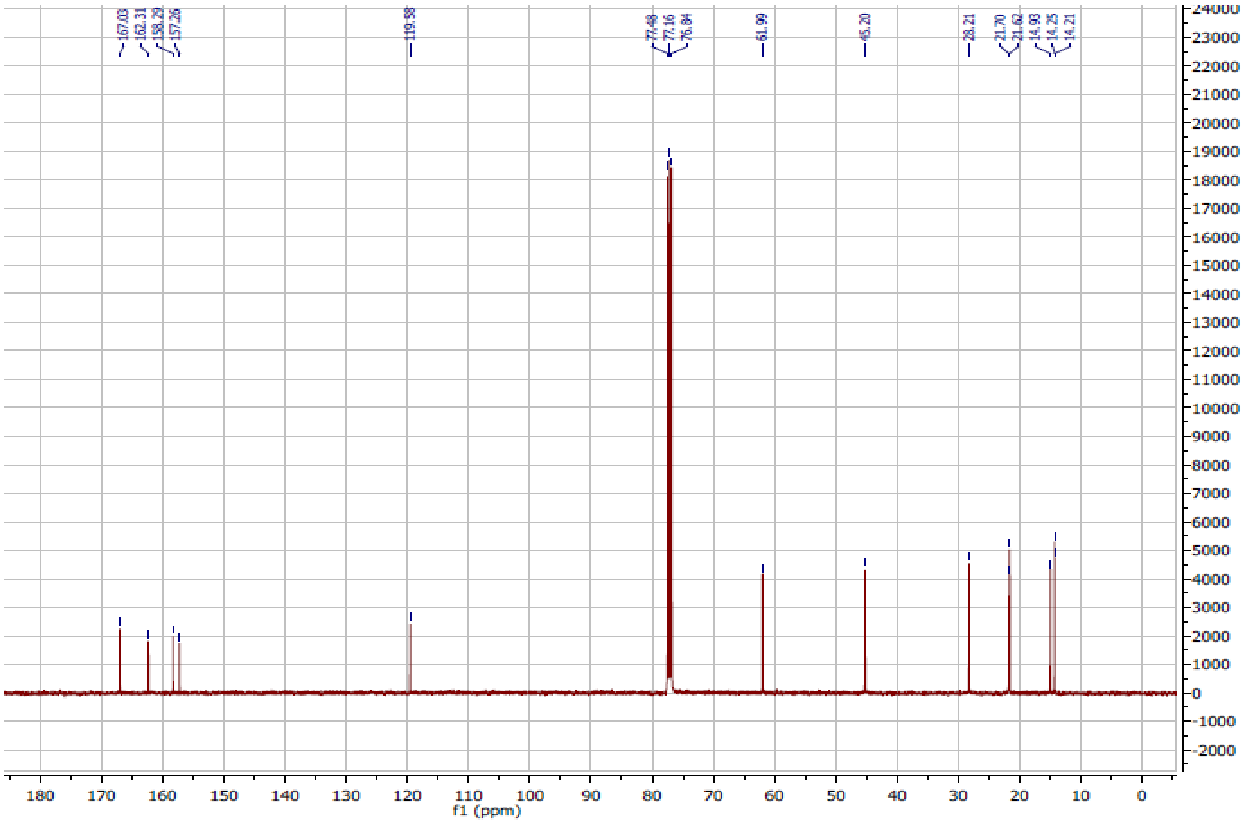

Figure S44: ^{13}C NMR spectrum of compound **22b** ($CDCl_3$).

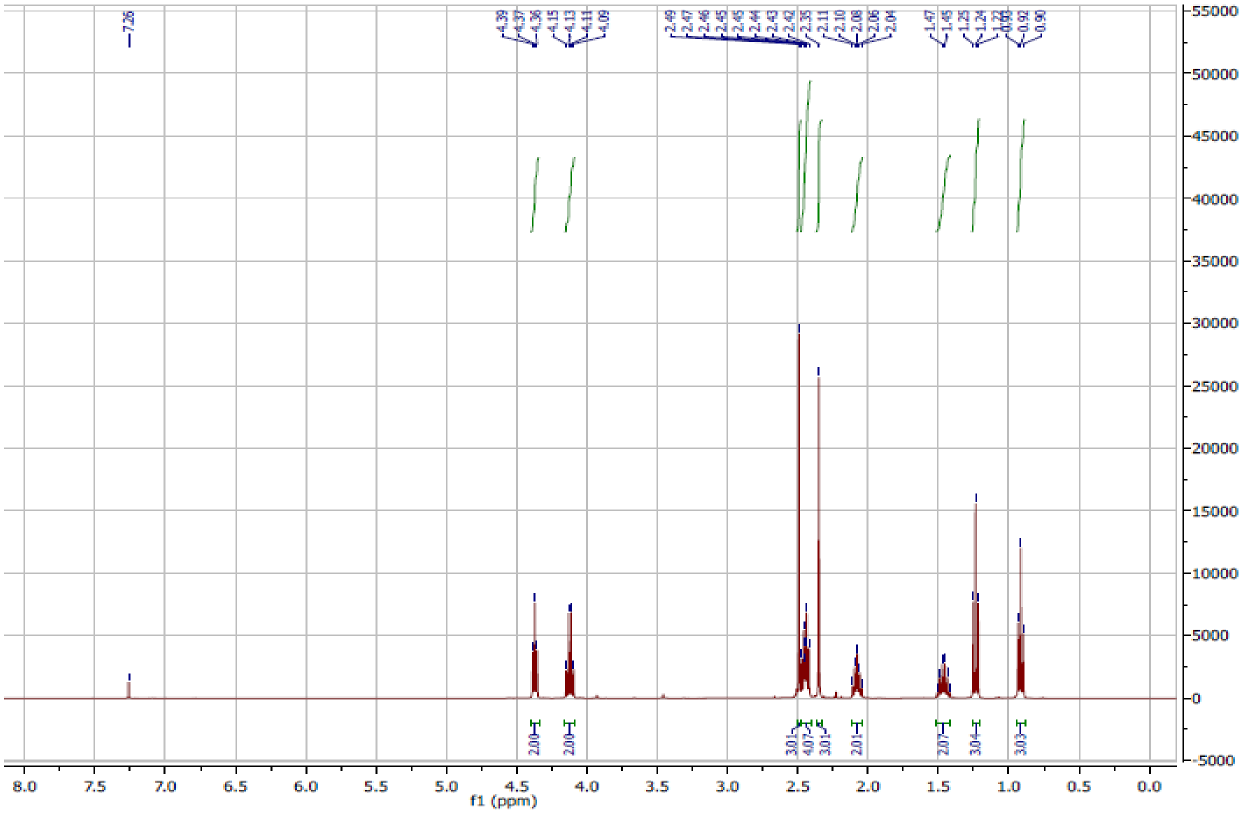

Figure S45: ^{1}H NMR spectrum of compound **23a** ($CDCl_3$).

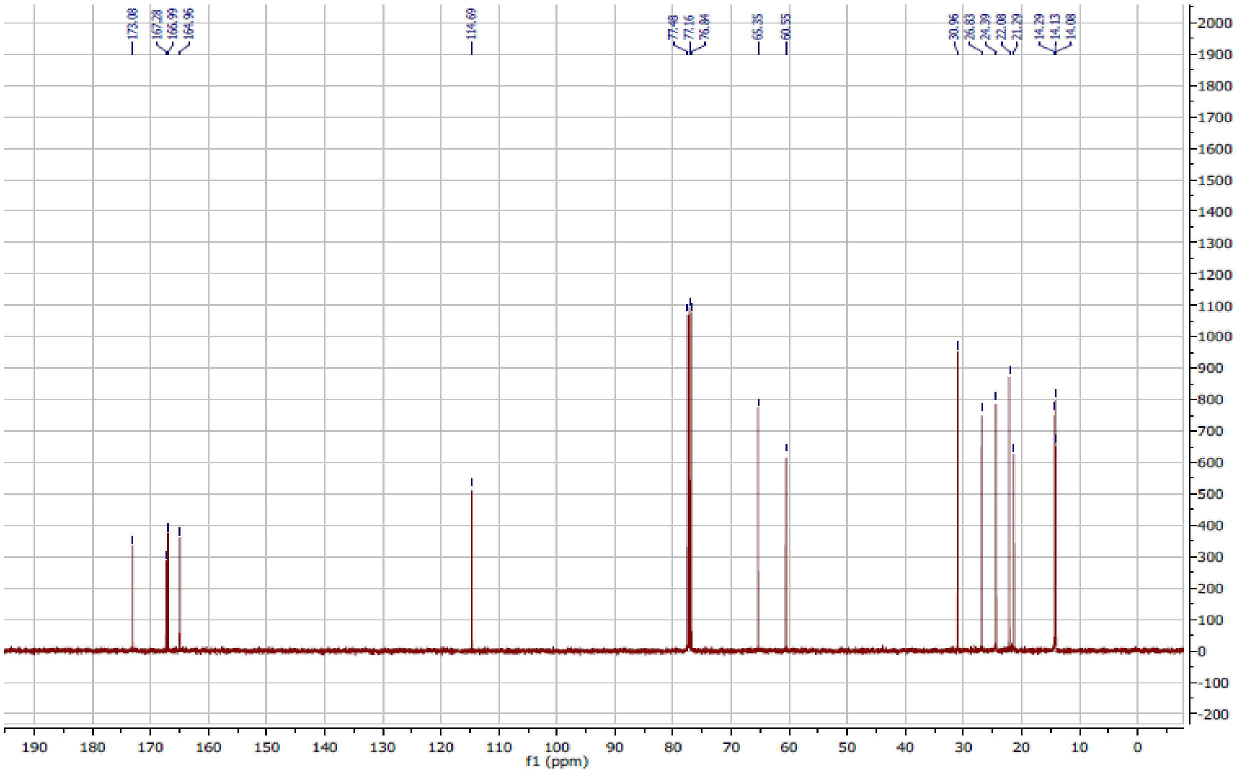

Figure S46: ^{13}C NMR spectrum of compound **23a** ($CDCl_3$).

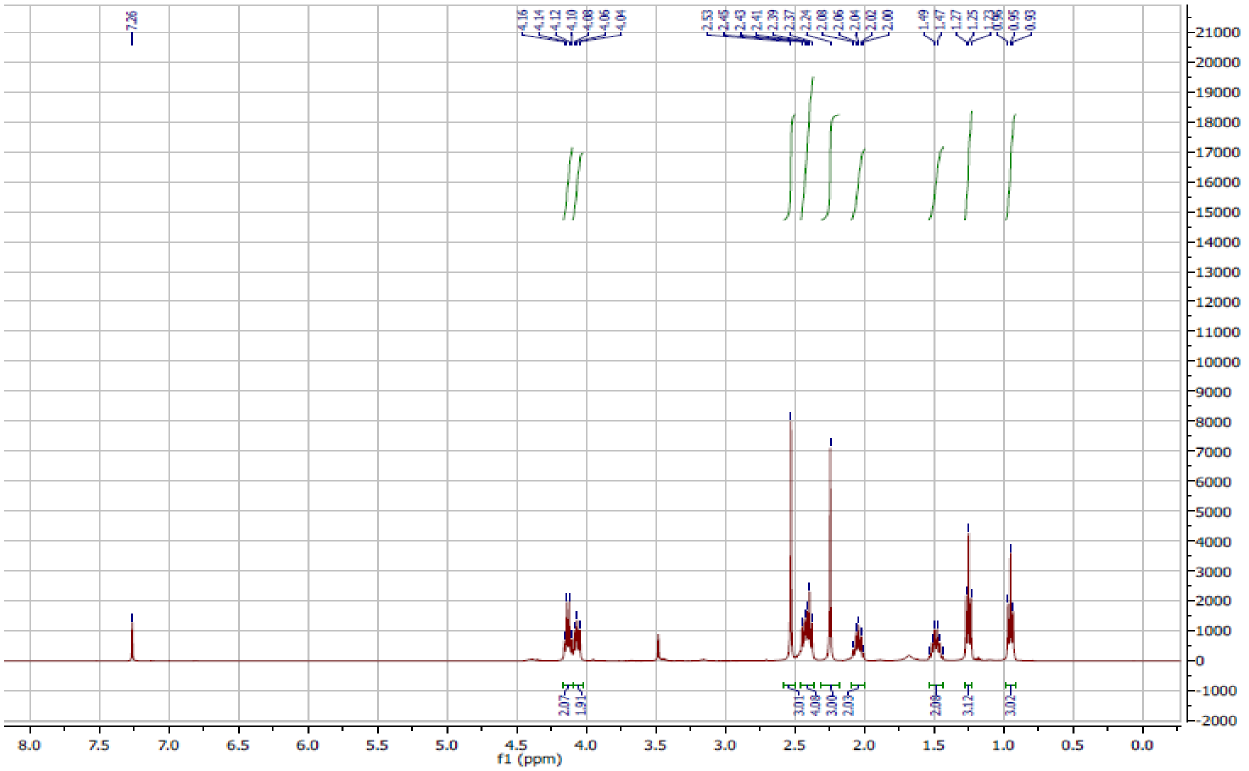

Figure S47: ^{1}H NMR spectrum of compound **23b** ($CDCl_3$).

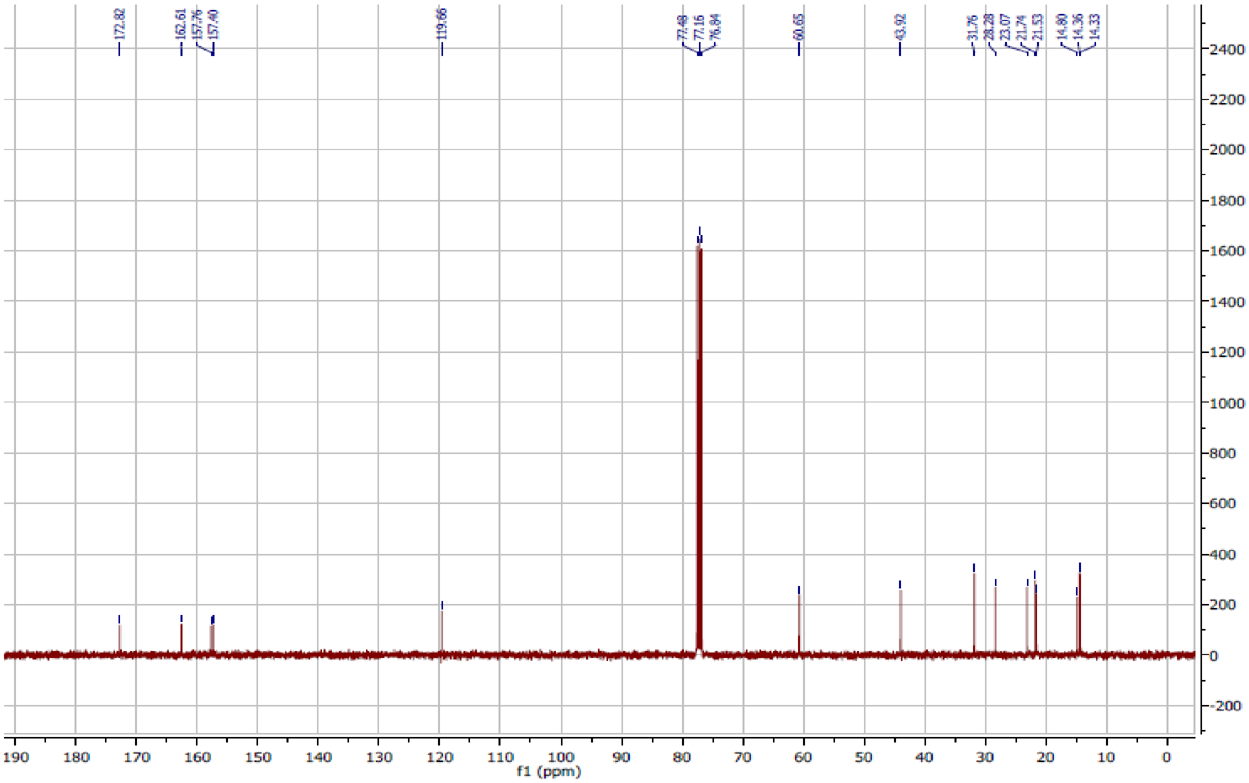

Figure S48: ^{13}C NMR spectrum of compound **23b** ($CDCl_3$).

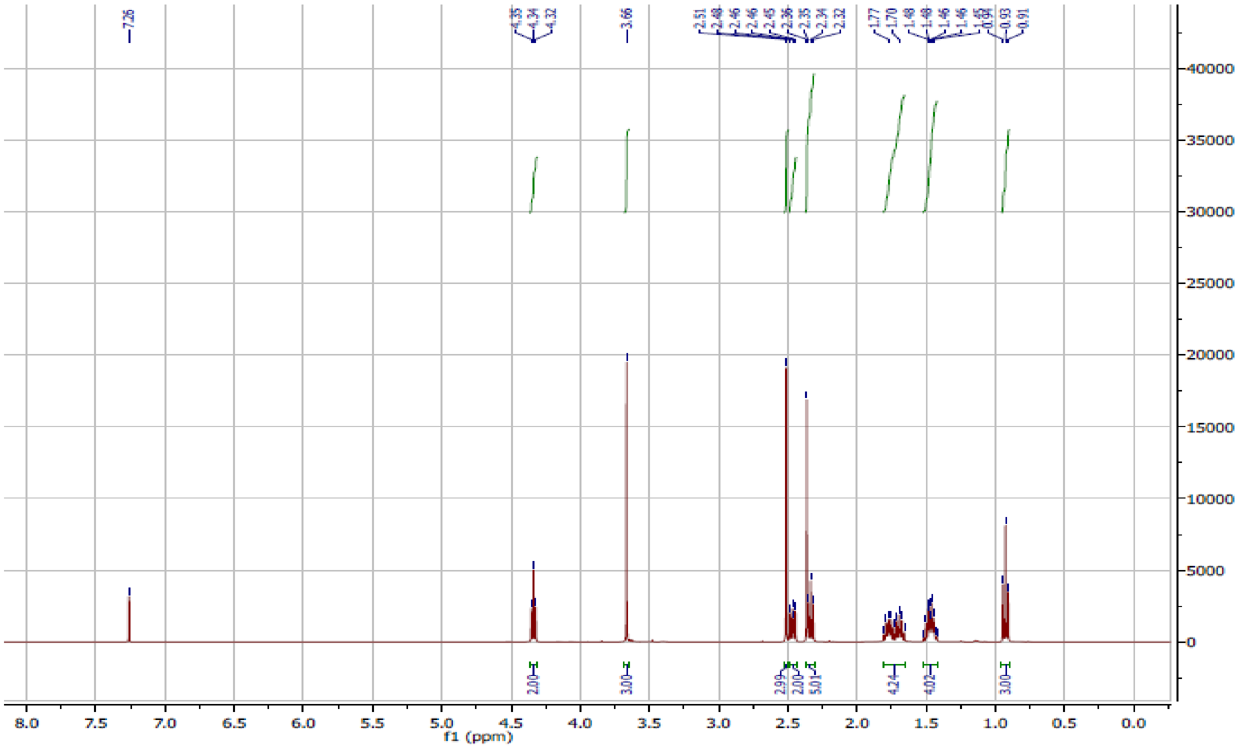

Figure S49: ^{1}H NMR spectrum of compound **24a** ($CDCl_3$).

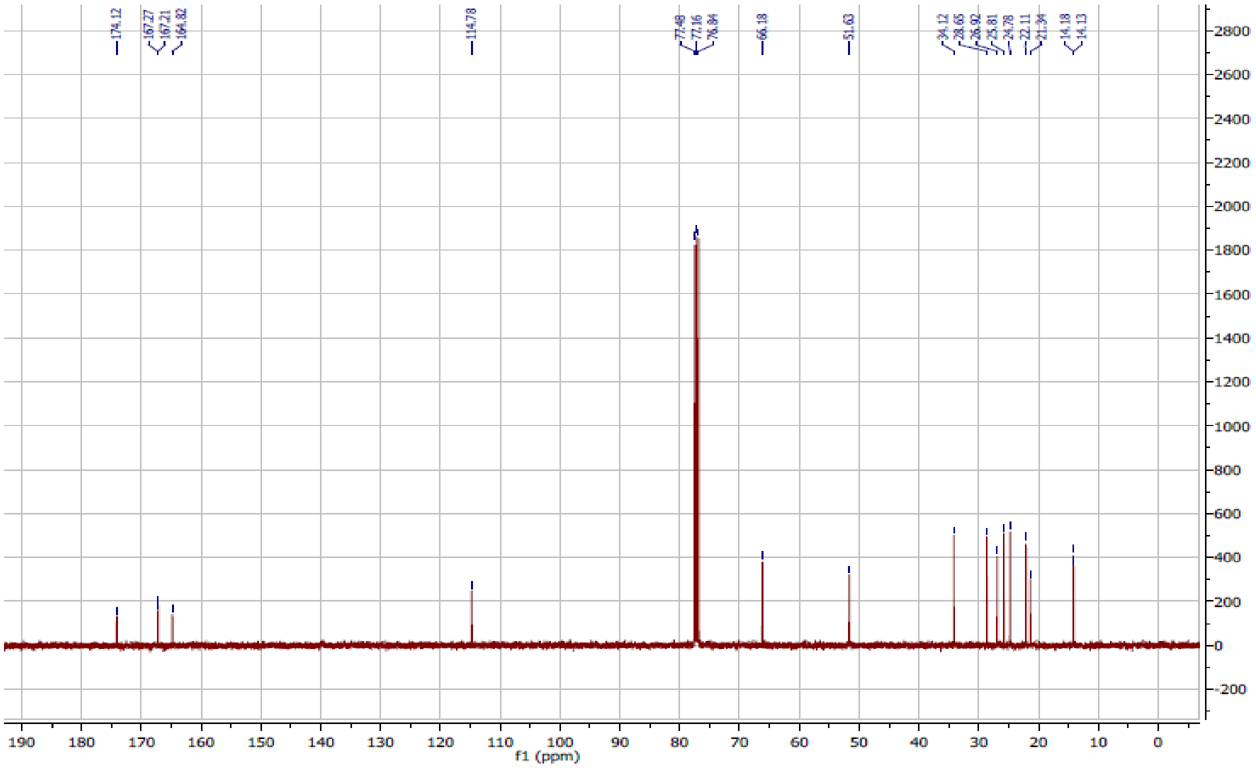

Figure S50: ^{13}C NMR spectrum of compound **24a** ($CDCl_3$).

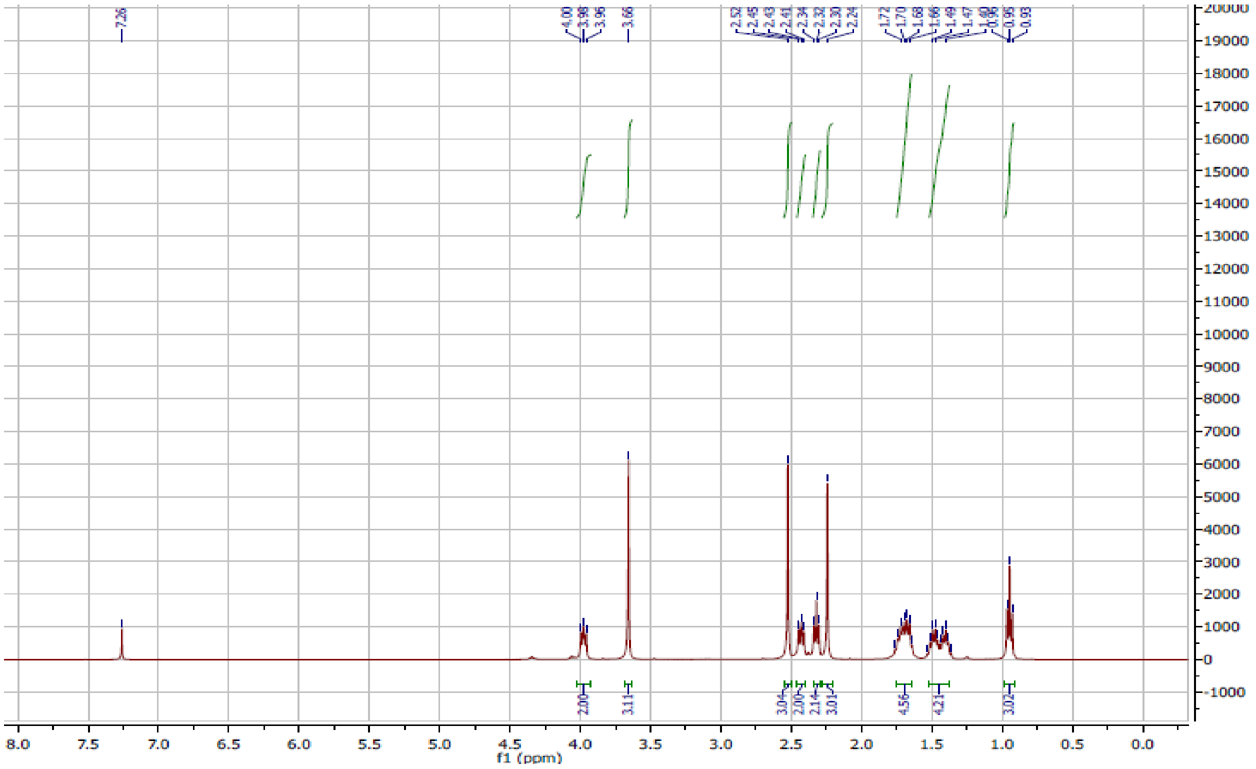

Figure S51: ^{1}H NMR spectrum of compound **24b** ($CDCl_3$).

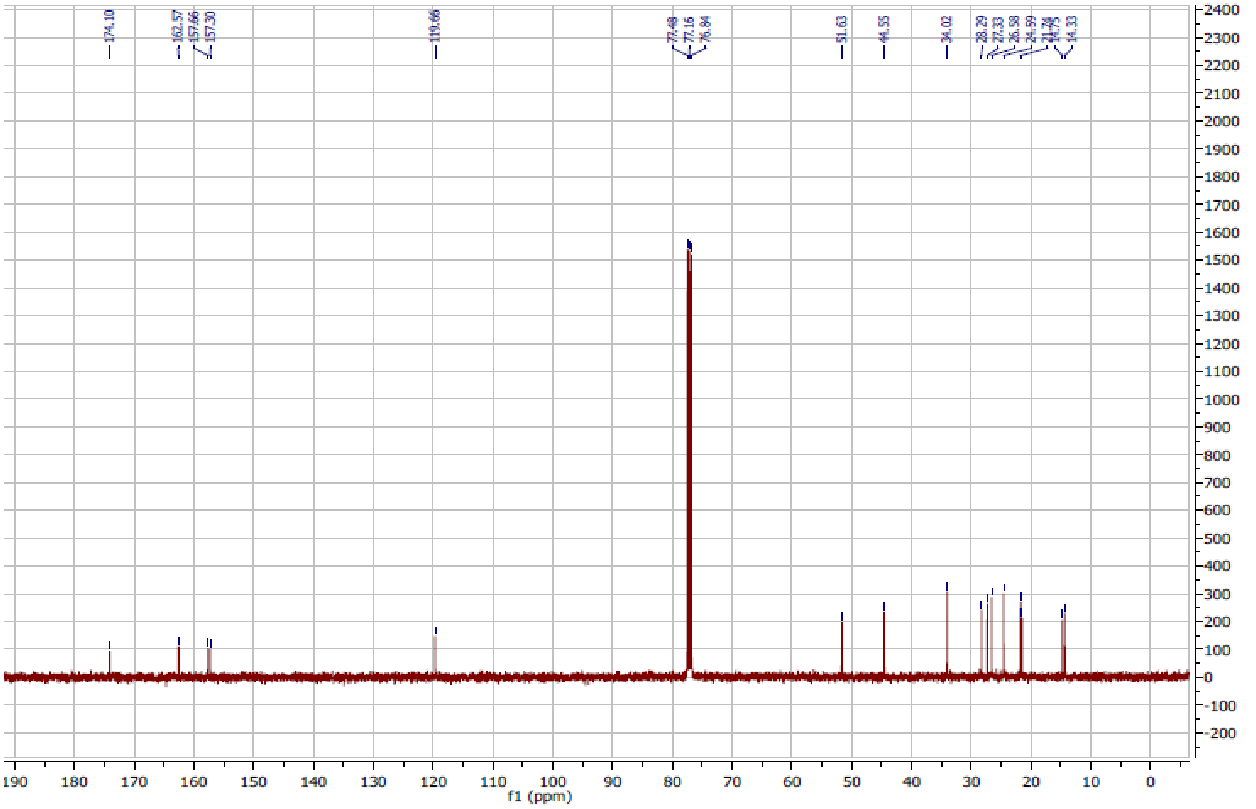

Figure S52: ^{13}C NMR spectrum of compound **24b** ($CDCl_3$).

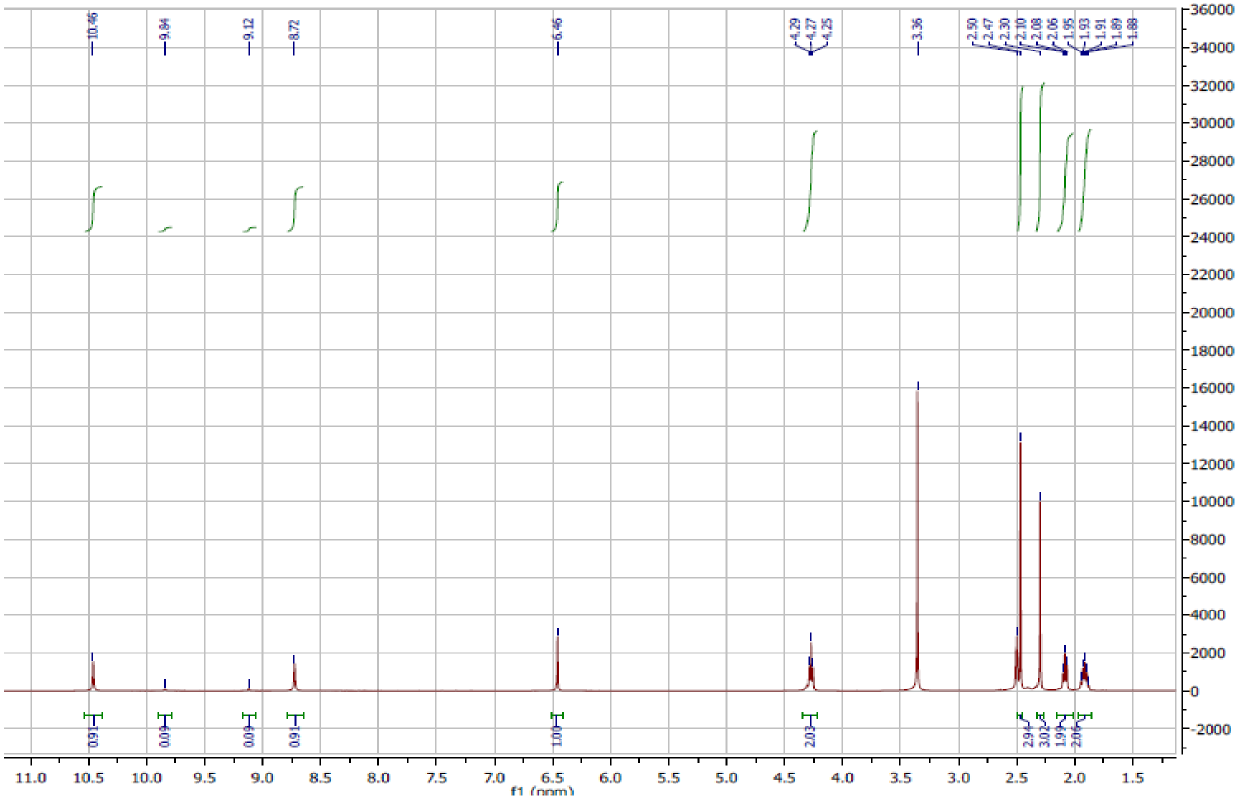

Figure S53: ^{1}H NMR spectrum of compound **25** (DMSO-d_6).

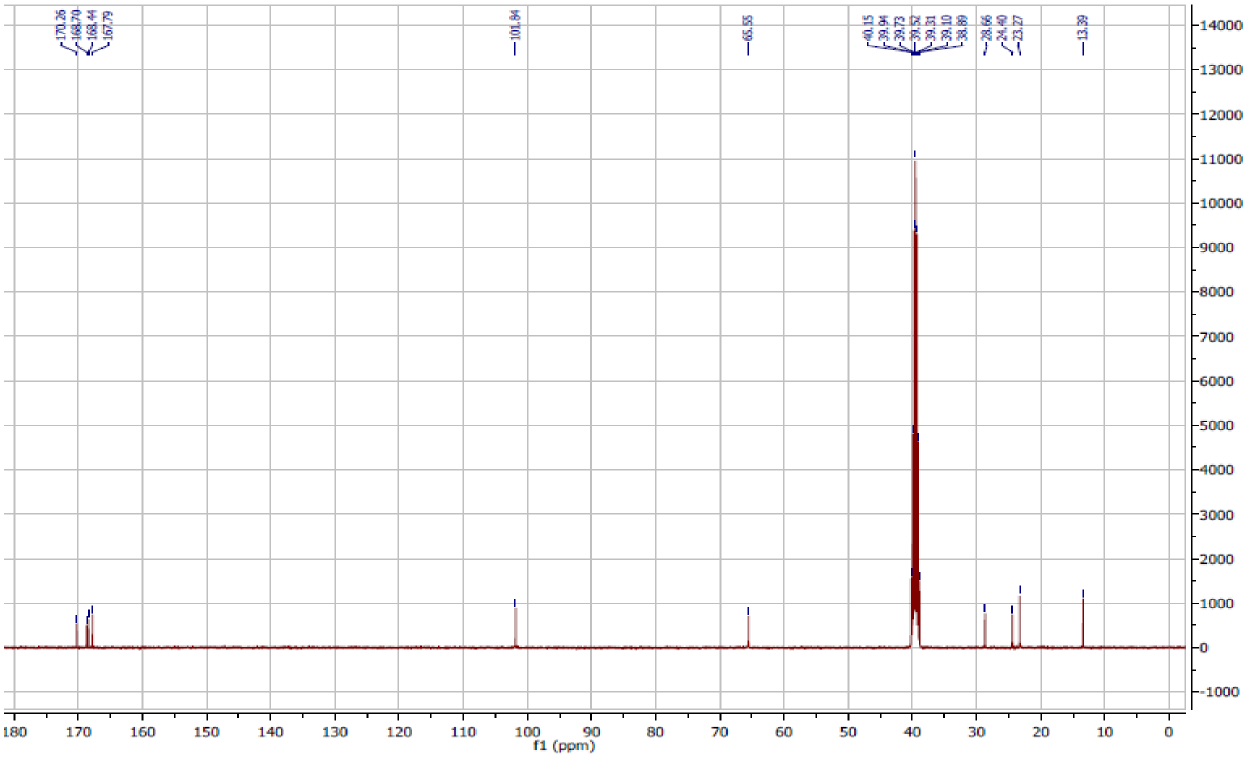

Figure S54: ^{13}C NMR spectrum of compound **25** (DMSO-d_6).

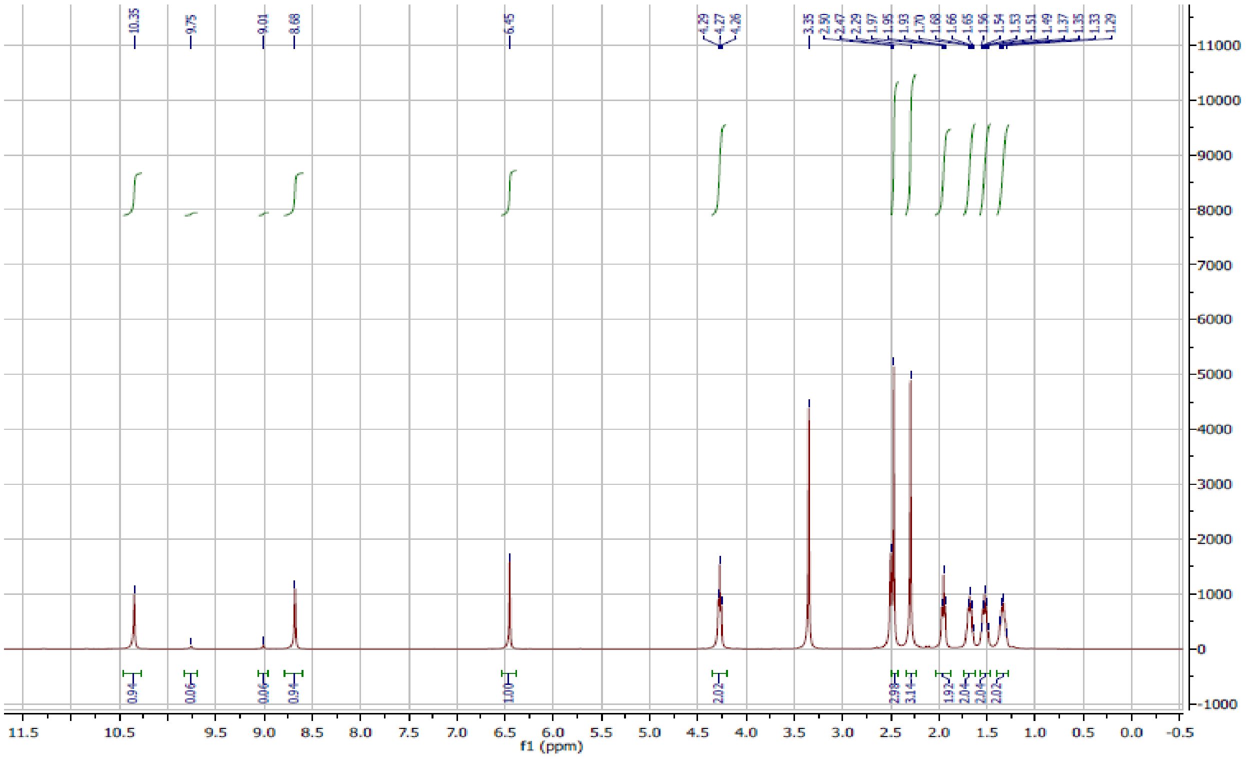

Figure S55: ^{1}H NMR spectrum of compound **26** (DMSO-d_6).

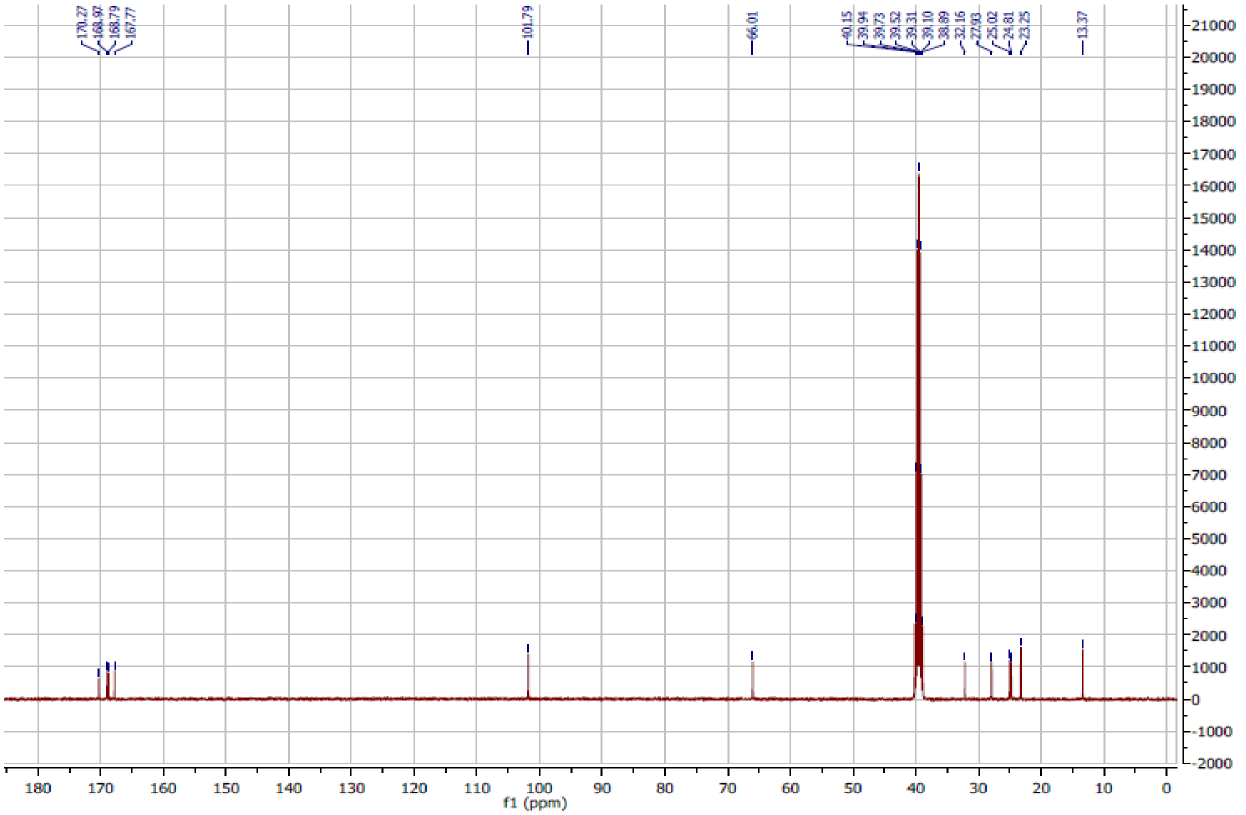

Figure S56: ^{13}C NMR spectrum of compound **26** (DMSO-d_6).

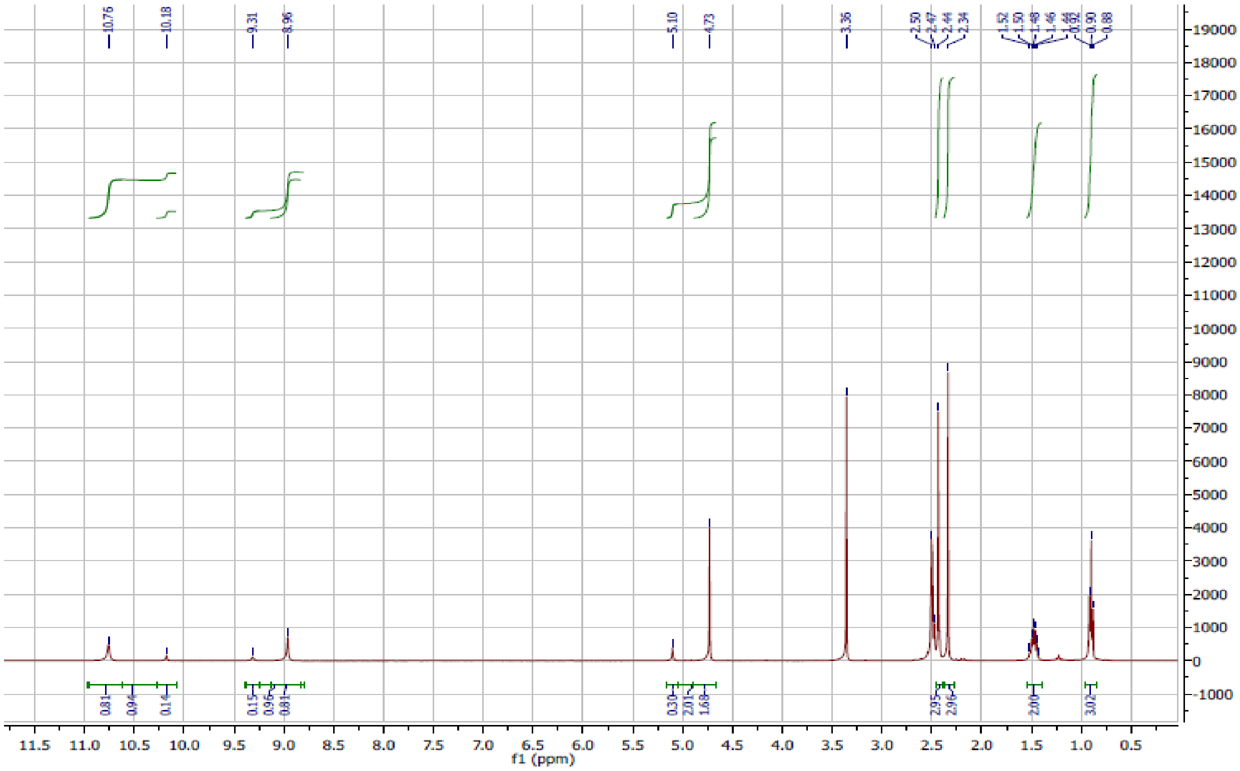

Figure S57: ^{1}H NMR spectrum of compound **27** (DMSO-d_6).

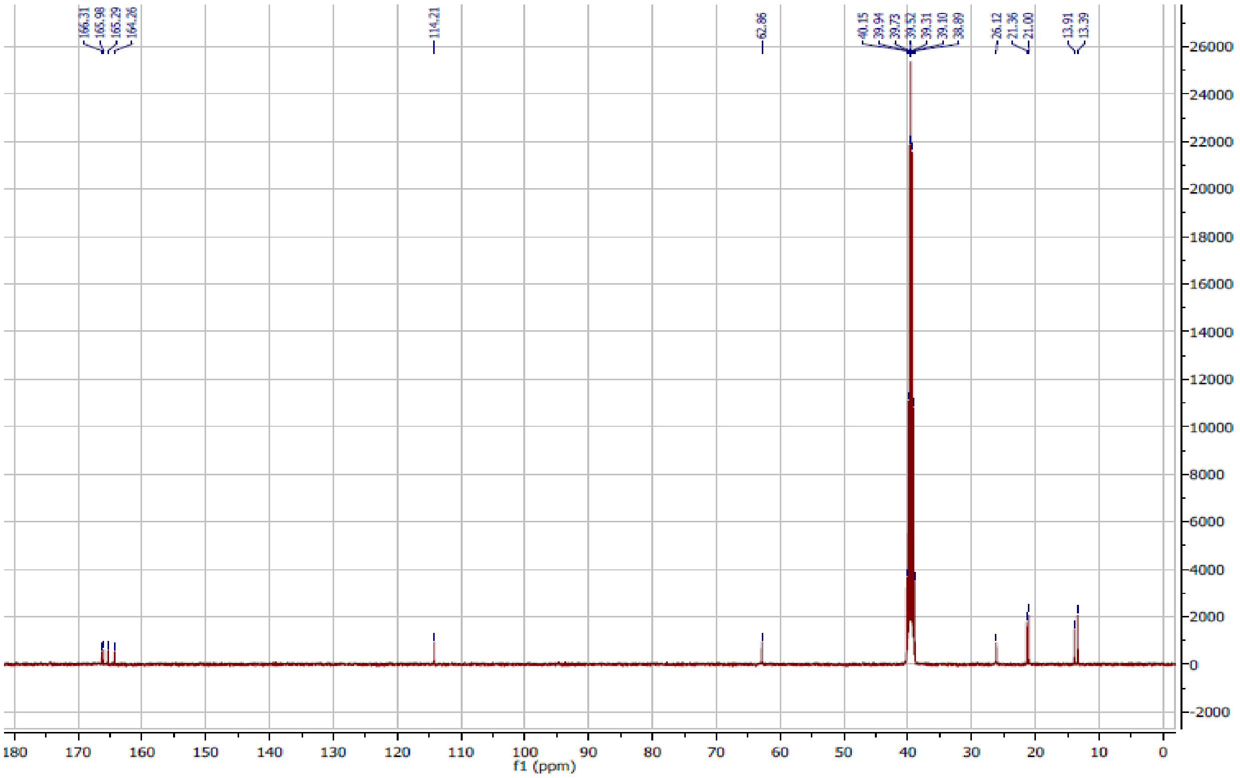

Figure S58: ^{13}C NMR spectrum of compound **27** (DMSO-d_6).

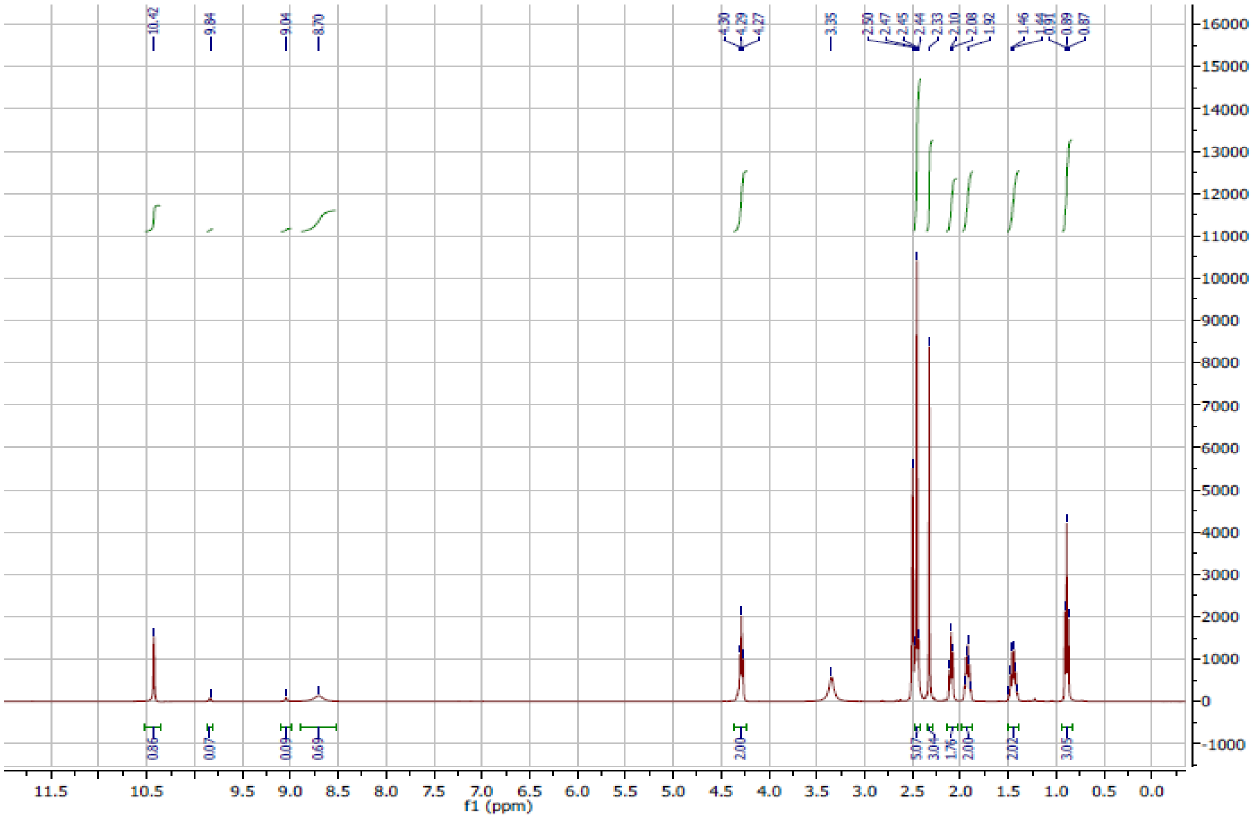

Figure S59: ^{1}H NMR spectrum of compound **28** (DMSO-d_6).

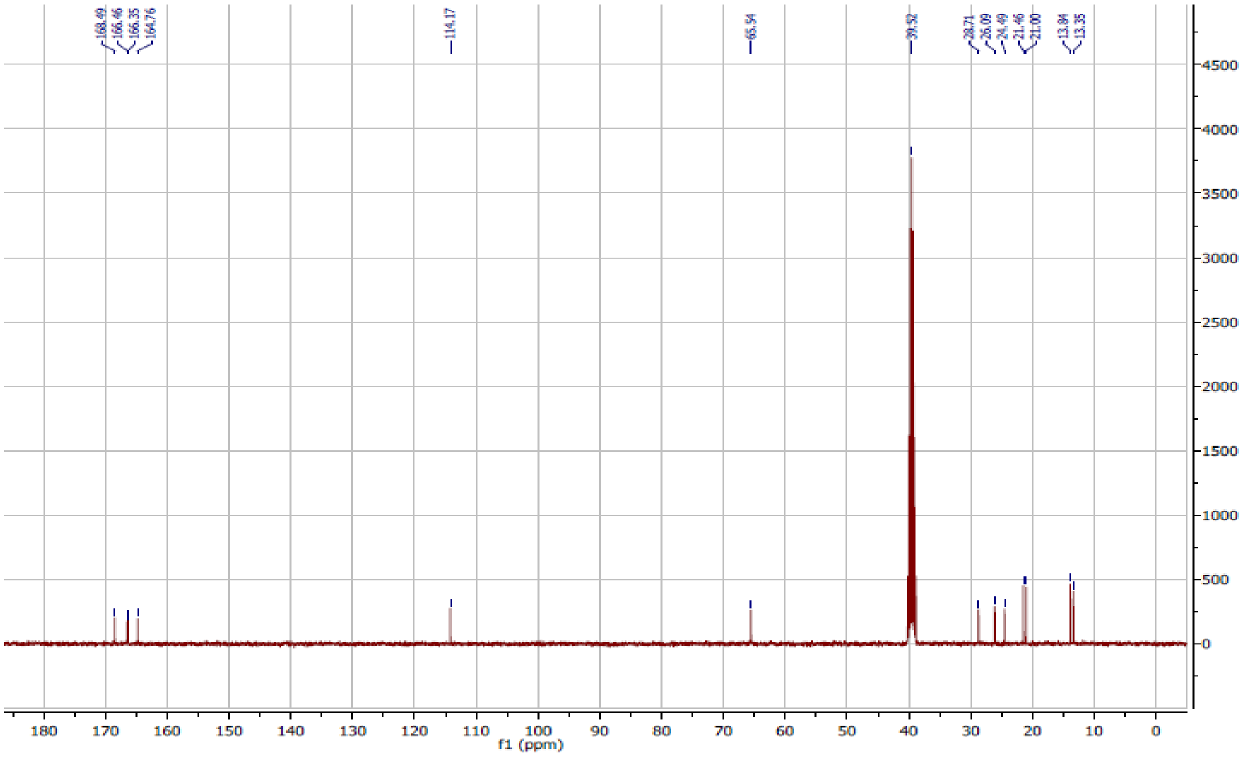

Figure S60: ^{13}C NMR spectrum of compound **28** (DMSO-d_6).

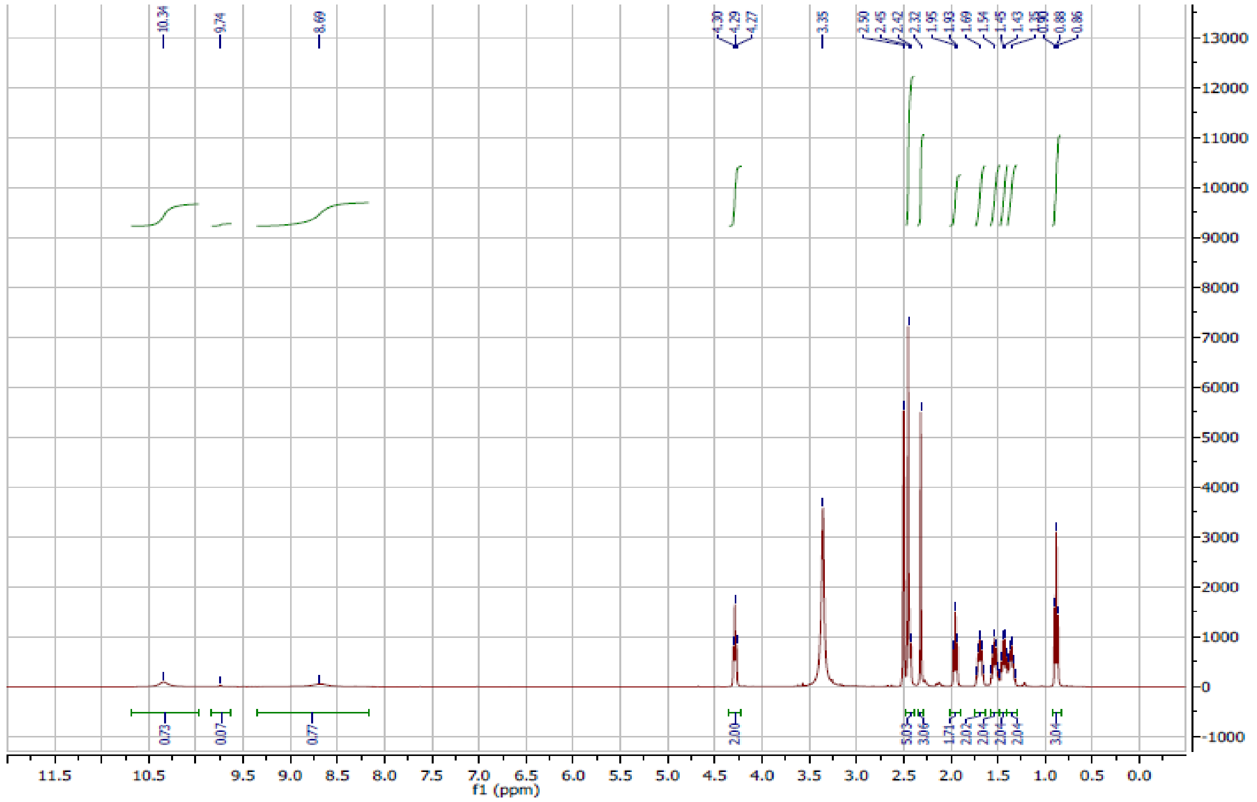

Figure S61: ^{1}H NMR spectrum of compound **29** (DMSO-d_6).

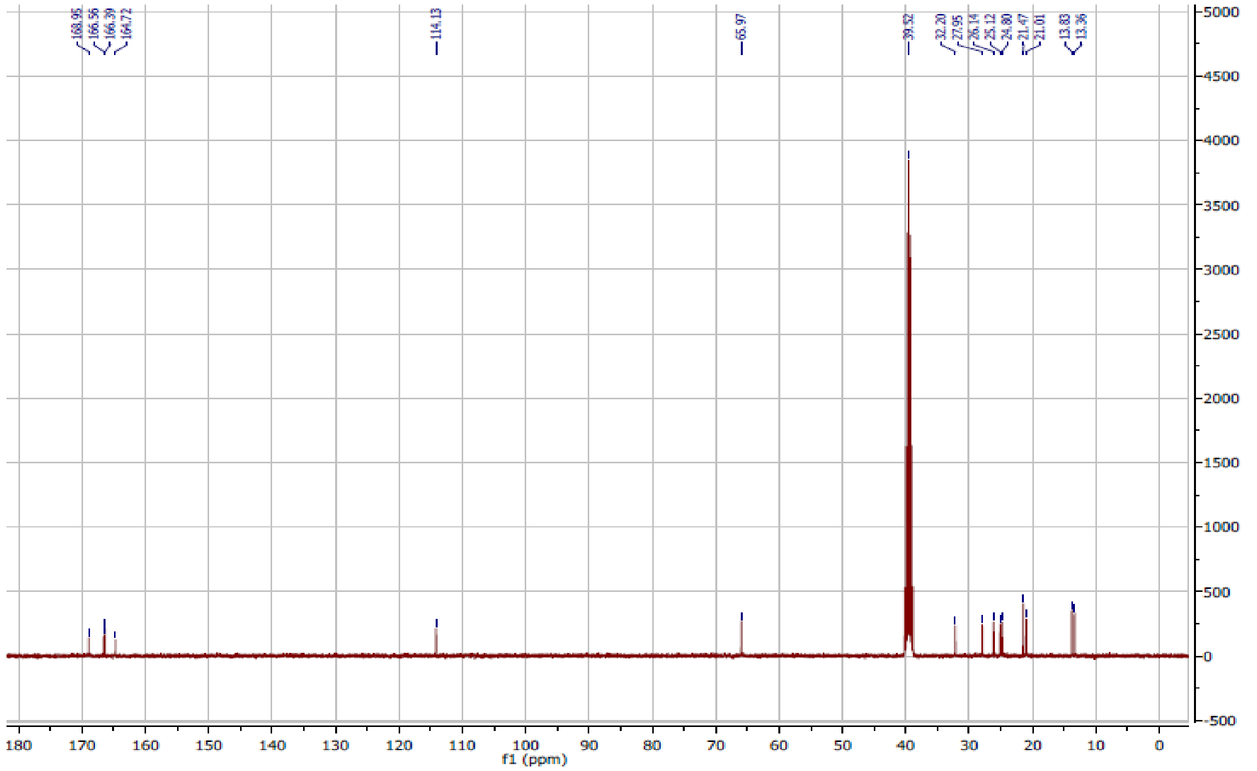

Figure S62: ^{13}C NMR spectrum of compound **29** (DMSO-d_6).

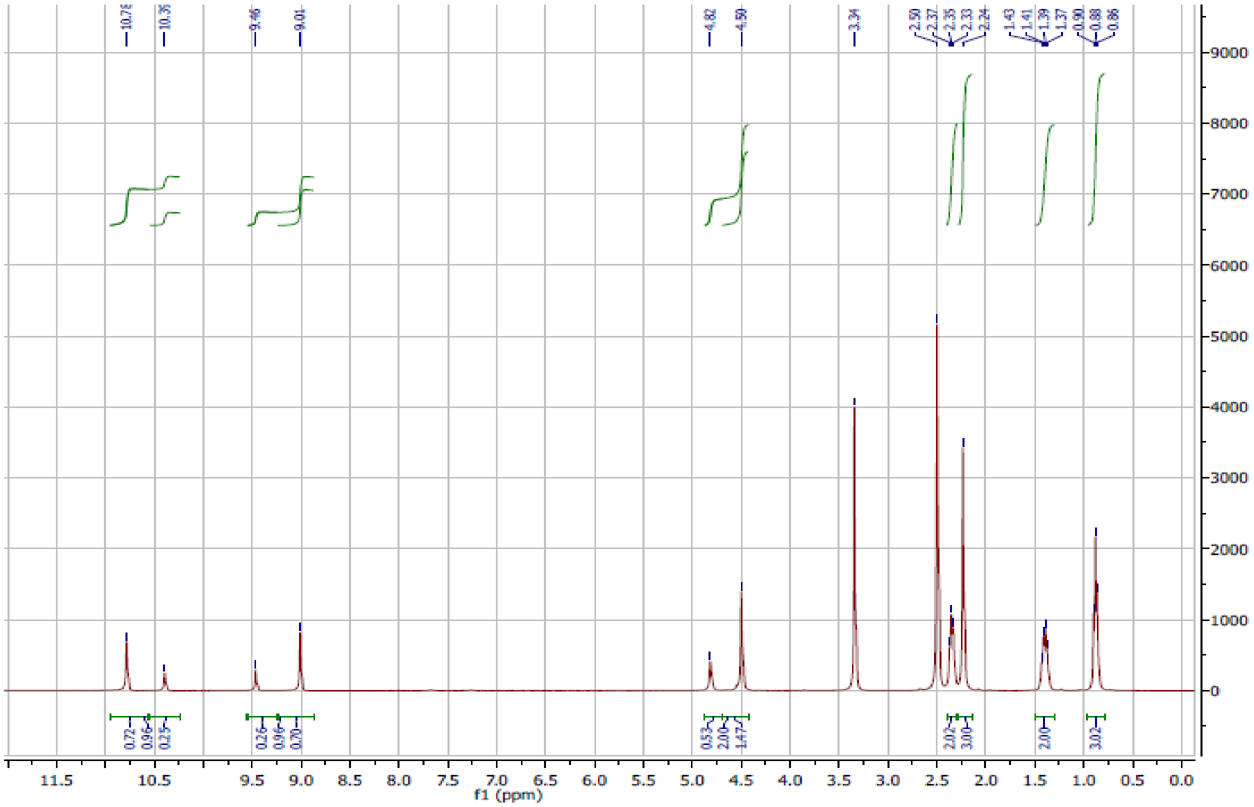

Figure S63: ^{1}H NMR spectrum of compound **30** (DMSO-d_6).

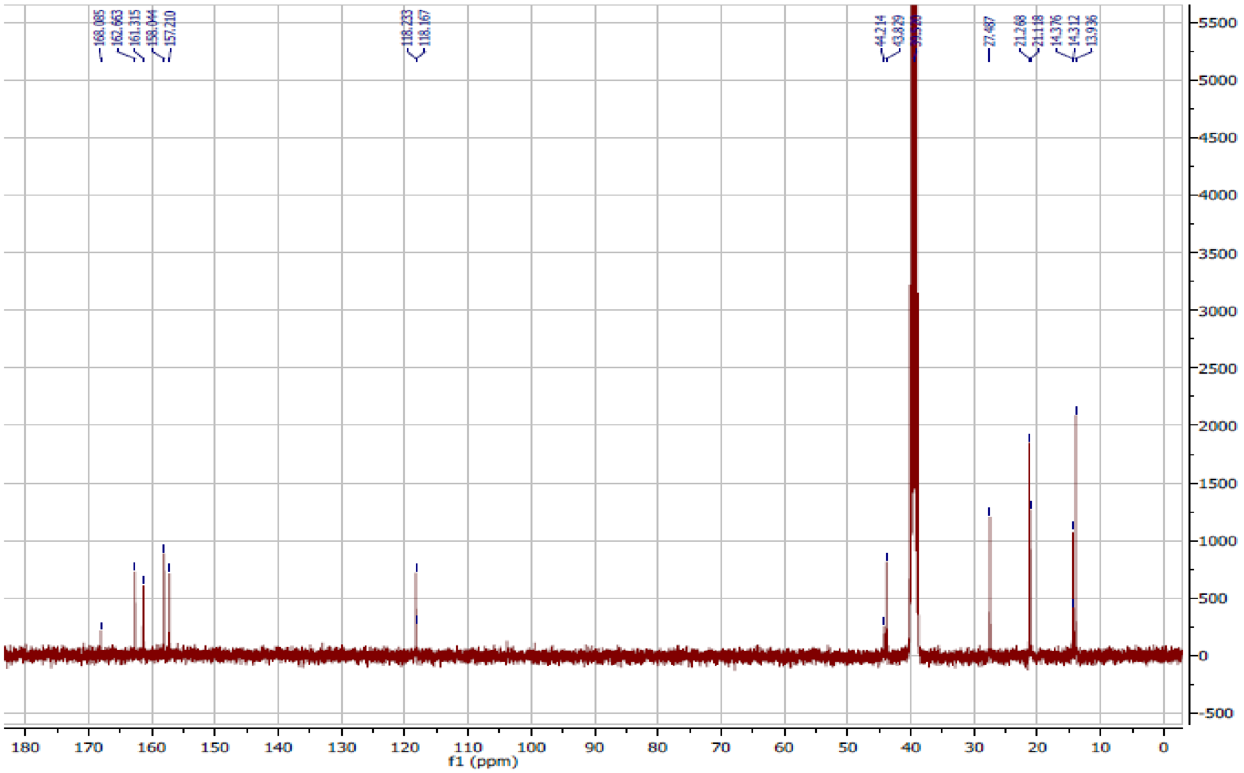

Figure S64: ^{13}C NMR spectrum of compound **30** (DMSO-d_6).

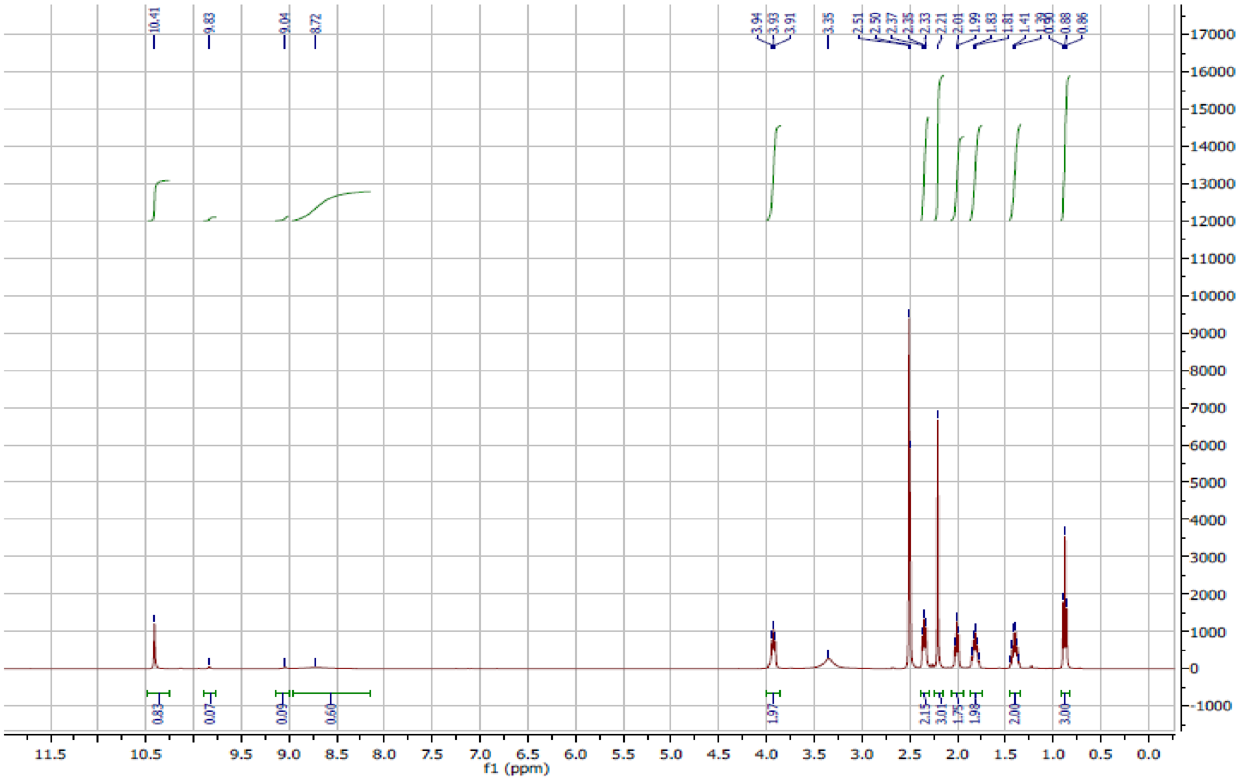

Figure S65: ^{1}H NMR spectrum of compound **31** (DMSO-d_6).

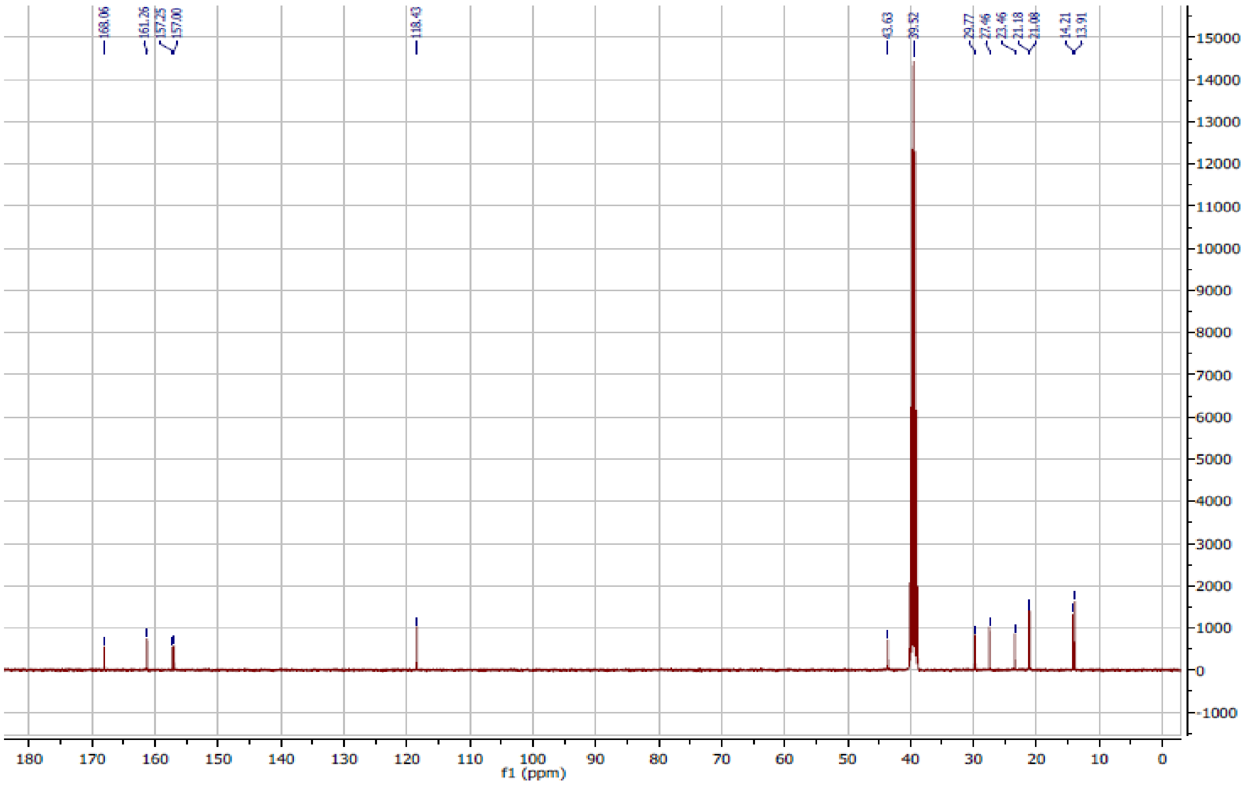

Figure S66: ^{13}C NMR spectrum of compound **31** (DMSO-d_6).
